# Indicators for monitoring maternal and neonatal quality care: a systematic review

**DOI:** 10.1186/s12884-019-2173-2

**Published:** 2019-01-11

**Authors:** Pedro J. Saturno-Hernández, Ismael Martínez-Nicolás, Estephania Moreno-Zegbe, María Fernández-Elorriaga, Ofelia Poblano-Verástegui

**Affiliations:** Centro de Investigación en Evaluación y Encuestas, Instituto Nacional de Salud Pública, Universidad No. 655 Colonia Santa María Ahuacatitlán, C.P 62100 Cuernavaca, Morelos Mexico

**Keywords:** Indicators, Maternal health, Monitoring, Neonatal health, Quality of health care, Systematic review

## Abstract

**Background:**

Research and different organizations have proposed indicators to monitor the quality of maternal and child healthcare, such indicators are used for different purposes.

**Objective:**

To perform a systematic review of indicators for the central phases of the maternal and child healthcare continuum of care (pregnancy, childbirth, newborn care and postpartum).

**Method:**

A search conducted using international repositories, national and international indicator sets, scientific articles published between 2012 and 2016, and grey literature. The eligibility criteria was documents in Spanish or English with indicators to monitor aspects of the continuum of care phases of interest.

The identified indicators were characterized as follows: formula, justification, evidence level, pilot study, indicator type, phase of the continuum, intended organizational level of application, level of care, and income level of the countries. Selection was based on the characteristics associated with scientific soundness (formula, evidence level, and reliability).

**Results:**

We identified 1791 indicators. Three hundred forty-six were duplicated, which resulted in 1445 indicators for analysis. Only 6.7% indicators exhibited all requirements for scientific soundness. The distribution by the classifying variables is clearly uneven, with a predominance of indicators for childbirth, hospital care and facility level.

**Conclusions:**

There is a broad choice of indicators for maternal and child healthcare. However, most indicators lack demonstrated scientific soundness and refer to particular continuum phases and levels within the healthcare system. Additional efforts are needed to identify good indicators for a comprehensive maternal and child healthcare monitoring system.

**Electronic supplementary material:**

The online version of this article (10.1186/s12884-019-2173-2) contains supplementary material, which is available to authorized users.

## Background

Maternal and child health (MCH) has been a priority public health problem for decades [1]. Since the 1990s, the international community has implemented important initiatives to reduce the morbidity and mortality of mothers and newborn infants. We highlight the Millennium Development Goals (MDG) [2], as well as the current Sustainable Development Goals (SDG) that reinforce good health and well-being for women and children (Goal 3), coupled with the target of universal health coverage [3] and reflected in the renewed Global Strategy for Women’s, Children’s and Adolescents’ Health (2016–2030) [4]. MCH care frameworks to integrate and improve health system performance have simultaneously been proposed as the so-called *continuum of care* for maternal, newborn, and child health. This *continuum* has been presented as a rallying call to reduce the toll of maternal, newborn and child deaths, and has reached a solid international acceptance [5].

The initial main improvement strategy has focused on access to health services and increasing the number of births in health facilities, as well as the number of births attended by qualified health personnel. However, the improvement in accessibility has not yielded a similar reduction in the morbidity and mortality of mothers and newborns, thus bringing to the foreground the importance of the quality of care received [6, 7] and its measurement, which indicate the period around childbirth as the most important.

The emphasis on quality in several international initiatives has favored the construction and measurement of indicators [8–12]. Indicators of quality as “measurement tools that can be used to monitor, evaluate and improve the quality of patient care, organization and support services that affect patient outcomes” [13]. The consensus is that measurement of performance is essential to support improvement and accountability. However, this has unleashed a multitude of uncoordinated and often duplicative measurement and reporting initiatives [12]. Different international organizations, civil society groups, academics, and countries have proposed indicators. These indicators are used for different purposes and often examine only a component of the attention; rarely are the different initiatives integrated and coordinated [8, 9, 12, 14]. As a result, in the current situation, many initiatives co-exist; however, the total number and characteristics of the available indicators, as well as the particular technical components on which they were built, remain unknown [12, 14].

In this context, it appears relevant to perform a search and systematic review of the existing indicators for the most important components of the continuum of care (pregnancy, childbirth, newborn and puerperium), using explicit and homogeneous criteria in relation to their scientific soundness and unambiguous definitions for their application according to the levels of responsibility within the health system.

## Methods

This study was conducted in two stages: 1) a systematic search for maternal and neonatal quality of care indicators related to four of the phases of the *continuum of care:* pregnancy, childbirth, puerperium and newborn up to the first two months of life; 2) characterization, classification and selection of the indicators with explicit criteria on scientific soundness, including complete description, validity with explicit level of evidence, tested reliability and feasibility, and proposed applicability.

### Systematic search strategy and eligibility criteria

The searches were conducted using four sources: 1) international repositories of indicators; 2) compendiums or established national and international indicator sets; 3) grey literature; and 4) scientific articles, from 2012 to August 2016. We considered only indicators in Spanish and English. The search in repositories was conducted in the *National Quality Measures Clearinghouse* (NQMC) [15]; *National Quality Forum* (NQF) [16] and MEASURE Evaluation [17]. The selected compendiums or indicator sets were as follows: *Inpatient Quality Measures* [18] and *Core Measures* [19] of *The Joint Commission*; indicators of *Medicare and Medicaid Services* [20]; *General Practice Quality and Outcomes Framework* [21] and *Clinical Commissioning Group Outcomes Indicator Set* [22]; *Key indicators of the national health system* of Spain [23]; *National quality indicators in the health system* of Mexico [24]; *European Core Health Indicators* [25]; and indicators of the Organization for the Economic Cooperation and Development (OECD) [26]. The search of scientific articles was conducted in the *PubMed* database, whereas the grey literature search was performed through the search engine *Google.* The search terms used in the indicator compendiums, sets and repositories were as follows: *Neonatal*; *Newborn; Neonate; Infant; Premature; Preterm; Birth; Childbirth; Delivery; Labor; ; Natal; Postnatal; Perinatal; Prenatal; Partum; Postpartum; Peripartum; Intrapartum; Prepartum; Antepartum; Pregnancy; Pregnant; Maternal; Gestation;* and *Gestational*. The *PubMed* search strategy is in the Additional file 1. In Google, we used the terms “*maternal health indicators*”, “*newborn care indicators*”, “*birth indicators*”, “*delivery indicators*”, and “*pregnancy control indicators*”. In all searches, the homologous terms in Spanish were also employed.

To select scientific articles, we initially reviewed the titles and abstracts of all publications obtained in the search and maintained the articles that included indicators related to some aspect of the targeted phases of the continuum. Articles that addressed other phases of the continuum, did not describe the use of indicators, or were part of one of the analyzed compendiums and repertoires were discarded. In the grey literature, we extracted documents that included a set of indicators on MCH endorsed by organizations with international recognition, such as the WHO, UNICEF, and OECD. Additional file 2 shows the complete list of references used in the research by source (repositories, compendiums, scientific articles and grey literature).

### Characterization and classification of the indicators

**Fig. 1:**
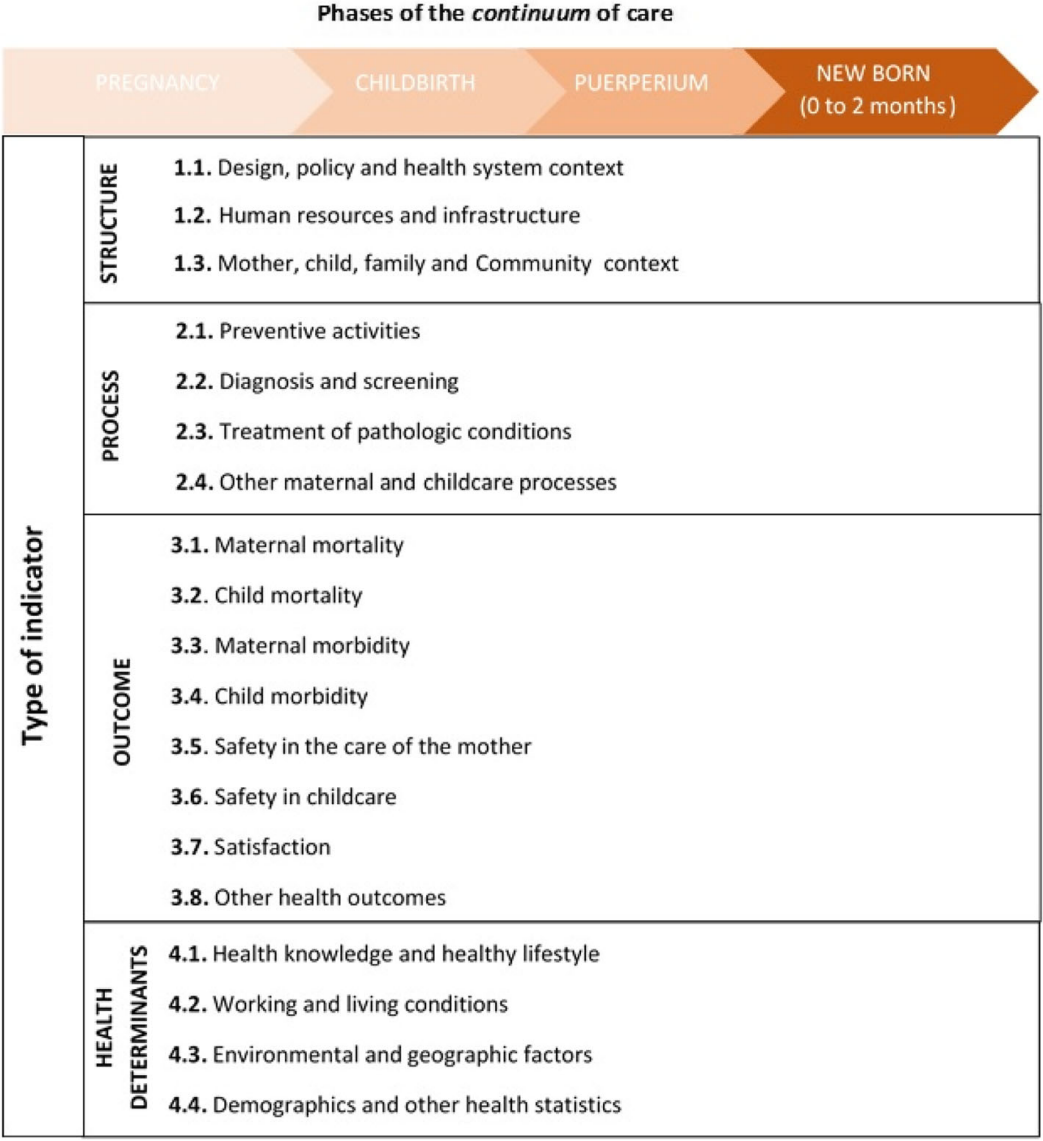
Framework for grouping indicators according to type, aspect of care measured, and phase of the maternal and childcare continuum

The reference framework is the phases of the continuum of care of interest, and the type of indicator and aspect of care measured [5, 27–29], as depicted in Fig. 1. To analyse, characterize and select them, we use the variables described in Table 1. The indicators found in the search were entered into a database that identified: full name; numerator and denominator or equivalent (format type if/then) [30]; type of indicator according to the Donabedian model [27] (structure, process, outcome) with an additional option for those not directly related to health care (determinants of health and demographic and social statistics) [28, 29]; phase of the *continuum* to which they refer [5]; referenced scientific justification; explicit level of evidence; existence of pilot study; organizational level of application within the health system; level of care for which the indicators are used; and the income level [31] of the country or countries for which they are proposed (Table 1).

**Table 1:** Variables used for the characterization of indicators

Variable	Meaning
a. Title	Name of the indicator
b. Formula	* Numerator * Denominator or equivalent (If/then format)
c. Type of indicator	* Structure * Process * Outcome * Health determinants and social and demographic statistics
d. Phase of c*ontinuum*	1. Pregnancy 2. Childbirth 3. Postpartum 4. Newborn (up to 2 months of age) 5. Children from 0 to 18 years of age 6. Other (more than two stages of *continuum*)
e. Referenced scientific justification	Based on evidence (Yes/No)
f. Explicit level of evidence	The quality of the scientific evidence used is explicitly assessed or described
g. Pilot study	Description of the implementation of a pilot study for practical validation of the indicator
h. Organizational level for application	Level of responsibility within the health system for which the indicator is proposed * In a service or specific unit within a health facility * In a health facility * At supra-institutional level (region, State or national)
i. Level of care	Level of care that are or can be used as indicators * Primary care level * Hospital care (second or third level of care)
j. Income level of the country for which the indicator is proposed	Income level of the country in which the use of the indicator is intended, according to the World Bank classification * High income * Medium and low income * High, medium and low income (all)

Two researchers independently extracted information on each indicator and discussed the discrepancies. In cases of unsolved discrepancies, a third senior researcher was consulted.

The analyzed indicators were grouped in relation to the intersection of the phase of the continuum to which they relate, and the main characteristics of the indicator: type of indicator and the activity or measurement objective (Fig. 1), level of care, application level, and income level of the country of intended use. In relation to the continuum, the indicators that applied to more than one of the four targeted phases were classified as “other”, whereas the indicators that also applied to infants older than 2 months of age (up to 18 years old) were considered a different category.

### Selection of indicators

**Fig. 2:**
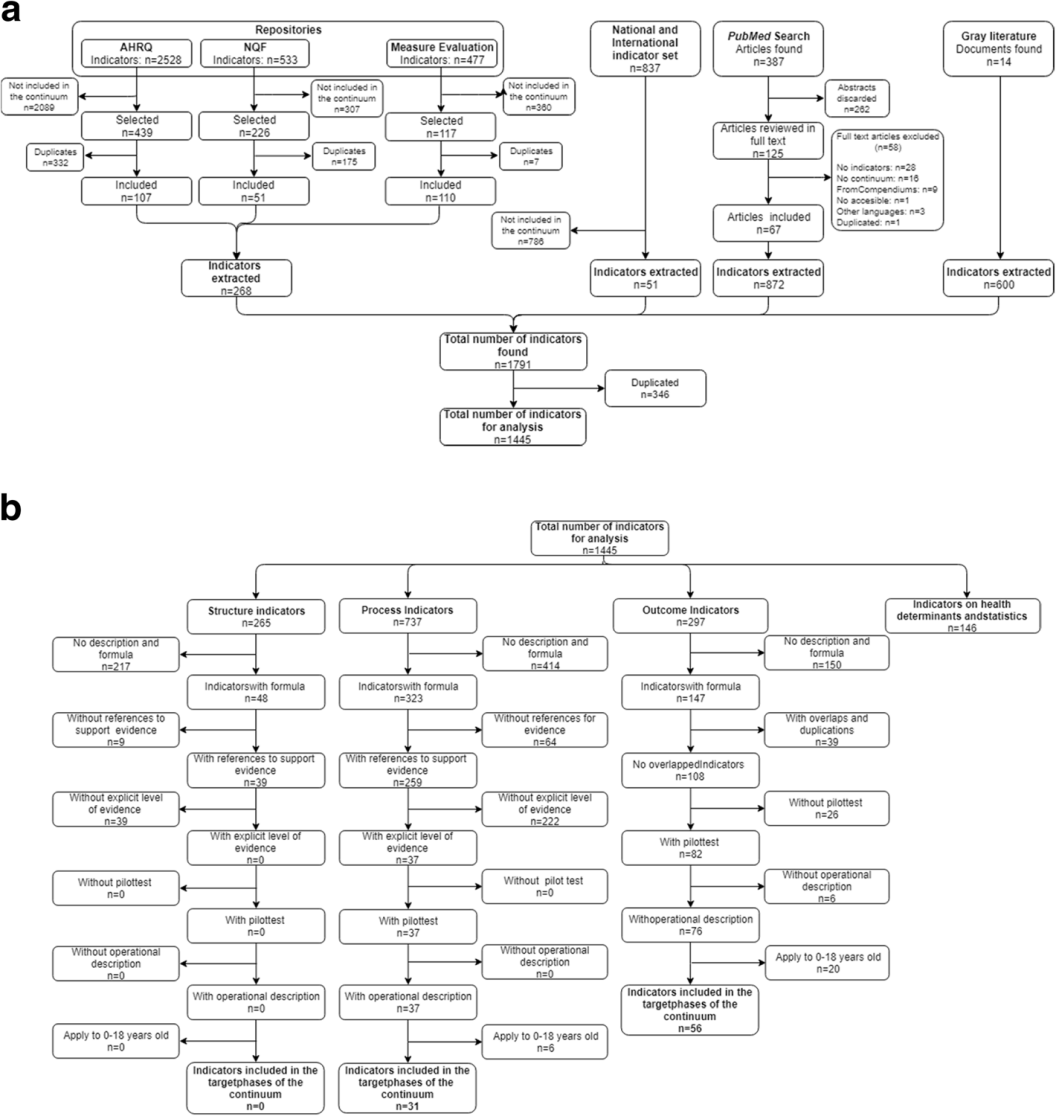
Systematic review process. **a** Systematic search of indicators. **b**. Review, selection and classification of indicators

The steps for analysis and selection are summarized in Fig. 2b. Given our objective of analyzing indicators focused on the performance of the health services, and more specifically healthcare quality, we discarded the group of indicators not directly related to healthcare (health determinants and other social and demographic statistics) for further analysis. For the remaining indicators, we analyzed the presence of the desirable characteristics associated with scientific soundness [28, 29, 32] (complete description, referenced explicit evidence, and reliability), as well as feasibility demonstrated by pilot testing [33, 34]. The strength of evidence determine validity, which is the degree to which an indicator measures what it is intended to measure, and whether the results of a measurement corresponds to the true state of the phenomenon being measured [33]. Ultimately, validity determines the likelihood that improvement in the indicator will produce consistent and credible improvements in the quality of care [33]. The reliability of a measure is also necessary for validity [34]. Therefore, to assess the validity of an indicator we look for the explicit reference to the level of evidence, and proven reliability.

Consequently, we discarded first the indicators that did not have complete formula, and then, in the case of structure and process indicators, we discarded indicators without reference to support evidence and those without explicit level of evidence. Finally, we considered whether they had been pilot tested, documenting reliability and feasibility, and had an operational description on their application, calculation and interpretation.

For outcome indicators, we discarded also those without complete formula, then those without pilot test for feasibility and reliability, and then those that did not have the full operational description for measurement (for example, when the indicator mentions “low birth weight”, the weight that must be considered “low” for the gestational age is not identified).

In cases of similar (non-contradictory) definitions for the same type of indicator, we selected the most complete or up-to-date information and, preferably, those endorsed by internationally recognized organizations (i.e., the WHO, OECD and Eurostat). When we found different definitions for the same indicator (as it was the case for instance for neonatal mortality and also for perinatal mortality), we kept both for the analysis.

Finally, indicators that apply to infants up to 18 years old were also discarded to increase specificity.

The results of the review include evidence-based, reliable, feasible and pilot-tested indicators, which are grouped according to the main classification axes (type of indicator, activities or aspects measured, and phase of the continuum that apply), as well as by the level of care (primary, hospital, or both), organization within the health system (unit or service, health facility, supra-facility, population, or system), and economic level of the country for which they are proposed.

## Results

The general outline of the study and the overall results are described in Fig. 2, depicted in a PRISMA Statement-based flow diagram [35]. From an initial selection of 1445 indicators, we identified 87 (6.7%, after discarding indicators on health determinants and statistics) that comply with the requirements of full description and empirically tested validity, reliability and feasibility.

### Identification of indicators for analysis (Fig. 2a)

We identified in repositories a total of 3538 indicators (2528 in NQMC; 533 in NQF and 477 in MEASURE Evaluation), of which 782 were related to the phases of the *continuum of care* of interest. Of these indicators, 514 were duplicated, thus resulting in 268 indicators for further analysis (Fig. 2a).

National and international indicator sets yielded 837 indicators, of which 51 indicators evaluate the care of pregnancy, childbirth, puerperium and the newborn and were kept for analysis**.**

One hundred twenty-five of the 387 articles identified in the literature search were related to the population groups and topics of interest, according to their abstracts. After reviewing the full text, 58 articles were excluded because they did not describe indicators, the indicators were not related to the target phases of the *continuum*, they used indicators from the analyzed repositories, or they were duplicated. In some cases, the full text was in a language other than English or Spanish. Eventually, we identified 872 indicators in the 67 full text articles reviewed.

We identified 14 grey literature documents, and 600 indicators were extracted for analysis.

The initial database with the indicators extracted from all sources contained 1791 indicators. Three hundred forty-six were duplicated and discarded, thus resulting in 1445 indicators for further analysis (Fig. 2a).

### Characteristics and classification of the analyzed indicators

Process indicators predominate in all phases of the continuum (Table 2). They represent 51% of the indicators and 75% of the indicators related to pregnancy. Childbirth and Pregnancy were the phases of the continuum for which more indicators were identified, i.e., 299 and 297 indicators respectively compared to 119 indicators related to the postpartum period and 277 indicators related to newborn care.

**Table 2:** Characteristics of the indicators included in the analysis by phase of the *continuum*

Type and applicability of the indicator	Phase of the continuum of maternal and child care						Total n(%)
	Pregnancy n(%)	Childbirth n(%)	Puerperium n(%)	Newborn^a^ n(%)	Child^b^ n(%)	Other^c^ n(%)	
Indicator type							
Structure	27 (9.1)	47 (15.7)	14 (11.8)	43 (15.5)	15 (7.7)	**119 (46)**	265 (18.3)
Process	**223 (75.1)**	**146 (48.8)**	**82 (68.9)**	**122 (44)**	**119 (61.3)**	45 (17.4)	**737 (51)**
Result	12 (4)	65 (21.7)	16 (13.5)	86 (31.1)	41 (21.1)	77 (29.7)	297 (20.6)
Health determinants and statistics	35 (11.8)	41 (13.7)	7 (5.9)	26 (9.4)	19 (9.8)	18 (7)	146 (10.1)
Level of care							
Primary care	**174 (58.6)**	35 (11.7)	32 (26.9)	35 (12.6)	49 (25.3)	25 (9.7)	350 (24.2)
Hospital care 2nd or 3rd level	89 (30)	**143 (47.8)**	**57 (47.9)**	**159 (57.4)**	57 (29.4)	**118 (45.6)**	**623 (43.1)**
Both	34 (11.5)	121 (40.5)	30 (25.2)	83 (30)	**88 (45.4)**	116 (44.8)	472 (32.7)
Application-level							
Care unit, department or service	18 (6.1)	32 (10.7)	20 (16.8)	50 (18.1)	26 (13.4)	3 (1.2)	149 (10.3)
Health facility	132 (44.4)	**161 (53.9)**	**72 (60.5)**	**139 (50.2)**	48 (24.7)	**174 (67.2)**	**726 (50.2)**
Population level	**147 (49.5)**	106 (35.5)	27 (22.7)	88 (31.8)	**120 (61.9)**	82 (31.7)	570 (39.5)
Income level of the country							
Middle and low income	111 (37.4)	102 (34.1)	38 (31.9)	95 (34.3)	**96 (49.5)**	91 (35.1)	**533 (36.9)**
High income	**150 (50.5)**	75 (25.1)	**47 (39.5)**	**102 (36.8)**	82 (42.3)	22 (8.5)	478 (33.1)
High, middle and low income	36 (12.1)	**122 (40.8)**	34 (28.6)	80 (28.9)	16 (8.3)	**146 (56.4)**	434 (30)
Total (%)	**297 (100)**	**299 (100)**	**119 (100)**	**277 (100)**	**194 (100)**	**259 (100)**	**1445 (100)**

^a^First 2 months of life

^b^0 to 18 years of life

^c^Indicators applying to two or more phases of the continuum

**In bold**: the largest group in each particular phase of the continuum

In relation to the level of care, 43.1% of indicators are related to hospital care. Indicators applicable to primary care represent 24.2% of the indicators found, and are mainly for pregnancy care (58.6% of the total) and, to a lesser extent, puerperium (26.9%).

In relation to the organizational level, the majority of indicators are intended for use at health facility level, particularly in the phases related to puerperium (60.5%) and childbirth (53.9%), whereas the remaining indicators are intended for use at the supra-institutional or population level level (Table 2).

In relation to the income level of the countries in which they are applied, there is a slight predominance of middle and low-income countries in general (36.9%), even though high-income countries predominate in indicators related to pregnancy, puerperium, and newborn (Table 2).

According to the object of measurement (Table 3), preventive activities represent 16.2% of the total number of indicators and are the relative majority in the Pregnancy and Newborn phases of the continuum. Indicators related to the mortality and morbidity of newborns are more frequent in the outcomes group. It is relatively remarkable the number of structure indicators related to policy and the context of the system of health (7.3%), as well as human and material resources (9.4% of the total).

**Table 3:** Indicators included in the analysis according to the phase of the continuum and the object of measurement

Measurement object	Phase of the continuum of maternal and child care						Total n(%)
	Pregnancy n(%)	Childbirth n(%)	Puerperium n(%)	Newborn^a^ n(%)	Child^b^ n(%)	Other^c^ n(%)	
Structure							
Design, policy and health system context	11 (3.7)	22 (7.4)	7 (5.9)	12 (4.3)	7 (3.6)	46 (17.8)	105 (7.3)
Human resources and infrastructure	8 (2.7)	18 (6)	7 (5.9)	30 (10.8)	5 (2.6)	68 (26.3)	136 (9.4)
Mother, child, family and community context	8 (2.7)	7 (2.3)	0 (0)	1 (0.4)	3 (1.6)	5 (1.9)	24 (1.7)
Process							
Preventive activities	70 (23.6)	42 (14.1)	21 (17.7)	58 (20.9)	26 (13.4)	17 (6.6)	234 (16.2)
Diagnostic and/or screening	73 (24.6)	16 (5.4)	5 (4.2)	16 (5.8)	9 (4.6)	2 (0.8)	121 (8.4)
Treatment activities	48 (16.2)	15 (5)	18 (15.1)	20 (7.2)	42 (21.7)	8 (3.1)	151 (10.5)
Other maternal and childcare processes	32 (10.8)	73 (24.4)	38 (31.9)	28 (10.1)	42 (21.7)	18 (7)	231 (16)
Outcome							
Maternal mortality	1 (0)	0 (0)	4 (3.4)	2 (0.7)	0 (0)	19 (7.3)	26 (1.8)
Child mortality	0 (0)	6 (2)	1 (0.8)	31 (11.2)	14 (7.2)	12 (4.6)	64 (4.4)
Maternal morbidity	8 (2.7)	13 (4.4)	7 (5.9)	0 (0)	1 (0.5)	15 (5.8)	44 (3)
Child morbidity	1 (0.3)	4 (1.3)	1 (0.8)	38 (13.7)	14 (7.2)	0 (0)	58 (4)
Safety in the care of the mother	0 (0)	13 (4.4)	2 (1.7)	1 (0.4)	0 (0)	2 (0.8)	18 (1.3)
Safety in child care	0 (0)	0 (0)	0 (0)	7 (2.5)	3 (1.6)	1 (0.4)	11 (0.8)
Satisfaction	0 (0)	2 (0.7)	0 (0)	0 (0)	8 (4.1)	18 (7)	28 (1.9)
Other health outcomes	2 (0.7)	27 (9)	1 (0.8)	7 (2.5)	1 (0.5)	10 (3.9)	48 (3.3)
Determinants and health statistics							
Knowledge and healthy lifestyle	16 (5.4)	5 (1.7)	2 (1.7)	7 (2.5)	2 (1)	1 (0.4)	33 (2.3)
Working and living conditions	2 (0.7)	2 (0.7)	0 (0)	1 (0.4)	1 (0.5)	3 (1.2)	9 (0.6)
Environmental factors	0 (0)	0 (0)	0 (0)	0 (0)	0 (0)	1 (0.4)	1 (0.1)
Demographic and other health statistics	17 (5.7)	34 (11.4)	5 (4.2)	18 (6.5)	16 (8.3)	13 (5)	103 (7.1)
Total n(%)	297 (100)	299 (100)	119 (100)	277 (100)	194 (100)	259 (100)	1445 (100)

^a^First 2 months of life

^b^0 to 18 years of life

^c^Indicators applying to two or more phases of the continuum

### Selection of valid, reliable and pilot-tested indicators

After the application of the criteria on validity, reliability, feasibility and pilot testing (Fig. 2b, Table 4), 87 of the 1445 analyzed indicators were selected. Databases with all indicators analyzed are available online.

**Table 4:** Classification of the indicators selected in the review (*n* = 87)

Measurement object	*Continuum of care*					*Level of care*			*Application-level*			*Country’s income*			Total
	Pregnancy	Childbirth	Puerperium	Newborn^a^	Other^b^	Primary care	Hospital care	Both	Care unit	Health facility	Supra-institutional	Middle and low	High	All	
Process (31, 35.6%)															
Preventive activities	2	6	1	2	1	4	8	–	–	9	3	–	8	4	12
Diagnostic and/or screening	–	1	–	1	–	–	2	–	–	2	–	–	2	–	2
Treatment activities	5	3	–	–	–	–	8	–	–	8	–	–	–	8	8
Other maternal and childcare processes	–	7	–	1	1	–	8	1	–	9	–	–	4	5	9
Outcome (56, 64.4%)															
Maternal mortality	–	–	–	–	6	–	–	6	–	4	2	5	–	1	6
Child mortality	–	1	–	6	8	–	2	13	1	1	13	–	5	10	15
Maternal morbidity	1	4	1	–	6	1	3	8	1	7	4	5	2	5	12
Child morbidity	–	–	–	12	–	–	9	3	7	1	4	2	9	1	12
Safety in the care of the mother	–	2	–	–	–	–	2	–	1	–	1	–	1	1	2
Safety in child care	–	–	–	2	–	–	2	–	1	1	–	–	2	–	2
Other health outcomes	–	5	–	2	–	1	5	1	4	–	3	1	4	2	7
Total (%)	8	29	2	26	22	6	49	32	15	42	30	13	37	37	87
*% of indicators reviewed*	*2.7*	*9.7*	*1.7*	*9.4*	*8.5*	*1.7*	*7.9*	*6.8*	*10.1*	*5.8*	*5.3*	*2.4*	*7.7*	*8.5*	*6.0*

^a^First 2 months of life

^b^Indicators applying to more than two phases of the continuum

We initially discarded 146 indicators not directly related to health care (social determinants and demographic statistics). More than half (781, 60%) of the remaining 1299 indicators did not report their formula, which made their reproducibility impossible. However, the differences between the indicators extracted from different sources are noteworthy: all indicators from repositories and 89.1% of indicators extracted from systems or official indicator sets describe their formulas compared with less than half of the indicators identified from the grey literature and only 32.3% of the indicators obtained from scientific publications.

In the groups of structure and process indicators with formulas, 73 (19.7%) indicators were discarded because there was no reference to support their evidence, and 261 (an additional 70.4%) indicators were discarded because the level of evidence was not explicitly stated. Referenced and explicit evidence are the first and key criteria for validity.

In the outcome group, 39 (26.5%) of the 147 indicators with formula were duplicated or overlapped, and 26 (24.1%) of the remaining indicators were discarded because of the absence of a pilot study to demonstrate feasibility and reliability, or clear operational description of their application, calculation and interpretation.

Finally, to increase specificity we discarded 26 indicators that applied to an age range of 0 to 18 years. The final selection includes 31 process indicators and 56 outcome indicators (Fig. 2b, Table 4).

A more detailed analysis of the selected indicators, in view of their potential use for monitoring the quality of maternal and perinatal care, indicates certain imbalances and gaps (Table 4). Thus, they do not cover all possible categories according to the type of indicator and the object of measurement in any of the considered phases of the continuum. Most indicators relate to childbirth or newborns; however, very few relate to puerperium. The majority are applicable at the hospital level, and only a limited number exclusively relate to primary care. In addition, the majority are proposed for assessing quality at the facility level and for high-income countries, with a limited number for middle and low-income environments only.

The low proportion of indicators selected (6.7% of the analyzed indicators, after discarding indicators on health determinants and statistics) reflects a low level of scientific soundness and proven feasibility of the published indicators. This proportion is, nevertheless, uneven according to the phase of the continuum and the level of application within the health system. Calculating these proportions using data from Table 4 and Table 2, they are somehow higher for the indicators that apply to childbirth (9.7%), hospital care (8.7%), and department or service unit (12.2%). Indicators related to pregnancy are one of the largest group of extracted and analyzed indicators (Table 2); however, they are also one of the lowest percentages of indicators that comply with the criteria used in the review (2.7%). Puerperium, primary healthcare, and supra-institutional or system level indicators yield the lowest percentage of scientifically sound indicators. In particular, for primary health care, only 6 of the 311 analyzed (1.9%) indicators were selected.

Additional file 3 provides the description and reference documents of the 87 selected indicators in English and Spanish, ordered by the phase of the continuum.

## Discussion

This review provides an abundance of initiatives, frameworks and indicators to monitor the quality of pregnancy, childbirth, postpartum and newborn care. However, in terms of proven scientific soundness, the quality is largely poor. Moreover, indicators that meet the requirements do not cover all aspects expected and only relate to parts of the continuum, levels of care, such as primary care, and levels of application within the system, such as the supra-institutional, where there is a dearth of indicators with the necessary guarantees to consider their adaptation and routine use.

### Abundance of indicators, but limited scientific rigor

Doctors, administrators, policy makers and patients require reliable and valid information to perform comparative evaluations, make judgments, determine priorities and improve the quality of care [33]. Therefore, there is a need for indicators that are easy to interpret, reasonable, validated and adapted to the characteristics of the context of each country [8]. In the pursuit of the achievement of the MDG, as well as the SDG, the need to assess the quality of care has been strongly acknowledged [8, 9, 11, 36]. This has motivated and given momentum to the development and proposal of indicators [8, 9, 37–40]. However, according to our review, more than half of the indicators are not properly reported, and the vast majority of the remaining indicators lack the necessary methodological rigor. Another review, that included only indicators proposed by global multi-stakeholder groups, found that 25% of all indicators is either under development or requires a clear definition and methodology [40]. A common feature is the use of consensus techniques to select indicators, which is to the detriment of proven and explicit criteria to ensure their scientific robustness. These criteria are often described as desirable and a guide for the selection; however, this selection is eventually left to the opinion of the consulted experts. Consensus may be important as a final step to guarantee acceptance; however, we suggest that the initial selection should be made on solid scientific grounds. We have determined that this is not the most frequent case.

Three other issues of relevance for the creation of monitoring systems for MCH are worth highlighting because they are not clearly addressed: 1) the desirable integration of the indicators in a coordinated set which takes into account the different levels of responsibility within the health system [41]; 2) the limited attention paid to the importance of the quality of data; and 3) the scarcity of indicators based on data reported by patients. We have shown, as indicated in other publications [9, 12], that the level of detail on the processes addressed for monitoring is often not suitable for routine information systems. Most valid indicators are intended for use at the micro level (service unit or facility) (Table 4), whereas other indicators intended for the system level ignore lower levels in the organization. Metrics may need to vary at different levels of the healthcare system; however, all sets should be aligned. This is not visible in the current situation. In addition, some indicators seem to address coverage of services (i.e. number of antenatal services) rather than direct quality of care (i.e. the right contents of antenatal care or the right clinical decisions for particular MCH conditions).

The quality of the data is not directly addressed in our review. However, the lack of explicit reference to the data required for the calculation of the indicators (the first criterion for discarding them; refer to Fig. 2b), as well as the indicators proposed without pilot tests (particularly among outcome indicators), may be considered as proxies for the limited attention paid to the quality of data required for implementing the indicators.

Patient-reported indicators are considered by international organizations, such as the OECD, as the next generation of health statistics [42], and survey-based indicators on patient experience have a salient place in the current monitoring systems of quality of care in general. However, they are barely present in the final selection in our review. We found 28 indicators measuring satisfaction (see Table 3), but most of them were complex, including more than one phase of the continuum, and they did not have the required criteria for being included in the final selection.

It is striking that indicators identified from the scientific literature are of poorer quality in terms of a lower compliance with the selection criteria established than the extracted indicators from repositories or established systems of measurement (Fig. 2b). This issue primarily arises because authors fail to accurately describe how to measure them, as well as the reduced attention given to explicitly state the level of evidence that supports them. Specialized repositories (NQMC and NQF) are good sources for well-described indicators, as well as indicators developed by agencies, such as the WHO [9, 10], USAID [43] and other agencies [44]. However, we have not identified an extensive and systematic review as the one presented here.

### A comprehensive set with significant gaps

As indicated in the total column in Table 4, there are indicators for all considered aspects of the continuum, as well as all levels of attention and levels of responsibility within the health system. However, a complete and coordinated set must be constructed. Incomplete proposals, which referred to a single phase of the continuum or very specific aspects within the same phase, are frequent. The distribution of the indicators by groups is also very uneven. As a result, although the selection we present forms the basis, or first step, to determine and adapt a relatively comprehensive indicator set, there are many significant gaps. There is a persistent need to provide additional efforts to build a good indicator set for MCH care and monitor progress in the SDG [45]. The lack of indicators related to the postnatal period for the mother and, relatively, pregnancy within the continuum is particularly noticeable, which is likely associated with the shortage of fully validated indicators applicable to primary health care and the population level. The apparent abundance of indicators hides the likely need of further progress in the construction and validation of empirically tested and scientifically solid indicators to build a comprehensive and hierarchical system to monitor the quality of MHC care, in all phases of the continuum and at all levels of the health system. Recent initiatives such as the WHO Quality of Care Network for Maternal, newborn, child and adolescent health [46] may contribute to this endeavor.

The search was limited to sources in Spanish and English. There may be repositories and publications in other languages. Moreover, it is possible to search other databases, such as Embase, and attempt to enlarge the grey literature search using the snowball technique. However, we believe that most references for indicators are included in our search and that our results provide a fair view of the current situation.

## Conclusions

There is a broad panorama of indicators available internationally for the evaluation of the quality of maternal and newborn care; however, a critical analysis shows that most indicators are not readily suitable for adaptation and implementation. Only a minority of published indicators comply with the requirements of scientific validity, usefulness and feasibility empirically tested.

The indicators identified and selected in our search may comprise a good starting point; however, it is likely that they should be supplemented by new indicators to cover the needs of a comprehensive monitoring system. Our study indicates the specific aspects and levels of care and responsibility for which there is a likely need to make additional efforts in the construction and validation of quality indicators to monitor the continuum of maternal and newborn health.

## Additional files

Additional file 1: PubMed search strategy. Search strategy in PubMed. (DOCX 14 kb)

Additional file 2: References all sources. Complete list of references used in the research by source: repositories, compendiums, scientific articles (abstract discarded, full text articles excluded, and full text articles included) and grey literature. (DOCX 82 kb)

Additional file 3: Description 87 indicators. 87 indicators selected in English and Spanish ordered by each phase of the continuum with reference. (DOCX 112 kb)

## Abbreviations

MCH: Maternal and child health
MDG: Millennium Development Goals
NQF: *National Quality Forum*
NQMC: *National Quality Measures Clearinghouse*
OECD: Organization for the Economic Cooperation and Development
SDG: Sustainable Development Goals
UNICEF: United Nations International Children’s Emergency Fund
WHO: World Health Organization
