# Supplementary material for: Indicators for monitoring maternal and neonatal quality care: a systematic review
Source: BMC Pregnancy Childbirth. 2019 Jan 11;19:25. doi: 10.1186/s12884-019-2173-2 (PMC6330388; doi:10.1186/s12884-019-2173-2)
Supplement: Supplementary file 1 — PubMed search strategy. Search strategy in PubMed. (DOCX 14 kb) [file 12884_2019_2173_MOESM1_ESM.docx]

**Appendix S1.** PubMed search strategy

| **Search** | **(#1 OR #2) AND #3 AND (((#4 OR #5) AND (#6 OR #7)) OR (#8 OR #9) OR (#10 OR #11 OR #12) OR (#13 OR #14) OR (#15 OR #16))** |
| --- | --- |
| #1 | systematic[sb] OR Review[ptyp] |
| #2 | set[Title/Abstract] OR group[Title/Abstract] OR kit[Title/Abstract] OR assortment[Title/Abstract] OR collection[Title/Abstract] OR inventory[Title/Abstract] OR repository[Title/Abstract] OR battery[Title/Abstract] |
| #3 | "Quality Indicators, Health Care"[Mesh] OR "quality indicators"[All Fields] OR ("quality"[All Fields] AND "indicators"[All Fields]) OR "quality measures"[All Fields] |
| #4 | "Mothers"[Mesh] OR "Pregnant Women"[Mesh] |
| #5 | "Infant, Newborn"[Mesh] OR "Premature Birth"[Mesh] OR "Infant, Premature"[Mesh] OR "Term Birth"[Mesh] OR "Fetus"[Mesh] |
| #6 | "Pregnancy Complications"[Mesh] |
| #7 | "Infant, Newborn, Diseases"[Mesh] |
| #8 | "Maternal Health"[Mesh] OR ("maternal"[All Fields] AND "health"[All Fields]) OR "maternal health"[All Fields] |
| #9 | "Infant Health"[Mesh] OR ("infant"[All Fields] AND "health"[All Fields]) OR "infant health"[All Fields] |
| #10 | "Pregnancy"[Mesh] OR "Prenatal Care"[Mesh] |
| #11 | "Parturition"[Mesh] OR "Live Birth"[Mesh] OR "Natural Childbirth"[Mesh] OR "Home Childbirth"[Mesh] OR "Delivery, Obstetric"[Mesh] OR "Labor, Obstetric"[Mesh] OR "Peripartum Period"[Mesh] OR "Perinatal Care"[Mesh] |
| #12 | "Postpartum Period"[Mesh] OR "Postnatal Care"[Mesh] |
| #13 | ("mothers"[MeSH Terms] OR "mothers"[All Fields] OR "maternal"[All Fields]) AND care[All Fields] |
| #14 | "Infant Care"[Mesh] OR "Child Care"[Mesh] |
| #15 | "Maternal-Child Health Centers"[Mesh] OR "Maternal Health Services"[Mesh] OR "Maternal-Child Health Services"[Mesh] OR "Maternal-Child Nursing"[Mesh] OR "Obstetric Nursing"[Mesh] |
| #16 | "Intensive Care, Neonatal"[Mesh] OR "Intensive Care Units, Neonatal"[Mesh] OR "Neonatal Nursing"[Mesh] |

Search limited to five years (from 2012 to 2016).
