# Supplementary material for: Indicators for monitoring maternal and neonatal quality care: a systematic review
Source: BMC Pregnancy Childbirth. 2019 Jan 11;19:25. doi: 10.1186/s12884-019-2173-2 (PMC6330388; doi:10.1186/s12884-019-2173-2)
Supplement: Supplementary file 2 — References all sources. Complete list of references used in the research by source: repositories, compendiums, scientific articles (abstract discarded, full text articles excluded, and full text articles included) and grey literature. (DOCX 82 kb) [file 12884_2019_2173_MOESM2_ESM.docx]

**Additional file 2.** List of references used in the research by source (repositories, compendiums, scientific articles and gray literature)

| **International repositories of indicators** |
| --- |
| 1. Agency for Healthcare Research and Quality (AHRQ). National Quality Measures Clearinghouse. https://www.qualitymeasures.ahrq.gov/. Accessed 15 May 2016.  2. National Quality Forum. Find Measures. [http://www.qualityforum.org/QPS/QPSTool.aspx. Accessed 20 May 2016](http://www.qualityforum.org/QPS/QPSTool.aspx.%20Accessed%2020%20May%202016).  3. MEASURE Evaluation. Family Planning and Reproductive Health Indicators Database. Summary List of Indicators. https://www.measureevaluation.org/prh/rh_indicators/indicator-summary. Accessed 26 Jul 2018. |
| **Compendiums or established national and international indicators sets** |
| 1. The Joint Commission. Performance Measurement. Specifications Manual for National Hospital Inpatient Quality Measures. 2016. https://www.jointcommission.org/performance_measurement.aspx. Accessed 26 Jun 2016.  2. The Joint Commission. Performance Measurement. Specifications Manual for Joint Commission National Quality Core Measures. 2016. https://www.jointcommission.org/performance_measurement.aspx. Accessed 30 Nov 2016.  3. Centers for Medicare & Medicaid Services. Core Measure. 2016. https://www.cms.gov/Medicare/Quality-Initiatives-Patient-Assessment-Instruments/QualityMeasures/Core-Measures.html. Accessed 3 Jun 2016.  4. National Institute for Health and Care Excellence (NICE). Standards and Indicators. Single menu of indicators. 2016. https://www.nice.org.uk/standards-and-indicators. Accessed 15 Jun 2016.  5. NHS Digital. Clinical Commissioning Group Outcomes Indicator Set (CCG OIS). https://indicators.hscic.gov.uk/webview/. Accessed 17 Jun 2016.  6. Ministerio de Sanidad Servicios Sociales e Igualdad (MSSSI). Indicadores Clave Sistema Nacional de Salud - Versión 1.0. 2016. http://inclasns.msssi.es/main.html. Accessed 10 Jun 2016.  7. Secretaría de Salud. Dirección General de Calidad y Educación en Salud. Sistema Nacional de Indicadores de Calidad en Salud. 2016. http://dgces.salud.gob.mx/INDICASII/. Accessed 2 Jun 2016.  8. European Commission. Indicators. European Core Health Indicators (ECHI). 2016. https://ec.europa.eu/health/indicators_data/echi_en. Accessed 20 Jun 2016.  9. Organisation for Economic Co-operation and Development. OECD Health Statistics 2015. List of indicators. 2016. http://www.oecd.org/els/health-systems/health-data.htm. Accessed 24 Jun 2016. |
| **Scientific articles** |
| **Abstract discarded** |
|  |
| 1. Aghajafari F, Nagulesapillai T, Ronksley PE, Tough SC, O’Beirne M, Rabi DM. Association between maternal serum 25-hydroxyvitamin D level and pregnancy and neonatal outcomes: systematic review and meta-analysis of observational studies. BMJ. 2013;346:f1169. |
| 2. Aharonson-Daniel L, Schwartz D, Hornik-Lurie T, Halpern P. Quality of coding diagnoses in emergency departments: effects on mapping the public’s health. Isr Med Assoc J. 2014 Jan;16(1):11–6. |
| 3. AIRTUM Working Group, CCM, AIEOP Working Group. Italian cancer figures, report 2012: Cancer in children and adolescents. Epidemiol Prev. 2013;37 Suppl 1:1–225. |
| 4. Al-Akour NA, Khader YS, Khassawneh MY, Bawadi H. Health-related quality of life of adolescents with overweight or obesity in the north of Jordan. Child Care Health Dev. 2012;38(2):237–43. |
| 5. American College of Gynecologists and Obstetricians. The importance of vital records and statistics for the obstetrician–gynecologist. Committee Opinion Summary No. 639. Obstet Gynecol. 2015;126(3):690. |
| 6. American Diabetes Association. Executive summary: Standards of medical care in diabetes--2012. Diabetes Care. 2012;35 Suppl 1:S4–10. |
| 7. Andersson E, Christensson K, Hildingsson I. Mothers’ satisfaction with group antenatal care versus individual antenatal care--a clinical trial. Sex Reprod Healthc. 2013;4(3):113–20. |
| 8. Antier C, Kumar S, Bhagwat S, Sankar R. Production of fortified food for a public supplementary nutrition program: performance and viability of a decentralised production model for the Integrated Child Development Services Program, India. Asia Pac J Clin Nutr. 2014;23 Suppl 1:S20-8. |
| 9. Arrowsmith SD, Barone MA, Ruminjo J. Outcomes in obstetric fistula care: a literature review. Curr Opin Obstet Gynecol. 2013;25(5):399–403. |
| 10. Azria E, Guittet L, Delahaye M, Koskas M, Naoura I, Luton D, et al. Improvement of first-trimester ultrasound screening in socially deprived settings through a community-based perinatal network. Eur J Obstet Gynecol Reprod Biol. 2011;159(2):351–4. |
| 11. Baghirzada L, Downey KN, Macarthur AJ. Assessment of quality of life indicators in the postpartum period. Int J Obstet Anesth. 2013;22(3):209–16. |
| 12. Balaban B, Sakkas D, Gardner DK. Laboratory procedures for human in vitro fertilization. Semin Reprod Med. 2014;32(4):272–82. |
| 13. Bardach NS, Vittinghoff E, Asteria-Peñaloza R, Edwards JD, Yazdany J, Lee HC, et al. Measuring hospital quality using pediatric readmission and revisit rates. Pediatrics. 2013;132(3):429–36. |
| 14. Barrasa Villar JI, Castán Ruiz S, Estupiñán Romero FR, Valderrama Rodríguez M, Moliner Lahoz FJ. [Amenable mortality as indicator of effectiveness of health care services in Spain before and after transferences]. Rev Calid Asist  organo la Soc Española Calid Asist. 2013;28(2):84–95. |
| 15. Basra MKA, Gada V, Ungaro S, Finlay AY, Salek SM. Infants’ Dermatitis Quality of Life Index: a decade of experience of validation and clinical application. Br J Dermatol. 2013;169(4):760–8. |
| 16. Battini M, Vieira A, Barbieri S, Ajuda I, Stilwell G, Mattiello S. Invited review: Animal-based indicators for on-farm welfare assessment for dairy goats. J Dairy Sci. 2014;97(11):6625–48. |
| 17. Been J V, Lievense S, Zimmermann LJI, Kramer BW, Wolfs TGAM. Chorioamnionitis as a risk factor for necrotizing enterocolitis: a systematic review and meta-analysis. J Pediatr. 2013;162(2):236–42.e2. |
| 18. Beigi M, Javanmardi Z, Khani B, Safdari F. The effect of using maternal care log book on pregnancy outcome in clients referred to private gynecologists and midwives offices. Iran J Nurs Midwifery Res. 2011;16(3):222–6. |
| 19. Belaid L, Dumont A, Chaillet N, De Brouwere V, Zertal A, Hounton S, et al. Protocol for a systematic review on the effect of demand generation interventions on uptake and use of modern contraceptives in LMIC. Syst Rev. 2015;4:124. |
| 20. Bell JC, Raynes-Greenow C, Turner RM, Bower C, Nassar N, O’Leary CM. Maternal alcohol consumption during pregnancy and the risk of orofacial clefts in infants: a systematic review and meta-analysis. Paediatr Perinat Epidemiol. 2014;28(4):322–32. |
| 21. Bendavid E. Changes in child mortality over time across the wealth gradient in less-developed countries. Pediatrics. 2014;134(6):e1551-9. |
| 22. Benz JK, Espinosa O, Welsh V, Fontes A. Awareness of racial and ethnic health disparities has improved only modestly over a decade. Health Aff (Millwood). 2011;30(10):1860–7. |
| 23. Berghella V, Baxter JK, Hendrix NW. Cervical assessment by ultrasound for preventing preterm delivery. Cochrane database Syst Rev. 2013;1:CD007235. |
| 24. Berry JG, Toomey SL, Zaslavsky AM, Jha AK, Nakamura MM, Klein DJ, et al. Pediatric readmission prevalence and variability across hospitals. JAMA. 2013;309(4):372–80. |
| 25. Beusterien KM, Yeung J-E, Pang F, Brazier J. Development of the multi-attribute Adolescent Health Utility Measure (AHUM). Health Qual Life Outcomes. 2012;10:102. |
| 26. Bhatt M, Roback MG, Joubert G, Farion KJ, Ali S, Beno S, et al. The design of a multicentre Canadian surveillance study of sedation safety in the paediatric emergency department. BMJ Open. 2015;5(5):e008223. |
| 27. Bibbo C, Robinson JN. Management of twins: vaginal or cesarean delivery? Clin Obstet Gynecol. 2015 Jun;58(2):294–308. |
| 28. Bird SR, Noronha M, Kurowski W, Orkin C, Sinnott H. Integrated care facilitation model reduces use of hospital resources by patients with pediatric asthma. J Healthc Qual. 2012;34(3):25–33. |
| 29. Black A, Guilbert E, Costescu D, Dunn S, Fisher W, Kives S, et al. Canadian Contraception Consensus (Part 2 of 4). J Obstet Gynaecol Can. 2015;37(11):1033–9. |
| 30. Blaney S, Februhartanty J, Sukotjo S. Feeding practices among Indonesian children above six months of age: a literature review on their magnitude and quality (part 1). Asia Pac J Clin Nutr. 2015;24(1):16–27. |
| 31. Bond DM, Gordon A, Hyett J, de Vries B, Carberry AE, Morris J. Planned early delivery versus expectant management of the term suspected compromised baby for improving outcomes. Bond DM, editor. Cochrane database Syst Rev. 2015;11:CD009433. |
| 32. Bonney PA, Glenn CA, Ebeling PA, Conner AK, Boettcher LB, Cameron DM, et al. Seizure Freedom Rates and Prognostic Indicators After Resection of Gangliogliomas: A Review. World Neurosurg. 2015;84(6):1988–96. |
| 33. Bosse G, Ngoli B, Leshabari MT, Külker R, Dämmrich T, Abels W, et al. Quality of health care and the need for assessment. East Afr J Public Health. 2011;8(3):199–204. |
| 34. Bozic B, Bajcetic M. Use of antibiotics in paediatric primary care settings in Serbia. Arch Dis Child. 2015;100(10):966–9. |
| 35. Bozicevic I, Riedner G, Calleja JMG. HIV surveillance in MENA: recent developments and results. Sex Transm Infect. 2013;89 Suppl 3:iii11-16. |
| 36. Bradley E, Thompson JW, Byam P, Webster TR, Zerihun A, Alpern R, et al. Access and quality of rural healthcare: Ethiopian Millennium Rural Initiative. Int J Qual Health Care. 2011;23(3):222–30. |
| 37. Brewster D. Science and ethics of human immunodeficiency virus/acquired immunodeficiency syndrome controversies in Africa. J Paediatr Child Health. 2011;47(9):646–55. |
| 38. Briere EC, Ryman TK, Cartwright E, Russo ET, Wannemuehler KA, Nygren BL, et al. Impact of integration of hygiene kit distribution with routine immunizations on infant vaccine coverage and water treatment and handwashing practices of Kenyan mothers. J Infect Dis. 2012;205 Suppl:S56-64. |
| 39. Britton JR. The assessment of satisfaction with care in the perinatal period. J Psychosom Obstet Gynaecol. 2012;33(2):37–44. |
| 40. Brown MJ, Sinclair M, Liddle D, Hill AJ, Madden E, Stockdale J. A systematic review investigating healthy lifestyle interventions incorporating goal setting strategies for preventing excess gestational weight gain. PLoS One. 2012;7(7):e39503. |
| 41. Bruny JL, Hall BL, Barnhart DC, Billmire DF, Dias MS, Dillon PW, et al. American College of Surgeons National Surgical Quality Improvement Program Pediatric: a beta phase report. J Pediatr Surg. 2013;48(1):74–80. |
| 42. Brussoni M, Gibbons R, Gray C, Ishikawa T, Sandseter EBH, Bienenstock A, et al. What is the Relationship between Risky Outdoor Play and Health in Children? A Systematic Review. Int J Environ Res Public Health. 2015;12(6):6423–54. |
| 43. Bryant M, Ashton L, Brown J, Jebb S, Wright J, Roberts K, et al. Systematic review to identify and appraise outcome measures used to evaluate childhood obesity treatment interventions (CoOR): evidence of purpose, application, validity, reliability and sensitivity. Health Technol Assess. 2014;18(51):1–380. |
| 44. Buchner F, Goepffarth D, Wasem J. The new risk adjustment formula in Germany: implementation and first experiences. Health Policy. 2013;109(3):253–62. |
| 45. Bundy DG, Solomon BS, Kim JM, Miller MR. Accuracy and usefulness of the HEDIS childhood immunization measures. Pediatrics. 2012;129(4):648–56. |
| *****46. Cahill AG, Spain J. Intrapartum fetal monitoring. Clin Obstet Gynecol. 2015;58(2):263–8. |
| 47. Campbell H, El Arifeen S, Hazir T, O’Kelly J, Bryce J, Rudan I, et al. Measuring coverage in MNCH: challenges in monitoring the proportion of young children with pneumonia who receive antibiotic treatment. PLoS Med. 2013;10(5):e1001421. |
| 48. Cantero M, Redondo M, Martin E, Callejon G, Hortas ML. Use of quality indicators to compare point-of-care testing errors in a neonatal unit and errors in a STAT central laboratory. Clin Chem Lab Med. 2015;53(2):239–47. |
| 49. Caughey AB, Sharshiner R, Cheng YW. Fetal malposition: impact and management. Clin Obstet Gynecol. 2015;58(2):241–5. |
| 50. Chang E. The role of simulation training in obstetrics: a healthcare training strategy dedicated to performance improvement. Curr Opin Obstet Gynecol. 2013;25(6):482–6. |
| 51. Chang H-Y. The impact of morbidity trajectories on identifying high-cost cases: using Taiwan’s National Health Insurance as an example. J Public Health (Oxf). 2014;36(2):300–7. |
| 52. Chavane L, Merialdi M, Betrán AP, Requejo-Harris J, Bergel E, Aleman A, et al. Implementation of evidence-based antenatal care in Mozambique: a cluster randomized controlled trial: study protocol. BMC Health Serv Res. 2014;14:228. |
| 53. Chaves AH, Sebastian J, Hoopes S, Rosenthal GL. The Effect of a Quality Improvement Intervention on Variability of Measurements of Left Ventricular Dimensions in a Pediatric Echocardiography Laboratory. Congenit Heart Dis. 2015;10(4):340–5. |
| 54. Cheng YW, Caughey AB. Second stage of labor. Clin Obstet Gynecol. 2015;58(2):227–40. |
| 55. Cheung CRLH, Lemer C. Using data to improve care. Arch Dis Child Educ Pract Ed. 2013;98(6):224–9. |
| 56. Cheung R, Ardolino A, Lawrence T, Bouamra O, Lecky F, Berry K, et al. The accuracy of existing prehospital triage tools for injured children in England--an analysis using trauma registry data. Emerg Med J. 2013;30(6):476–9. |
| 57. Chien AT, Song Z, Chernew ME, Landon BE, McNeil BJ, Safran DG, et al. Two-year impact of the alternative quality contract on pediatric health care quality and spending. Pediatrics. 2014;133(1):96–104. |
| 58. Christian P, Lee SE, Donahue Angel M, Adair LS, Arifeen SE, Ashorn P, et al. Risk of childhood undernutrition related to small-for-gestational age and preterm birth in low- and middle-income countries. Int J Epidemiol. 2013;42(5):1340–55. |
| 59. Clarke DL, Kong VY, Handley J, Aldous C. A concept paper: using the outcomes of common surgical conditions as quality metrics to benchmark district surgical services in South Africa as part of a systematic quality improvement programme. S Afr J Surg. 2013;51(3):84–6. |
| 60. Cohen E, Mahant S, Dell SD, Traubici J, Ragone A, Wadhwa A, et al. The long-term outcomes of pediatric pleural empyema: a prospective study. Arch Pediatr Adolesc Med. 2012;166(11):999–1004. |
| 61. Coller RJ, Klitzner TS, Lerner CF, Chung PJ. Predictors of 30-day readmission and association with primary care follow-up plans. J Pediatr. 2013;163(4):1027–33. |
| 62. Courville EL, Lew M, Sadow PM. Routine evaluation of adult tonsillectomy specimens: toward establishing a new standard of care. Int J Surg Pathol. 2011;19(4):469–75. |
| 63. Crowe S, Brown KL, Pagel C, Muthialu N, Cunningham D, Gibbs J, et al. Development of a diagnosis- and procedure-based risk model for 30-day outcome after pediatric cardiac surgery. J Thorac Cardiovasc Surg. 2013;145(5):1270–8. |
| 64. Csont GL, Groth S, Hopkins P, Guillet R. An evidence-based approach to breastfeeding neonates at risk for hypoglycemia. J Obstet Gynecol Neonatal Nurs. 2014;43(1):71–81. |
| 65. Dayal A, Alvarez F. The Effect of Implementation of Standardized, Evidence-Based Order Sets on Efficiency and Quality Measures for Pediatric Respiratory Illnesses in a Community Hospital. Hosp Pediatr. 2015;5(12):624–9. |
| 66. de Jong M, Lafeber HN, Cranendonk A, van Weissenbruch MM. Components of the metabolic syndrome in early childhood in very-low-birth-weight infants. Horm Res pædiatrics. 2014;81(1):43–9. |
| 67. den Breejen EME, Nelen WLDM, Schol SFE, Kremer JAM, Hermens RPMG. Development of guideline-based indicators for patient-centredness in fertility care: what patients add. Hum Reprod. 2013;28(4):987–96. |
| 68. Desouky TF, Mora PA, Howell EA. Measurement invariance of the SF-12 across European-American, Latina, and African-American postpartum women. Qual Life Res. 2013;22(5):1135–44. |
| 69. Dilber D, Malcic I, Dasovic Buljevic A, Anic D, Belina D, Zovko A. Croatian clinical epidemiological study (2008-2011): the use of standardised risk scores in paediatric congenital cardiac surgery for a case complexity selection and gradual progress of cardiosurgical model in developing countries. Cardiol Young. 2015;25(2):274–80. |
| 70. Doherty S, Knott J, Bennetts S, Jazayeri M, Huckson S. National project seeking to improve pain management in the emergency department setting: findings from the NHMRC-NICS National Pain Management Initiative. Emerg Med Australas. 2013;25(2):120–6. |
| 71. Dolgun E, Yavuz M, Celik A, Ergün MO. The effects of constipation on the quality of life of children and mothers. Turk J Pediatr. 2013;55(2):180–5. |
| 72. Dotta A, Portanova A, Bianchi N, Ciofi Degli Atti M, Zanini R, Raponi M. Accreditation of birth centres: advantages for newborns. J Matern Fetal Neonatal Med. 2013;26(4):417–8. |
| 73. d’Udekem Y, Galati JC, Konstantinov IE, Cheung MH, Brizard CP. Intersurgeon variability in long-term outcomes after transatrial repair of tetralogy of Fallot: 25 years’ experience with 675 patients. J Thorac Cardiovasc Surg. 2014;147(3):880–6. |
| 74. Duggan EM, Patel VP, Blakely ML. Inguinal hernia repair in premature infants: more questions than answers. Arch Dis Child Fetal Neonatal Ed. 2015;100(4):F286-8. |
| 75. Edwards SE, Grobman WA, Lappen JR, Winter C, Fox R, Lenguerrand E, et al. Modified obstetric early warning scoring systems (MOEWS): validating the diagnostic performance for severe sepsis in women with chorioamnionitis. Am J Obstet Gynecol. 2015;212(4):536.e1-8. |
| 76. Ehrenberg M, Nihalani BR, Melvin P, Cain CE, Hunter DG, Dagi LR. Goal-determined metrics to assess outcomes of esotropia surgery. J AAPOS. 2014;18(3):211–6. |
| 77. Eisenberg DF, Gu T, Krishnarajah G. Adherence to rotavirus vaccination quality measures in a commercially insured population. Hum Vaccin Immunother. 2013;9(2):389–97. |
| 78. Ekart J, McNatty K, Hutton J, Pitman J. Ranking and selection of MII oocytes in human ICSI cycles using gene expression levels from associated cumulus cells. Hum Reprod. 2013;28(11):2930–42. |
| 79. El Haj Ibrahim S, Fridman M, Korst LM, Gregory KD. Anesthesia complications as a childbirth patient safety indicator. Anesth Analg. 2014;119(4):911–7. |
| 80. Fingar KR, Barrett ML, Elixhauser A, Stocks C, Steiner CA. Trends in Potentially Preventable Inpatient Hospital Admissions and Emergency Department Visits: Statistical Brief #195. Healthcare Cost and Utilization Project (HCUP) Statistical Briefs. Rockville (MD); 2015. |
| 81. Flamand VH, Masse-Alarie H, Schneider C. Psychometric evidence of spasticity measurement tools in cerebral palsy children  and adolescents: a systematic review. J Rehabil Med. 2013;45(1):14–23. |
| 82. Ford ND, Stein AD. Risk factors affecting child cognitive development: a summary of nutrition, environment, and maternal-child interaction indicators for sub-Saharan Africa. J Dev Orig Health Dis. 2016;7(2):197–217. |
| 83. Fulton Z, Briggs D, Silva S, Szalay EA. Calf circumference discrepancies in patients with unilateral clubfoot: Ponseti versus surgical release. J Pediatr Orthop. 2015;35(4):403–6. |
| 84. Gagnon AJ, Carnevale F, Mehta P, Rousseau H, Stewart DE. Developing population interventions with migrant women for maternal-child health: a focused ethnography. BMC Public Health. 2013;13:471. |
| 85. Gesundheit B, Rosenzweig JP, Naor D, Lerer B, Zachor DA, Procházka V, et al. Immunological and autoimmune considerations of Autism Spectrum Disorders. J Autoimmun. 2013;44:1–7. |
| 86. Gharaibeh HF, Gharaibeh MK. Factors influencing health-related quality of life of thalassaemic Jordanian children. Child Care Health Dev. 2012;38(2):211–8. |
| 87. Ghosh A, Charlton KE, Girdo L, Batterham MJ, McDonald K. Addressing the deficiencies in the evidence-base for primary practice in regional Australia - sentinel practices data sourcing (SPDS) project: a pilot study. BMC Fam Pract. 2013;14:109. |
| 88. Giltenane M, Frazer K, Sheridan A. Evaluating the impact of a quality care-metric on public health nursing practice: protocol for a mixed methods study. J Adv Nurs. 2016; doi: 10.1111/jan.12964. |
| 89. Girard AW, Dzingina C, Akogun O, Mason JB, McFarland DA. Public health interventions, barriers, and opportunities for improving maternal nutrition in Northeast Nigeria. Food Nutr Bull. 2012;33 Suppl 2:S51-70. |
| 90. Girard AW, Self JL, McAuliffe C, Olude O. The effects of household food production strategies on the health and nutrition outcomes of women and young children: a systematic review. Paediatr Perinat Epidemiol. 2012;26 Suppl 1:205–22. |
| 91. González-Saiz F, Roncero C, Martínez-Raga J, Rivera A. Indicadores de calidad asistencial de GESIDA para la atención de personas infectadas por el virus de la inmunodeficiencia adquirida/sida y metadona. Enferm Infecc Microbiol Clin. 2011;29(9):715–6. |
| 92. Gottschalk A, Van Aken H, Zenz M, Standl T. Is anesthesia dangerous? Dtsch Ärzteblatt Int. 2011;108(27):469–74. |
| 93. Hagen A, Entezami M, Gasiorek-Wiens A, Albig M, Becker R, Knoll U, et al. The impact of first trimester screening and early fetal anomaly scan on invasive testing rates in women with advanced maternal age. Ultraschall Med. 2011;32(3):302–6. |
| 94. Haley VB, DiRienzo AG, Lutterloh EC, Stricof RL. Quantifying sources of bias in National Healthcare Safety Network laboratory-identified Clostridium difficile infection rates. Infect Control Hosp Epidemiol. 2014;35(1):1–7. |
| 95. Harder S, Davidsen K, MacBeth A, Lange T, Minnis H, Andersen MS, et al. Wellbeing and resilience: mechanisms of transmission of health and risk in parents with complex mental health problems and their offspring--The WARM Study. BMC Psychiatry. 2015;15:310. |
| 96. Hartmann S, Reimer T, Gerber B. Management of early invasive breast cancer in very young women (<35 years). Clin Breast Cancer. 2011;11(4):196–203. |
| 97. Hartz DL, Foureur M, Tracy SK. Australian caseload midwifery: the exception or the rule. Women Birth. 2012;25(1):39–46. |
| 98. Hazel E, Requejo J, David J, Bryce J. Measuring coverage in MNCH: evaluation of community-based treatment of childhood illnesses through household surveys. PLoS Med. 2013;10(5):e1001384. |
| 99. HEI Collaborative Working Group on Air Pollution, Poverty and H in HCMC, Le TG, Ngo L, Mehta S, Do VD, Thach TQ, et al. Effects of short-term exposure to air pollution on hospital admissions of young children for acute lower respiratory infections in Ho Chi Minh City, Vietnam. Res Rep Health Eff Inst. 2012;(169):5-72-83. |
| 100. Heidari-Bateni G, Norouzi S, Hall M, Brar A, Eghtesady P. Defining the best practice patterns for the neonatal systemic-to-pulmonary artery shunt procedure. J Thorac Cardiovasc Surg. 2014;147(3):869–873.e3. |
| 101. Hemminki E, Long Q, Zhang W-H, Wu Z, Raven J, Tao F, et al. Impact of financial and educational interventions on maternity care: results of cluster randomized trials in rural China, CHIMACA. Matern Child Health J. 2013;17(2):208–21. |
| 102. Hesemann J, Lauer E, Ziska S, Noonan K, Nemeth B, Scott-Schwoerer J, et al. Analysis of maternal risk factors associated with congenital vertebral malformations. Spine (Phila Pa 1976). 2013 1;38(5):E293-8. |
| 103. Hider P, Parker K, von Randow M, Milne B, Lay-Yee R, Davis P. Can patient safety indicators monitor medical and surgical care at New Zealand public hospitals? N Z Med J. 2014;127(1405):32–44. |
| 104. Higgins-Steele A, Waller K, Fotso JC, Vesel L. Peer-driven quality improvement among health workers and traditional birth attendants in Sierra Leone: linkages between providers’ organizational skills and relationships. BMC Health Serv Res. 2015;15 Suppl 1:S4. |
| 105. Hinkley T, Teychenne M, Downing KL, Ball K, Salmon J, Hesketh KD. Early childhood physical activity, sedentary behaviors and psychosocial well-being: a systematic review. Prev Med (Baltim). 2014;62:182–92. |
| 106. Hintz SR, Stevenson DK, Yao Q, Wong RJ, Das A, Van Meurs KP, et al. Is phototherapy exposure associated with better or worse outcomes in 501- to 1000-g-birth-weight infants? Acta Paediatr. 2011;100(7):960–5. |
| 107. Hirshberg A, Srinivas SK. Role of operative vaginal deliveries in prevention of cesarean deliveries. Clin Obstet Gynecol. 2015;58(2):256–62. |
| 108. Holland ML, Yoo B-K, Kitzman H, Chaudron L, Szilagyi PG, Temkin-Greener H. Self-efficacy as a mediator between maternal depression and child hospitalizations in low-income urban families. Matern Child Health J. 2011;15(7):1011–9. |
| 109. Holzer RJ, Gauvreau K, Kreutzer J, Moore JW, McElhinney DB, Bergersen L. Relationship between procedural adverse events associated with cardiac catheterization for congenital heart disease and operator factors: results of a multi-institutional registry (C3PO). Catheter Cardiovasc Interv. 2013;82(3):463–73. |
| 110. Hooker AB, Aydin H, Brolmann HAM, Huirne JAF. Long-term complications and reproductive outcome after the management of retained products of conception: a systematic review. Fertil Steril. 2016;105(1):152–6. |
| 111. Hooper TD, Hibbert PD, Mealing N, Wiles LK, Jaffe A, White L, et al. CareTrack Kids-part 2. Assessing the appropriateness of the healthcare delivered  to Australian children: study protocol for a retrospective medical record review. BMJ Open. 2015;5(4):e007749. |
| 112. Islam MS. Introducing modern technology to promote transparency in health services. Int J Health Care Qual Assur. 2015;28(6):611–20. |
| 113. Jackson S, Gregory KD. Management of the first stage of labor: potential strategies to lower the cesarean delivery rate. Clin Obstet Gynecol. 2015;58(2):217–26. |
| 114. Jacobs SE, Berg M, Hunt R, Tarnow-Mordi WO, Inder TE, Davis PG. Cooling for newborns with hypoxic ischaemic encephalopathy. Cochrane database Syst Rev. 2013;1:CD003311. |
| 115. Jorgensen ML, Young JM, Dobbins TA, Solomon MJ. Assessment of abdominoperineal resection rate as a surrogate marker of hospital quality in rectal cancer surgery. Br J Surg. 2013;100(12):1655–63. |
| 116. Kadhel P, Monnier P, Boucoiran I, Chaillet N, Fraser WD. Organochlorine pollutants and female fertility: a systematic review focusing on in vitro fertilization studies. Reprod Sci. 2012;19(12):1246–59. |
| 117. Karim RM, Abdullah MS, Rahman AM, Alam AM. Identifying influence of perceived quality and satisfaction on the utilization status of the community clinic services; Bangladesh context. Bangladesh Med Res Counc Bull. 2015;41(1):1–12. |
| 118. Kato-Lin Y-C, Krishnamurti L, Padman R, Seltman HJ. Does e-pain plan improve management of sickle cell disease associated vaso-occlusive pain crisis? a mixed methods evaluation. Int J Med Inform. 2014 Nov;83(11):814–24. |
| 119. Kavirayani A, Foster HE, British Society for Paediatric and Adolescent Rheumatology. Paediatric rheumatology practice in the UK benchmarked against the British Society for Paediatric and Adolescent Rheumatology/Arthritis and Musculoskeletal Alliance Standards of Care for juvenile idiopathic arthritis. Rheumatology (Oxford). 2013;52(12):2203–7. |
| 120. Kawicka A, Regulska-Ilow B. How nutritional status, diet and dietary supplements can affect autism. A review. Rocz Państwowego Zakładu Hig. 2013;64(1):1–12. |
| 121. Kersten FAM, Hermens RPGM, Braat DDM, Hoek A, Mol BWJ, Goddijn M, et al. Overtreatment in couples with unexplained infertility. Hum Reprod. 2015;30(1):71–80. |
| 122. Khanna R, Karikalan N, Mishra AK, Agarwal A, Bhattacharya M, Das JK. Repository on maternal child health: health portal to improve access to information on maternal child health in India. BMC Public Health. 2013;13:2. |
| 123. Kilian A, Koenker H, Baba E, Onyefunafoa EO, Selby RA, Lokko K, et al. Universal coverage with insecticide-treated nets - applying the revised indicators for ownership and use to the Nigeria 2010 malaria indicator survey data. Malar J. 2013;12:314. |
| 124. Klevens J, Trick WE, Kee R, Angulo F, Garcia D, Sadowski LS. Concordance in the measurement of quality of life and health indicators between two methods of computer-assisted interviews: self-administered and by telephone. Qual Life Res. 2011;20(8):1179–86. |
| 125. Knoblauch AM, Hodges MH, Bah MS, Kamara HI, Kargbo A, Paye J, et al. Changing patterns of health in communities impacted by a bioenergy project in Northern Sierra Leone. Int J Environ Res Public Health. 2014;11(12):12997–3016. |
| 126. Knoche LL, Sheridan SM, Clarke BL, Edwards CP, Marvin CA, Cline KD, et al. GETTING READY: RESULTS OF A RANDOMIZED TRIAL OF A RELATIONSHIP-FOCUSED INTERVENTION ON THE PARENT-INFANT RELATIONSHIP IN RURAL EARLY HEAD START. Infant Ment Health J. 2012;33(5):439–58. |
| 127. Korst LM, Fridman M, Estarziau M, Gregory KD, Mitchell C. The Feasibility of Tracking Elective Deliveries Prior to 39 Gestational Weeks: Lessons From Three California Projects. Matern Child Health J. 2015;19(10):2128–37. |
| 128. Kvist LJ, Damiati N, Rosenqvist J, Sandin-Bojo A-K. Measuring the quality of documented care given by Swedish midwives during birth. Midwifery. 2011;27(6):e188-94. |
| 129. Lago P, Boccuzzo G, Garetti E, Pirelli A, Pieragostini L, Merazzi D, et al. Pain management during invasive procedures at Italian NICUs: has anything changed in the last 5 years? J Matern Fetal Neonatal Med. 2012;25 Suppl 4:148–50. |
| 130. LaKind JS, Goodman M, Barr DB, Weisel CP, Schoeters G. Lessons learned from the application of BEES-C: Systematic assessment of study quality of epidemiologic research on BPA, neurodevelopment, and respiratory health. Environ Int. 2015;80:41–71. |
| 131. Lannon C, Peterson LE, Goudie A. Quality measures for the care of children with otitis media with effusion. Pediatrics. 2011;127(6):e1490-7. |
| 132. Lassi ZS, Cometto G, Huicho L, Bhutta ZA. Quality of care provided by mid-level health workers: systematic review and meta-analysis. Bull World Health Organ. 2013;91(11):824–833I. |
| 133. Lazzerini M, Seward N, Lufesi N, Banda R, Sinyeka S, Masache G, et al. Mortality and its risk factors in Malawian children admitted to hospital with clinical pneumonia, 2001-12: a retrospective observational study. Lancet Glob Heal. 2016;4(1):e57-68. |
| 134. LeBlanc AG, Spence JC, Carson V, Connor Gorber S, Dillman C, Janssen I, et al. Systematic review of sedentary behaviour and health indicators in the early years (aged 0-4 years). Appl Physiol Nutr Metab = Physiol Appl Nutr métabolisme. 2012;37(4):753–72. |
| 135. Lee W-C, Chen T-J. Quantifying morbidity burdens and medical utilization of children with intellectual disabilities in Taiwan: a nationwide study using the ACG case-mix adjustment system. Res Dev Disabil. 2012;33(4):1270–8. |
| 136. Leger J, Ecosse E, Roussey M, Lanoe JL, Larroque B. Subtle health impairment and socioeducational attainment in young adult patients  with congenital hypothyroidism diagnosed by neonatal screening: a longitudinal population-based cohort study. J Clin Endocrinol Metab. 2011;96(6):1771–82. |
| 137. Leong PM, Gussy MG, Barrow S-YL, de Silva-Sanigorski A, Waters E. A systematic review of risk factors during first year of life for early childhood caries. Int J Paediatr Dent. 2013;23(4):235–50. |
| 138. Leroy JL, Ruel M, Frongillo EA, Harris J, Ballard TJ. Measuring the Food Access Dimension of Food Security: A Critical Review and Mapping of Indicators. Food Nutr Bull. 2015;36(2):167–95. |
| 139. Li MY, Kelly J, Subhi R, Were W, Duke T. Global use of the WHO pocket book of hospital care for children. Paediatr Int Child Health. 2013;33(1):4–17. |
| 140. Little SE, Caughey AB. Induction of Labor and Cesarean: What is the True Relationship? Clin Obstet Gynecol. 2015;58(2):269–81. |
| 141. MacGillivray S, Fahey T, McGuire W. Lactose avoidance for young children with acute diarrhoea. Cochrane database Syst Rev. 2013;10:CD005433. |
| 142. Mackey ER, Herbert L, Monaghan M, Cogen F, Wang J, Streisand R. The Feasibility of a Pilot Intervention for Parents of Young Children Newly Diagnosed with Type 1 Diabetes. Clin Pract Pediatr Psychol. 2016;4(1):35–50. |
| 143. Magge H, Anatole M, Cyamatare FR, Mezzacappa C, Nkikabahizi F, Niyonzima S, et al. Mentoring and quality improvement strengthen integrated management of childhood illness implementation in rural Rwanda. Arch Dis Child. 2015;100(6):565–70. |
| 144. Mahadevan U, Matro R. Care of the Pregnant Patient With Inflammatory Bowel Disease. Obstet Gynecol. 2015;126(2):401–12. |
| 145. Målqvist M, Yuan B, Trygg N, Selling K, Thomsen S. Targeted interventions for improved equity in maternal and child health in low- and middle-income settings: a systematic review and meta-analysis. PLoS One. 2013;8(6):e66453. |
| 146. Markova V, Norgaard A, Jorgensen KJ, Langhoff-Roos J. Treatment for women with postpartum iron deficiency anaemia. Cochrane database Syst Rev. 2015;8:CD010861. |
| 147. Martinelli S, Gatelli I, Proto A. SpO2 and retinopathy of prematurity: state of the art. J Matern Fetal Neonatal Med. 2012;25 Suppl 4:108–10. |
| 148. Mbuagbaw L, Medley N, Darzi AJ, Richardson M, Habiba Garga K, Ongolo-Zogo P. Health system and community level interventions for improving antenatal care coverage and health outcomes. Cochrane database Syst Rev. 2015;12:CD010994. |
| 149. Mbwele B, Reddy E, Reyburn H. A rapid assessment of the quality of neonatal healthcare in Kilimanjaro region, northeast Tanzania. BMC Pediatr. 2012;12:182. |
| 150. McBride N, Johnson S. Fathers’ Role in Alcohol-Exposed Pregnancies: Systematic Review of Human Studies. Am J Prev Med. 2016; doi: 10.1016/j.amepre.2016.02.009. |
| 151. McConachie H, Parr JR, Glod M, Hanratty J, Livingstone N, Oono IP, et al. Systematic review of tools to measure outcomes for young children with autism spectrum disorder. Health Technol Assess. 2015;19(41):1–506. |
| 152. McKay H, Mitchell IA, Sinn K, Mugridge H, Lafferty T, Van Leuvan C, et al. Effect of a multifaceted intervention on documentation of vital signs and staff communication regarding deteriorating paediatric patients. J Paediatr Child Health. 2013;49(1):48–56. |
| 153. Mehmood A, Razzak JA, Kabir S, Mackenzie EJ, Hyder AA. Development and pilot implementation of a locally developed Trauma Registry: lessons learnt in a low-income country. BMC Emerg Med. 2013;13:4. |
| 154. Mielke RT, Kaiser D, Centuolo R. Interconception care for women with prior gestational diabetes mellitus. J Midwifery Womens Health. 2013;58(3):303–12. |
| 155. Milman N. Postpartum anemia I: definition, prevalence, causes, and consequences. Ann Hematol. 2011;90(11):1247–53. |
| 156. Mincarone P, Leo CG, Sabina S, Costantini D, Cozzolino F, Wong JB, et al. Evaluating reporting and process quality of publications on UNHS: a systematic review of programmes. BMC Pediatr. 2015;15:86. |
| 157. Mofid LS, Casapia M, Montresor A, Rahme E, Fraser WD, Marquis GS, et al. Maternal Deworming Research Study (MADRES) protocol: a double-blind, placebo-controlled randomised trial to determine the effectiveness of deworming in the immediate postpartum period. BMJ Open. 2015;5(6):e008560. |
| 158. Mokdad AH, Colson KE, Zuniga-Brenes P, Rios-Zertuche D, Palmisano EB, Alfaro-Porras E, et al. Salud Mesoamerica 2015 Initiative: design, implementation, and baseline findings. Popul Health Metr. 2015;13(1):3. |
| 159. Moore D, Ayers S. A review of postnatal mental health websites: help for healthcare professionals and patients. Arch Womens Ment Health. 2011;14(6):443–52. |
| 160. Morfaw FLI, Thabane L, Mbuagbaw LCE, Nana PN. Male participation in prevention programmes of mother to child transmission of HIV: a protocol for a systematic review to identify barriers, facilitators and reported interventions. Syst Rev. 2012;1:13. |
| 161. Morris C, Janssens A, Shilling V, Allard A, Fellowes A, Tomlinson R, et al. Meaningful health outcomes for paediatric neurodisability: Stakeholder prioritisation and appropriateness of patient reported outcome measures. Health Qual Life Outcomes. 2015;13:87. |
| 162. Morse RB, Hall M, Fieldston ES, McGwire G, Anspacher M, Sills MR, et al. Hospital-level compliance with asthma care quality measures at children’s hospitals and subsequent asthma-related outcomes. JAMA. 2011;306(13):1454–60. |
| 163. Moser JW, Applegate KE. Imaging and insurance: do the uninsured get less imaging in emergency departments? J Am Coll Radiol. 2012;9(1):50–7. |
| 164. Moura LMVR, Mendez DY, De Jesus J, Andrade RA, Hoch DB. Quality care in epilepsy: Women’s counseling and its association with folic acid  prescription or recommendation. Epilepsy Behav. 2015;44:151–4. |
| 165. Mukherjee S, Ghosh S, Goswami DN, Samanta A. Performance evaluation of PPTCT (Prevention of parent to child transmission of HIV) programme: an experience from West Bengal. Indian J Med Res. 2012;136(6):1011–9. |
| 166. Naja F, Nasreddine L, Al Thani AA, Yunis K, Clinton M, Nassar A, et al. Study protocol: Mother and Infant Nutritional Assessment (MINA) cohort study in Qatar and Lebanon. BMC Pregnancy Childbirth. 2016;16(1):98. |
| 167. Nalwadda Kayemba C, Naamala Sengendo H, Ssekitooleko J, Kerber K, Källander K, Waiswa P, et al. Introduction of newborn care within integrated community case management in Uganda. Am J Trop Med Hyg. 2012;87 Suppl 5:46–53. |
| 168. Neuberger F, Wennike N. Problem based review: pulmonary embolism in pregnancy. Acute Med. 2013;12(4):239–45. |
| 169. Newcombe PA, Sheffield JK, Chang AB. Parent cough-specific quality of life: development and validation of a short form. J Allergy Clin Immunol. 2013;131(4):1069–74. |
| 170. Nowakowski A, Cybulski M, Sliwczynski A, Chil A, Teter Z, Seroczynski P, et al. The implementation of an organised cervical screening programme in Poland: an analysis of the adherence to European guidelines. BMC Cancer. 2015;15:279. |
| 171. O’Connor MJ, Laugeson EA, Mogil C, Lowe E, Welch-Torres K, Keil V, et al. Translation of an evidence-based social skills intervention for children with prenatal alcohol exposure in a community mental health setting. Alcohol Clin Exp Res. 2012;36(1):141–52. |
| 172. O’Donnell E, Utz B, Khonje D, van den Broek N. “At the right time, in the right way, with the right resources”: perceptions of the quality of care provided during childbirth in Malawi. BMC Pregnancy Childbirth. 2014;14:248. |
| 173. Ohannessian A, Loundou A, Courbière B, Cravello L, Agostini A. Ovarian responsiveness in women receiving fertility treatment after methotrexate for ectopic pregnancy: a systematic review and meta-analysis. Hum Reprod. 2014;29(9):1949–56. |
| 174. Olney RS, Ailes EC, Sontag MK. Detection of critical congenital heart defects: Review of contributions from prenatal and newborn screening. Semin Perinatol. 2015;39(3):230–7. |
| 175. Olsen J, Ramlau-Hansen CH. Epidemiologic methods for investigating male fecundity. Asian J Androl. 2014;16(1):17–22. |
| 176. Oono IP, Honey EJ, McConachie H. Parent-mediated early intervention for young children with autism spectrum disorders (ASD). Cochrane database Syst Rev. 2013;4:CD009774. |
| 177. Osrin D, Das S, Bapat U, Alcock GA, Joshi W, More NS. A rapid assessment scorecard to identify informal settlements at higher maternal and child health risk in Mumbai. J Urban Health. 2011;88(5):919–32. |
| 178. Oster ME, Strickland MJ, Mahle WT. Impact of prior hospital mortality versus surgical volume on mortality following  surgery for congenital heart disease. J Thorac Cardiovasc Surg. 2011;142(4):882–6. |
| 179. Palmer C, Bycroft J, Healey K, Field A, Ghafel M. Can formal collaborative methodologies improve quality in primary health care in New Zealand? Insights from the EQUIPPED Auckland Collaborative. J Prim Health Care. 2012;4(4):328–36. |
| 180. Pati S, Wong AT, Calixte RE, Ludwig J, Zeigler A, Localio AR, et al. Medicaid and CHIP retention among children in 12 states. Acad Pediatr. 2015;15(3):249–57. |
| 181. Patrick SW, Schumacher RE, Davis MM. Methods of mortality risk adjustment in the NICU: a 20-year review. Pediatrics. 2013 Mar;131 Suppl:S68-74. |
| 182. Paul J, Jordan R, Duty S, Engstrom JL. Improving satisfaction with care and reducing length of stay in an obstetric triage unit using a nurse-midwife-managed model of care. J Midwifery Womens Health. 2013;58(2):175–81. |
| 183. Penny KI, Smith GD. The use of data-mining to identify indicators of health-related quality of life in patients with irritable bowel syndrome. J Clin Nurs. 2012;21(19–20):2761–71. |
| 184. Perez-Lu JE, Iguiniz Romero R, Bayer AM, Garcia PJ. [Wawared Peru: reducing health inequities and improving maternal health by improving information systems in health]. Rev Peru Med Exp Salud Publica. 2015;32(2):373–7. |
| 185. Piatt JH, Freibott CE. Quality measurement in the shunt treatment of hydrocephalus: analysis and risk adjustment of the Revision Quotient. J Neurosurg Pediatr. 2014;14(1):48–54. |
| 186. Pinheiro JMB, Furdon SA, Boynton S, Dugan R, Reu-Donlon C, Jensen S. Decreasing hypothermia during delivery room stabilization of preterm neonates. Pediatrics. 2014;133(1):e218-26. |
| 187. Radeva-Petrova D, Kayentao K, ter Kuile FO, Sinclair D, Garner P. Drugs for preventing malaria in pregnant women in endemic areas: any drug regimen versus placebo or no treatment. Cochrane database Syst Rev. 2014;10:CD000169. |
| 188. Rajmil L, Fernandez de Sanmamed M-J, Choonara I, Faresjö T, Hjern A, Kozyrskyj AL, et al. Impact of the 2008 economic and financial crisis on child health: a systematic review. Int J Environ Res Public Health. 2014;11(6):6528–46. |
| 189. Ramaiya A, Kiss L, Baraitser P, Mbaruku G, Hildon Z. A systematic review of risk factors for neonatal mortality in adolescent mother’s in Sub Saharan Africa. BMC Res Notes. 2014;7:750. |
| 190. Ramirez-Luzuriaga MJ, Unar-Munguia M, Rodriguez-Ramirez S, Rivera JA, Gonzalez de Cosio T. A Food Transfer Program without a Formal Education Component Modifies Complementary Feeding Practices in Poor Rural Mexican Communities. J Nutr. 2016;146(1):107–13. |
| 191. Ramphul M, Ooi P V, Burke G, Kennelly MM, Said SAT, Montgomery AA, et al. Instrumental delivery and ultrasound : a multicentre randomised controlled trial of ultrasound assessment of the fetal head position versus standard care as an approach to prevent morbidity at instrumental delivery. BJOG. 2014;121(8):1029–38. |
| 192. Richard F, Hercot D, Ouedraogo C, Delvaux T, Samake S, van Olmen J, et al. Sub-Saharan Africa and the health MDGs: the need to move beyond the “quick impact” model. Reprod Health Matters. 2011;19(38):42–55. |
| 193. Rinke ML, Bundy DG, Abdullah F, Colantuoni E, Zhang Y, Miller MR. State-Mandated Hospital Infection Reporting Is Not Associated With Decreased Pediatric Health Care-Associated Infections. J Patient Saf. 2015;11(3):123–34. |
| 194. Riskin-Mashiah S, Auslander R. Quality of medical care in diabetic women undergoing fertility treatment: we should do better! Diabetes Care. 2011;34(10):2164–9. |
| 195. Roberts YH, Ferguson M, Crusto CA. Exposure to traumatic events and health-related quality of life in preschool-aged children. Qual Life Res. 2013;22(8):2159–68. |
| 196. Robson M, Hartigan L, Murphy M. Methods of achieving and maintaining an appropriate caesarean section rate. Best Pract Res Clin Obstet Gynaecol. 2013;27(2):297–308. |
| 197. Romley JA, Chen AY, Goldman DP, Williams R. Hospital costs and inpatient mortality among children undergoing surgery for congenital heart disease. Health Serv Res. 2014;49(2):588–608. |
| 198. Rosen BS, Maddox PJ, Ray N. A position paper on how cost and quality reforms are changing healthcare in America: focus on nutrition. JPEN J Parenter Enteral Nutr. 2013;37(6):796–801. |
| 199. Ryman TK, Briere EC, Cartwright E, Schlanger K, Wannemuehler KA, Russo ET, et al. Integration of routine vaccination and hygiene interventions: a comparison of 2 strategies in Kenya. J Infect Dis. 2012;205 Suppl:S65-76. |
| 200. Salehi Z, Mokhtari Nouri J, Khademolhoseyni SM, Ebadi A. The effect of education and implementation of evidence-based nursing guidelines on infants’ weight gaining in NICU. Glob J Health Sci. 2015;7(2):148–53. |
| 201. Scholte M, Neeleman-van der Steen CWM, Hendriks EJM, Nijhuis-van der Sanden MWG, Braspenning J. Evaluating quality indicators for physical therapy in primary care. Int J Qual Health Care. 2014;26(3):261–70. |
| 202. Schubert KG, Cavarocchi N. The value of advocacy in obstetrics and maternal-fetal medicine. Curr Opin Obstet Gynecol. 2012;24(6):453–7. |
| 203. Senosy W, Zain AE, Abdel-Razek A-RK, Uchiza M, Tameoka N, Izaike Y, et al. Association between energy status early postpartum and subsequent embryonic mortality in high-yielding recipient cows. Anim Sci J = Nihon chikusan Gakkaihō. 2012;83(4):284–90. |
| 204. Sentell T, Chang A, Cheng Y, Miyamura J. Maternal quality and safety outcomes for Asians and Pacific Islanders in Hawai’i: an observational study from five years of statewide data. BMC Pregnancy and Childbirth. 2014;14:298. |
| 205. Setodji CM, Le V-N, Schaack D. Using generalized additive modeling to empirically identify thresholds within the ITERS in relation to toddlers’ cognitive development. Dev Psychol. 2013;49(4):632–45. |
| 206. Shamsi MB, Imam SN, Dada R. Sperm DNA integrity assays: diagnostic and prognostic challenges and implications in management of infertility. J Assist Reprod Genet. 2011;28(11):1073–85. |
| 207. Shanmugam G, Clark LL, Burton HJ, Warren AE, O’Blenes SB, Hancock Friesen CL. Improving and standardizing capture of pediatric cardiac surgical complications. J Thorac Cardiovasc Surg. 2012;144(3):570–6. |
| 208. Sharpe S, Kool B, Shepherd M, Dalziel S, Ameratunga S. Mild traumatic brain injury: improving quality of care in the paediatric emergency department setting. J Paediatr Child Health. 2012;48(2):170–6. |
| 209. Sharshiner R, Silver RM. Management of fetal malpresentation. Clin Obstet Gynecol. 2015;58(2):246–55. |
| 210. Shmueli A. On the calculation of the Israeli risk adjustment rates. Eur J Health Econ. 2015;16(3):271–7. |
| 211. Shovlin CL. Pulmonary arteriovenous malformations. Am J Respir Crit Care Med. 2014;190(11):1217–28. |
| 212. Sibley LM, Glazier RH. Evaluation of the equity of age-sex adjusted primary care capitation payments in Ontario, Canada. Health Policy. 2012;104(2):186–92. |
| 213. Siegfried N, Irlam JH, Visser ME, Rollins NN. Micronutrient supplementation in pregnant women with HIV infection. Cochrane database Syst Rev. 2012;3:CD009755. |
| 214. Simms RA, Ping H, Yelland A, Beringer AJ, Fox R, Draycott TJ. Development of maternity dashboards across a UK health region; current practice, continuing problems. Eur J Obstet Gynecol Reprod Biol. 2013;170(1):119–24. |
| 215. Simms RA, Yelland A, Ping H, Beringer AJ, Draycott TJ, Fox R. Using data and quality monitoring to enhance maternity outcomes: a qualitative study of risk managers’ perspectives. BMJ Qual Saf. 2014;23(6):457–64. |
| 216. Sjetne IS, Iversen HH, Kjollesdal JG. A questionnaire to measure women’s experiences with pregnancy, birth and postnatal care: instrument development and assessment following a national survey in Norway. BMC Pregnancy Childbirth. 2015;15:182. |
| 217. Slattery J, Morgan A, Douglas J. Early sucking and swallowing problems as predictors of neurodevelopmental outcome in children with neonatal brain injury: a systematic review. Dev Med Child Neurol. 2012;54(9):796–806. |
| 218. Smith RF, Oultram J, Dobson H. Herd monitoring to optimise fertility in the dairy cow: making the most of herd records, metabolic profiling and ultrasonography (research into practice). Animal. 2014;8 Suppl 1:185–98. |
| 219. Smyth AR, Bell SC, Bojcin S, Bryon M, Duff A, Flume P, et al. European Cystic Fibrosis Society Standards of Care: Best Practice guidelines. J Cyst Fibros. 2014 May;13 Suppl 1:S23-42. |
| 220. Sparks AET. Human embryo cryopreservation-methods, timing, and other considerations for optimizing an embryo cryopreservation program. Semin Reprod Med. 2015;33(2):128–44. |
| 221. St Germaine-Smith C, Liu M, Quan H, Wiebe S, Jette N. Development of an epilepsy-specific risk adjustment comorbidity index. Epilepsia. 2011;52(12):2161–7. |
| 222. Stern M, Bertrand DP, Bignamini E, Corey M, Dembski B, Goss CH, et al. European Cystic Fibrosis Society Standards of Care: Quality Management in cystic fibrosis. J Cyst Fibros. 2014;13 Suppl 1:S43-59. |
| 223. Stevens B, Yamada J, Lee GY, Ohlsson A. Sucrose for analgesia in newborn infants undergoing painful procedures. Cochrane database Syst Rev. 2013;1:CD001069. |
| 224. Sulkowski JP, Cooper JN, McConnell PI, Pasquali SK, Shah SS, Minneci PC, et al. Variability in noncardiac surgical procedures in children with congenital heart disease. J Pediatr Surg. 2014;49(11):1564–9. |
| 225. Tabak YP, Sun X, Hyde L, Yaitanes A, Derby K, Johannes RS. Using enriched observational data to develop and validate age-specific mortality risk adjustment models for hospitalized pediatric patients. Med Care. 2013;51(5):437–45. |
| 226. Tahir MA, Dmitrieva O, de Lusignan S, van Vlymen J, Chan T, Golmohamad R, et al. Confidence and quality in managing CKD compared with other cardiovascular diseases and diabetes mellitus: a linked study of questionnaire and routine primary care data. BMC Fam Pract. 2011;12:83. |
| 227. Tanner-Smith EE, Steinka-Fry KT, Lipsey MW. The effects of CenteringPregnancy group prenatal care on gestational age, birth weight, and fetal demise. Matern Child Health J. 2014;18(4):801–9. |
| 228. Tanner-Smith EE, Steinka-Fry KT, Lipsey MW. Effects of CenteringPregnancy group prenatal care on breastfeeding outcomes. J Midwifery Womens Health. 2013;58(4):389–95. |
| 229. Thompson MA, Aberg JA, Hoy JF, Telenti A, Benson C, Cahn P, et al. Antiretroviral treatment of adult HIV infection: 2012 recommendations of the International Antiviral Society-USA panel. JAMA. 2012 25;308(4):387–402. |
| 230. Thorne-Lyman AL, Fawzi WW. Vitamin A and carotenoids during pregnancy and maternal, neonatal and infant health outcomes: a systematic review and meta-analysis. Paediatr Perinat Epidemiol. 2012;26 Suppl 1:36–54. |
| 231. Timmons BW, Leblanc AG, Carson V, Connor Gorber S, Dillman C, Janssen I, et al. Systematic review of physical activity and health in the early years (aged 0-4 years). Appl Physiol Nutr Metab = Physiol Appl Nutr métabolisme. 2012;37(4):773–92. |
| 232. To T, Guan J, Zhu J, Lougheed MD, Kaplan A, Tamari I, et al. Quality of asthma care under different primary care models in Canada: a population-based study. BMC Fam Pract. 2015;16:19. |
| 233. Tobollik M, Razum O, Wintermeyer D, Plass D. Burden of Outdoor Air Pollution in Kerala, India-A First Health Risk Assessment at State Level. Int J Environ Res Public Health. 2015;12(9):10602–19. |
| 234. Toft G. Persistent organochlorine pollutants and human reproductive health. Dan Med J. 2014;61(11):B4967. |
| 235. Tonguet-Papucci A, Huybregts L, Ait Aissa M, Huneau J-F, Kolsteren P. The MAM’Out project: a randomized controlled trial to assess multiannual and seasonal cash transfers for the prevention of acute malnutrition in children under 36 months in Burkina Faso. BMC Public Health. 2015;15:762. |
| 236. Trivedi D. Cochrane review summary: specialised antenatal clinics for women with a multiple pregnancy for improving maternal and infant outcomes. Prim Health Care Res Dev. 2014 15;15(1):3–4. |
| 237. Tryphonopoulos PD, Letourneau N, Ditommaso E. Attachment and caregiver-infant interaction: a review of observational-assessment tools. Infant Ment Health J. 2014;35(6):642–56. |
| 238. Turley R, Saith R, Bhan N, Rehfuess E, Carter B. Slum upgrading strategies involving physical environment and infrastructure interventions and their effects on health and socio-economic outcomes. Cochrane database Syst Rev. 2013;1:CD010067. |
| 239. Turner MJ. The use of quality control performance charts to analyze cesarean delivery rates  nationally. Int J Gynaecol Obstet. 2011;113(3):175–7. |
| 240. van der Kleij R, Coster N, Verbiest M, van Assema P, Paulussen T, Reis R, et al. Implementation of intersectoral community approaches targeting childhood obesity: a systematic review. Obes Rev. 2015;16(6):454–72. |
| 241. van der Wal MBA, Tuinebreijer WE, Lundgren-Nilsson Å, Middelkoop E, van Zuijlen PPM. Differential item functioning in the Observer Scale of the POSAS for different scar types. Qual Life Res. 2014;23(7):2037–45. |
| 242. Vanderkruik RC, Tunçalp Ö, Chou D, Say L. Framing maternal morbidity: WHO scoping exercise. BMC Pregnancy Childbirth. 2013;13:213. |
| 243. Vas J, Aranda-Regules JM, Modesto M, Ramos-Monserrat M, Barón M, Aguilar I, et al. Using moxibustion in primary healthcare to correct non-vertex presentation: a multicentre randomised controlled trial. Acupunct Med. 2013;31(1):31–8. |
| 244. Vavilala MS, Kernic MA, Wang J, Kannan N, Mink RB, Wainwright MS, et al. Acute care clinical indicators associated with discharge outcomes in children with severe traumatic brain injury. Crit Care Med. 2014;42(10):2258–66. |
| 245. Verrips G, Brouwer L, Vogels T, Taal E, Drossaert C, Feeny D, et al. Long term follow-up of health-related quality of life in young adults born very preterm or with a very low birth weight. Health Qual Life Outcomes. 2012;10:49. |
| 246. Vieira C, Portela A, Miller T, Coast E, Leone T, Marston C. Increasing the use of skilled health personnel where traditional birth attendants were providers of childbirth care: a systematic review. PLoS One. 2012;7(10):e47946. |
| 247. Vlayen J, Vrijens F, Devriese S, Beirens K, Van Eycken E, Stordeur S. Quality indicators for testicular cancer: a population-based study. Eur J Cancer. 2012;48(8):1133–40. |
| 248. Wagner J, Hanson C, Anderson-Berry A. Considerations in meeting protein needs of the human milk-fed preterm infant. Adv Neonatal Care. 2014;14(4):281–9. |
| 249. Warthon-Medina M, Moran VH, Stammers A-L, Dillon S, Qualter P, Nissensohn M, et al. Zinc intake, status and indices of cognitive function in adults and children: a systematic review and meta-analysis. Eur J Clin Nutr. 2015;69(6):649–61. |
| 250. Weiss WM, Rahman MDH, Solomon R, Ward D. Determinants of performance of supplemental immunization activities for polio eradication in Uttar Pradesh, India: social mobilization activities of the Social mobilization Network (SM Net) and Core Group Polio Project (CGPP). BMC Infect Dis. 2013;13:17. |
| 251. Wen J, Jiang J, Ding C, Dai J, Liu Y, Xia Y, et al. Birth defects in children conceived by in vitro fertilization and intracytoplasmic sperm injection: a meta-analysis. Fertil Steril. 2012;97(6):1331-7-4. |
| 252. Wiebe HW, Boule NG, Chari R, Davenport MH. The effect of supervised prenatal exercise on fetal growth: a meta-analysis. Obstet Gynecol. 2015;125(5):1185–94. |
| 253. Wiles LK, Hooper TD, Hibbert PD, White L, Mealing N, Jaffe A, et al. CareTrack Kids-part 1. Assessing the appropriateness of healthcare delivered to Australian children: study protocol for clinical indicator development. BMJ Open. 2015;5(4):e007748. |
| 254. Wilson S, Bremner A, Hauck Y, Finn J. The effect of nurse staffing on clinical outcomes of children in hospital: a systematic review. Int J Evid Based Healthc. 2011;9(2):97–121. |
| 255. Winkler MS, Knoblauch AM, Righetti AA, Divall MJ, Koroma MM, Fofanah I, et al. Baseline health conditions in selected communities of northern Sierra Leone as revealed by the health impact assessment of a biofuel project. Int Health. 2014;6(3):232–41. |
| 256. Wolthuis AM, Tomassetti C. Multidisciplinary laparoscopic treatment for bowel endometriosis. Best Pract Res Clin Gastroenterol. 2014;28(1):53–67. |
| 257. Xu F, Sullivan EA, Madden RC, Black D, Pulver LRJ. Improvement of maternal Aboriginality in NSW birth data. BMC Med Res Methodol. 2012;12:8. |
| 258. Yao DF, Weinberg AC, Penna FJ, Huang L, Freilich DA, Minnillo BJ, et al. Quality of life in children with vesicoureteral reflux as perceived by children and parents. J Pediatr Urol. 2011;7(3):261–5. |
| 259. Zhao L-P, Gerdin M, Westman L, Rodriguez-Llanes JM, Wu Q, van den Oever B, et al. Hospital stay as a proxy indicator for severe injury in earthquakes: a retrospective analysis. PLoS One. 2013;8(4):e61371. |
| 260. Practice Bulletin No. 139: premature rupture of membranes. Obstet Gynecol. 2013; doi: 10.1097/01.AOG.0000435415.21944.8f |
| 261. The Joint Commission. Questions and answers: the perinatal care core measure set. Jt Comm Perspect. 2013 Nov;33(11):12–4. |
|  |
| **Full text articles excluded** |
| Not described indicators |
|  |
| 1. American Diabetes Association. Standards of Medical Care in Diabetes--2012. Diabetes Care. 2012;35(Supplement_1):S11–63. |
| 2. Anderka M, Mai CT, Romitti PA, Copeland G, Isenburg J, Feldkamp ML, et al. Development and implementation of the first national data quality standards for population-based birth defects surveillance programs in the United States. BMC Public Health. 2015;15:925. |
| 3. Baaqeel H, Baaqeel R. Timing of administration of prophylactic antibiotics for caesarean section: a systematic review and meta-analysis. BJOG. 2013;120(6):661–9. |
| 4. Brady M, Manning J. Lessons from reproductive health to inform multipurpose prevention technologies: don’t reinvent the wheel. Antiviral Res. 2013;100 Suppl:S25-31. |
| 5. Brenner S, Muula AS, Robyn PJ, Bärnighausen T, Sarker M, Mathanga DP, et al. Design of an impact evaluation using a mixed methods model - an explanatory assessment of the effects of results-based financing mechanisms on maternal healthcare services in Malawi. BMC Health Serv Res. 2014;14(1):1–33. |
| 6. Chhabra P. Maternal near miss: an indicator for maternal health and maternal care. Indian J Community Med. 2014;39(3):132–7. |
| 7. Colais P, Fantini MP, Fusco D, Carretta E, Stivanello E, Lenzi J, et al. Risk adjustment models for interhospital comparison of CS rates using Robson’s ten group classification system and other socio-demographic and clinical variables. BMC Pregnancy Childbirth. 2012;12:54. |
| 8. Corsi DJ, Neuman M, Finlay JE, Subramanian S V. Demographic and health surveys: a profile. Int J Epidemiol. 2012;41(6):1602–13. |
| 9. Delnord M, Szamotulska K, Hindori-Mohangoo AD, Blondel B, Macfarlane AJ, Dattani N, et al. Linking databases on perinatal health: a review of the literature and current practices in Europe. Eur J Public Health. 2016; 26(3), 422-430. |
| 10. Dettrick Z, Firth S, Jimenez Soto E. Do strategies to improve quality of maternal and child health care in lower and middle income countries lead to improved outcomes? A review of the evidence. PLoS One. 2013;8(12):e83070. |
| 11. Doumouchtsis SK, Nikolopoulos K, Talaulikar V, Krishna A, Arulkumaran S. Menstrual and fertility outcomes following the surgical management of postpartum haemorrhage: a systematic review. BJOG. 2014;121(4):382–8. |
| 12. Ejigu T, Woldie M, Kifle Y. Quality of antenatal care services at public health facilities of Bahir-Dar special zone, Northwest Ethiopia. BMC Health Serv Res. 2013;13:443. |
| 13. Hancioglu A, Arnold F. Measuring coverage in MNCH: tracking progress in health for women and children using DHS and MICS household surveys. PLoS Med. 2013;10(5):e1001391. |
| 14. Hinton CF, Feuchtbaum L, Kus CA, Kemper AR, Berry SA, Levy-Fisch J, et al. What questions should newborn screening long-term follow-up be able to answer? A statement of the US Secretary for Health and Human Services’ Advisory Committee on Heritable Disorders in Newborns and Children. Genet Med. 2011;13(10):861–5. |
| 15. Inamdar AS, Croucher RE, Chokhandre MK, Mashyakhy MH, Marinho VCC. Maternal Smokeless Tobacco Use in Pregnancy and Adverse Health Outcomes in Newborns: A Systematic Review. Nicotine Tob Res. 2015;17(9):1058–66. |
| 16. Ismail S, Shafiq F, Malik A. Technique of anaesthesia for different grades of caesarean section: a cross-sectional study. J Pak Med Assoc. 2012;62(4):363–7. |
| 17. Jensen HA, Brown KL, Pagel C, Barron DJ, Franklin RCG. Mortality as a measure of quality of care in infants with congenital cardiovascular malformations following surgery. Br Med Bull. 2014;111(1):5–15. |
| 18. Kindra G, Coutsoudis A, Esposito F. Effect of nutritional supplementation of breastfeeding HIV positive mothers on maternal and child health: findings from a randomized controlled clinical trial. BMC Public Health. 2011;11:946. |
| 19. Kinney M V, Cocoman O, Dickson KE, Daelmans B, Zaka N, Rhoda NR, et al. Implementation of the Every Newborn Action Plan: Progress and lessons learned. Semin Perinatol. 2015;39(5):326–37. |
| 20. Langley-Evans SC. Nutrition in early life and the programming of adult disease: a review. J Hum Nutr Diet. 2015;28 Suppl 1:1–14. |
| 21. Liu L-C, Wang Y-C, Yu M-H, Su H-Y. Major risk factors for stillbirth in different trimesters of pregnancy--a systematic review. Taiwan J Obstet Gynecol. 2014;53(2):141–5. |
| 22. Main EK, Goffman D, Scavone BM, Low LK, Bingham D, Fontaine PL, et al. National Partnership for Maternal Safety: consensus bundle on obstetric hemorrhage. Anesth Analg. 2015;121(1):142–8. |
| 23. Maso G, Alberico S, Monasta L, Ronfani L, Montico M, Businelli C, et al. The application of the Ten Group classification system (TGCS) in caesarean delivery case mix adjustment. A multicenter prospective study. PLoS One. 2013;8(6):e62364. |
| 24. Melman S, Schoorel ENC, Dirksen C, Kwee A, Smits L, de Boer F, et al. SIMPLE: implementation of recommendations from international evidence-based guidelines on caesarean sections in the Netherlands. Protocol for a controlled before and after study. Implement Sci. 2013;8:3. |
| 25. Nair M, Yoshida S, Lambrechts T, Boschi-Pinto C, Bose K, Mason EM, et al. Facilitators and barriers to quality of care in maternal, newborn and child health: a global situational analysis through metareview. BMJ Open. 2014;4(5):e004749. |
| 26. Ndwiga C, Birungi H, Undie C-C, Weyenga H, Sitienei J. Feasibility and effect of integrating tuberculosis screening and detection in postnatal care services: an operations research study. BMC Health Serv Res. 2013;13:99. |
| 27. Tluczek A, Becker T, Laxova A, Grieve A, Racine Gilles CN, Rock MJ, et al. Relationships among health-related quality of life, pulmonary health, and newborn screening for cystic fibrosis. Chest. 2011;140(1):170–7. |
| 28. van Diem MT, Timmer A, Gordijn SJ, Bergman KA, Korteweg FJ, Ravise J, et al. Classification of substandard factors in perinatal care: development and multidisciplinary inter-rater agreement of the Groningen-system. BMC Pregnancy Childbirth. 2015;15(1):215. |
|  |
| Not related to the target phases of the *continuum* |
|  |
| 1. Afsana K, Haque MR, Sobhan S, Shahin SA. BRAC’s experience in scaling-up MNP in Bangladesh. Asia Pac J Clin Nutr. 2014;23(3):377–84. |
| 2. Bautista-Otero A, Garcia-Ubaque JC. [Local government management regarding the quality of children’s healthcare]. Rev salud pública (Bogotá, Colomb. 2012;14 Suppl 2:3–14. |
| 3. Berta P, Seghieri C, Vittadini G. Comparing health outcomes among hospitals: the experience of the Lombardy Region. Health Care Manag Sci. 2013;16(3):245–57. |
| 4. Borkotoky K, Unisa S. Indicators to examine quality of large scale survey data: an example through district level household and facility survey. PLoS One. 2014;9(3):e90113. |
| 5. Coddington J, Sands L, Edwards N, Kirkpatrick J, Chen S. Quality of health care provided at a pediatric nurse-managed clinic. J Am Acad Nurse Pract. 2011;23(12):674–80. |
| 6. Fischer KE. Decision-making in healthcare: a practical application of partial least square path modelling to coverage of newborn screening programmes. BMC Med Inform Decis Mak. 2012;12:83. |
| 7. Flores G, Lin H. Trends in racial/ethnic disparities in medical and oral health, access to care, and use of services in US children: has anything changed over the years? Int J Equity Health. 2013;12:10. |
| 8. Halliday J, Wilson C, Hammarberg K, Doyle LW, Bruinsma F, McLachlan R, et al. Comparing indicators of health and development of singleton young adults conceived with and without assisted reproductive technology. Fertil Steril. 2014;101(4):1055–63. |
| 9. Hersh A. Measures of health-related quality of life in pediatric systemic lupus erythematosus: Childhood Health Assessment Questionnaire (C-HAQ), Child Health Questionnaire (CHQ), Pediatric Quality of Life Inventory Generic Core Module (PedsQL-GC), Pediatric Quali. Arthritis Care Res (Hoboken). 2011;63 Suppl 1:S446-53. |
| 10. Ilboudo PGC, Russell S, D’Exelle B. The long term economic impact of severe obstetric complications for women and their children in Burkina Faso. PLoS One. 2013;8(11):e80010. |
| 11. Johnson NB, Hayes LD, Brown K, Hoo EC, Ethier KA, Centers for Disease Control and Prevention (CDC). CDC National Health Report: leading causes of morbidity and mortality and associated behavioral risk and protective factors--United States, 2005-2013. MMWR Suppl. 2014;31;63(4):3–27. |
| 12. Lugo SE, Pavlicich V. Quality in triage: indicators in patients with respiratory disease. Pediatr Emerg Care. 2013;29(6):710–4. |
| 13. Magill SS, Hellinger W, Cohen J, Kay R, Bailey C, Boland B, et al. Prevalence of healthcare-associated infections in acute care hospitals in Jacksonville, Florida. Infect Control Hosp Epidemiol. 2012;33(3):283–91. |
| 14. Riskin-Mashiah S, Auslender R. Periconceptional folic acid and teratogenic drug use in women undergoing fertility treatments. J Matern Fetal Neonatal Med. 2012;25(10):1899–903. |
| 15. Staniszewska S, Brett J, Redshaw M, Hamilton K, Newburn M, Jones N, et al. The POPPY study: developing a model of family-centred care for neonatal units. Worldviews Evid Based Nurs. 2012;9(4):243–55. |
| 16. Wang CJ, Jonas R, Fu CM, Ng CY, Douglass L. Quality-of-care indicators for infantile spasms. J Child Neurol. 2013;28(1):13–20. |
|  |
| Used from the analyzed repositories or compendiums |
|  |
| 1. Austveg B. Perpetuating power: some reasons why reproductive health has stalled. Reprod Health Matters. 2011;19(38):26–34. |
| 2. Benova L, Macleod D, Footman K, Cavallaro F, Lynch CA, Campbell OMR. Role of the private sector in childbirth care: cross-sectional survey evidence from 57 low- and middle-income countries using Demographic and Health Surveys. Trop Med Int Health. 2015;20(12):1657–73. |
| 3. Boulkedid R, Alberti C, Sibony O. Quality indicator development and implementation in maternity units. Best Pract Res Clin Obstet Gynaecol. 2013;27(4):609–19. |
| 4. Campbell J, Sochas L, Cometto G, Matthews Z. Evidence for action on improving the maternal and newborn health workforce: The basis for quality care. Int J Gynaecol Obstet. 2016;132(1):126–9. |
| 5. de Bernis L, Kinney M V, Stones W, ten Hoope-Bender P, Vivio D, Leisher SH, et al. Stillbirths: ending preventable deaths by 2030. Lancet. 2016;387(10019):703–16. |
| 6. Lenzi J, Luciano L, McDonald KM, Rosa S, Damiani G, Corsello G, et al. Empirical examination of the indicator “pediatric gastroenteritis hospitalization rate” based on administrative hospital data in Italy. Ital J Pediatr. 2014;40:14. |
| 7. Moxon SG, Ruysen H, Kerber KJ, Amouzou A, Fournier S, Grove J, et al. Count every newborn; a measurement improvement roadmap for coverage data. BMC Pregnancy Childbirth. 2015;15 Suppl 2:S8. |
| 8. Pettker CM, Grobman WA. Obstetric Safety and Quality. Obstet Gynecol. 2015;126(1):196–206. |
| 9. Rodin U, Filipović-Grčić B, Đelmiš J, Glivetić T, Juras J, Mustapić Ž, et al. Perinatal Health Statistics as the Basis for Perinatal Quality Assessment in Croatia. Biomed Res Int. 2015;2015:1–9. |
|  |
| Not accessible |
|  |
| 1. Waldrop JB, Anderson CK, Brandon DH. Guideline-based educational intervention to decrease the risk for readmission of newborns with severe hyperbilirubinemia. J Pediatr Health Care. 2013;27(1):41–50. |
|  |
| Other language |
|  |
| 1. Moosavisadat SM, Lamyian M, Parsap S, Hajizadeh E. Comparison of maternity care quality in teaching and non-teaching hospitals in Khorram Abad, Islamic Republic of Iran. East Mediterr Heal J = La Rev santé la Méditerranée Orient = al-Majallah al-ṣiḥḥīyah li-sharq al-mutawassiṭ. 2011;17(8):638–45. |
| 2. Rudasingwa M, Soeters R, Bossuyt M. The effect of performance-based financial incentives on improving health care provision in Burundi: a controlled cohort study. Glob J Health Sci. 2015;7(3):15–29. |
| 3. Voerman GE, Calsbeek H, Maassen ITHM, Wiegers TA, Braspenning J. A systematic approach towards the development of a set of quality indicators for public reporting in community-based maternity care. Midwifery. 2013;29(4):316–24. |
|  |
| Duplicated |
|  |
| 1. Luitjes S, Wouters M, Franx A, Bolte A, de Groot C, van Tulder M, et al. PP095. Guideline-based development of quality indicators for hypertensive diseases in pregnancy. Pregnancy Hypertens An Int J Women’s Cardiovasc Heal. 2012;2(3):291–2. |
|  |
| **Full text articles rewiew** |
|  |
| 1. Albouy-Llaty M, Nadeau C, Descombes E, Pierre F, Migeot V. Improving perinatal Group B streptococcus screening with process indicators. J Eval Clin Pract. 2012;18(4):727–33. |
| 2. Aluvaala J, Nyamai R, Were F, Wasunna A, Kosgei R, Karumbi J, et al. Assessment of neonatal care in clinical training facilities in Kenya. Arch Dis Child. 2015;100(1):42–7. |
| 3. Armstrong CE, Magoma M, Ronsmans C. Magnitude of maternal and neonatal mortality in Tanzania: A systematic review. Int J Gynaecol Obstet. 2015;130(1):98–110. |
| 4. Awoonor-Williams JK, Bawah AA, Nyonator FK, Asuru R, Oduro A, Ofosu A, et al. The Ghana essential health interventions program: a plausibility trial of the impact of health systems strengthening on maternal & child survival. BMC Health Serv Res. 2013;13 Suppl 2:S3. |
| 5. Bagriansky J, Champa N, Pak K, Whitney S, Laillou A. The economic consequences of malnutrition in Cambodia, more than 400 million US dollar lost annually. Asia Pac J Clin Nutr. 2014;23(4):524–31. |
| 6. Barker L, Field D. Neonatal quality measures: time to show developmental progress? Arch Dis Child Fetal Neonatal Ed. 2014;99(6):F505-9. |
| 7. Bassani DG, Arora P, Wazny K, Gaffey MF, Lenters L, Bhutta ZA. Financial incentives and coverage of child health interventions: a systematic review and meta-analysis. BMC Public Health. 2013;13 Suppl 3:S30. |
| 8. Binyaruka P, Patouillard E, Powell-Jackson T, Greco G, Maestad O, Borghi J. Effect of Paying for Performance on Utilisation, Quality, and User Costs of Health Services in Tanzania: A Controlled Before and After Study. PLoS One. 2015;10(8):e0135013. |
| 9. Bollini P, Quack-Lötscher K. Guidelines-based indicators to measure quality of antenatal care. J Eval Clin Pract. 2013;19(6):1060–6. |
| 10. Bonfill X, Roqué M, Aller MB, Osorio D, Foradada C, Vives A, et al. Development of quality of care indicators from systematic reviews: the case of hospital delivery. Implement Sci. 2013;8:42. |
| 11. Boulkedid R, Sibony O, Goffinet F, Fauconnier A, Branger B, Alberti C. Quality indicators for continuous monitoring to improve maternal and infant health in maternity departments: a modified Delphi survey of an international multidisciplinary panel. PLoS One. 2013;8(4):e60663. |
| 12. Brenner S, De Allegri M, Gabrysch S, Chinkhumba J, Sarker M, Muula AS. The quality of clinical maternal and neonatal healthcare - a strategy for identifying “routine care signal functions”. PLoS One. 2015;10(4):e0123968. |
| 13. Bryce J, Arnold F, Blanc A, Hancioglu A, Newby H, Requejo J, et al. Measuring coverage in MNCH: new findings, new strategies, and recommendations for action. PLoS Med. 2013;10(5):e1001423. |
| 14. Centers for Disease Control and Prevention (CDC). Vital signs: hospital practices to support breastfeeding--United States, 2007 and 2009. MMWR Morb Mortal Wkly Rep. 2011;60(30):1020–5. |
| 15. Cooper BP, Scharff DP, Elliott M, Rotter B. The impact of SLHS program on perinatal indicators. Matern Child Health J. 2013;17(6):1158–65. |
| 16. de Oliveira RLA, da Fonseca CRB, Carvalhaes MA de BL, Parada CMG de L. Evaluation of pre-natal care from the perspective of different models in primary care. Rev Lat Am Enfermagem. 2013;21(2):546–53. |
| 17. Dunlop AL, McCarthy BJ, Freymann GR, Smith CK, Bugg GW, Brann AW. Analysis of feto-infant mortality using the BABIES framework: Georgia 1981-83 through 2001-03. Int J Heal Res. 2010;3(3):153–63. |
| 18. Dunn S, Sprague AE, Fell DB, Dy J, Harrold J, Lamontagne B, et al. The use of a quality indicator to reduce elective repeat Caesarean section for low-risk women before 39 weeks’ gestation: the Eastern Ontario experience. J Obstet Gynaecol Canada  JOGC = J d’obstétrique gynécologie du Canada  JOGC. 2013;35(4):306–16. |
| 19. Edwards RA, Dee D, Umer A, Perrine CG, Shealy KR, Grummer-Strawn LM. Using benchmarking techniques and the 2011 maternity practices infant nutrition and care (mPINC) survey to improve performance among peer groups across the United States. J Hum Lact. 2014;30(1):31–40. |
| 20. Eriksson J, Baker T, Jornvall H, Irestedt L, Mulungu M, Larsson E. Quality of anaesthesia for Caesarean sections: a cross-sectional study of a university hospital in a low-income country. Trop Med Int Health. 2015;20(10):1329–36. |
| 21. Escuriet R, Pueyo MJ, Perez-Botella M, Espada X, Salgado I, Gomez A, et al. Cross-sectional study comparing public and private hospitals in Catalonia: is the practice of routine episiotomy changing? BMC Health Serv Res. 2015;15:95. |
| 22. Esquivel M, Álvarez G, Izquierdo ME, Martínez D, Tamayo V. Well child care: a comprehensive strategy for Cuban children and adolescents. MEDICC Rev. 2014;16(1):7–11. |
| 23. Feng XL, Theodoratou E, Liu L, Chan KY, Hipgrave D, Scherpbier R, et al. Social, economic, political and health system and program determinants of child mortality reduction in China between 1990 and 2006: A systematic analysis. J Glob Health. 2012;2(1):10405. |
| 24. Fischer Walker CL, Fontaine O, Black RE. Measuring coverage in MNCH: current indicators for measuring coverage of diarrhea treatment interventions and opportunities for improvement. PLoS Med. 2013;10(5):e1001385. |
| 25. Foster J, Gossett S, Burgos R, Caceres R, Tejada C, Dominguez Garcia L, et al. Improving maternity care in the Dominican Republic: a pilot study of a community-based participatory research action plan by an international healthcare team. J Transcult Nurs. 2015;26(3):254–60. |
| 26. Ganaba R, Ilboudo PGC, Cresswell JA, Yaogo M, Diallo CO, Richard F, et al. The obstetric care subsidy policy in Burkina Faso: what are the effects after five years of implementation? Findings of a complex evaluation. BMC Pregnancy Childbirth. 2016;16(1):84. |
| 27. Gimbel S, Voss J, Rustagi A, Mercer MA, Zierler B, Gloyd S, et al. What does high and low have to do with it? Performance classification to identify health system factors associated with effective prevention of mother-to-child transmission of HIV delivery in Mozambique. J Int AIDS Soc. 2014;17:18828. |
| 28. Giménez Roca C, Martínez Sánchez L, Calzada Baños Y, Trenchs Sainz de la Maza V, Quintilla Martínez JM, Luaces Cubells C. [Assessment of quality indicators in pediatric poisoning in an emergency service]. An Pediatr (Barcelona, Spain  2003). 2014;80(1):34–40. |
| 29. Gupta M, Angeli F, van Schayck OCP, Bosma H. Effectiveness of a multiple-strategy community intervention to reduce maternal and child health inequalities in Haryana, North India: a mixed-methods study protocol. Glob Health Action. 2015;8:25987. |
| 30. Hajeebhoy N, Nguyen PH, Tran DT, de Onis M. Introducing infant and young child feeding indicators into national nutrition surveillance systems: lessons from Vietnam. Matern Child Nutr. 2013;9 Suppl 2:131–49. |
| 31. Hill K, You D, Inoue M, Oestergaard MZ, Technical Advisory Group of United Nations Inter-agency Group for Child Mortality Estimation. Child mortality estimation: accelerated progress in reducing global child mortality, 1990-2010. PLoS Med. 2012;9(8):e1001303. |
| 32. Hodgins S, D’Agostino A. The quality-coverage gap in antenatal care: toward better measurement of effective coverage. Glob Heal Sci Pract. 2014;2(2):173–81. |
| 33. Huda FA, Ahmed A, Ford ER, Johnston HB. Strengthening health systems capacity to monitor and evaluate programmes targeted at reducing abortion-related maternal mortality in Jessore district, Bangladesh. BMC Health Serv Res. 2015;15:426. |
| 34. Jones AD, Ickes SB, Smith LE, Mbuya MNN, Chasekwa B, Heidkamp RA, et al. World Health Organization infant and young child feeding indicators and their associations with child anthropometry: a synthesis of recent findings. Matern Child Nutr. 2014;10(1):1–17. |
| 35. Kanyuka M, Ndawala J, Mleme T, Chisesa L, Makwemba M, Amouzou A, et al. Malawi and Millennium Development Goal 4: a Countdown to 2015 country case study. Lancet Glob Heal. 2016;4(3):e201-14. |
| 36. Kayode GA, Amoakoh-Coleman M, Brown-Davies C, Grobbee DE, Agyepong IA, Ansah E, et al. Quantifying the validity of routine neonatal healthcare data in the Greater Accra Region, Ghana. PLoS One. 2014;9(8):e104053. |
| 37. Kildea S, Gao Y, Rolfe M, Josif CM, Bar-Zeev SJ, Steenkamp M, et al. Remote links: Redesigning maternity care for Aboriginal women from remote communities in Northern Australia - A comparative cohort study. Midwifery. 2016;34:47–57. |
| 38. Kowalkowski M, Gould JB, Bose C, Petersen LA, Profit J. Do practicing clinicians agree with expert ratings of neonatal intensive care unit quality measures? J Perinatol. 2012;32(4):247–52. |
| 39. Luitjes SH, Wouters MG, Franx A, Bolte AC, de Groot CJ, van Tulder MW, et al. Guideline-based development of quality indicators for hypertensive diseases in pregnancy. Hypertens pregnancy. 2013;32(1):20–31. |
| 40. Marchant T, Schellenberg J, Peterson S, Manzi F, Waiswa P, Hanson C, et al. The use of continuous surveys to generate and continuously report high quality timely maternal and newborn health data at the district level in Tanzania and Uganda. Implement Sci. 2014;9:112. |
| 41. Meier PP, Patel AL, Bigger HR, Rossman B, Engstrom JL. Supporting breastfeeding in the neonatal intensive care unit: Rush Mother’s Milk Club as a case study of evidence-based care. Pediatr Clin North Am. 2013;60(1):209–26. |
| 42. Moran AC, Kerber K, Sitrin D, Guenther T, Morrissey CS, Newby H, et al. Measuring coverage in MNCH: indicators for global tracking of newborn care. PLoS Med. 2013;10(5):e1001415. |
| 43. Murray C, Newby H. Data resource profile: United Nations Children’s Fund (UNICEF). Int J Epidemiol. 2012;41(6):1595–601. |
| 44. Nelissen EJT, Mduma E, Ersdal HL, Evjen-Olsen B, van Roosmalen JJM, Stekelenburg J. Maternal near miss and mortality in a rural referral hospital in northern Tanzania: a cross-sectional study. BMC Pregnancy Childbirth. 2013;13:141. |
| 45. Otsea K, Benson J, Alemayehu T, Pearson E, Healy J. Testing the Safe Abortion Care model in Ethiopia to monitor service availability, use, and quality. Int J Gynaecol Obstet. 2011;115(3):316–21. |
| 46. Owens L, Semrau K, Mbewe R, Musokotwane K, Grogan C, Maine D, et al. The state of routine and emergency obstetric and neonatal care in Southern Province, Zambia. Int J Gynecol Obstet. 2015;128(1):53–7. |
| 47. Pereira C, Mbaruku G, Nzabuhakwa C, Bergström S, McCord C. Emergency obstetric surgery by non-physician clinicians in Tanzania. Int J Gynaecol Obstet. 2011;114(2):180–3. |
| 48. Profit J, Gould JB, Zupancic JAF, Stark AR, Wall KM, Kowalkowski MA, et al. Formal selection of measures for a composite index of NICU quality of care: Baby-MONITOR. J Perinatol. 2011;31(11):702–10. |
| 49. Quinzanos I, Davis L, Keniston A, Nash A, Yazdany J, Fransen R, et al. Application and feasibility of systemic lupus erythematosus reproductive health care quality indicators at a public urban rheumatology clinic. Lupus. 2015;24(2):203–9. |
| 50. Rabbani F, Pradhan NA, Zaidi S, Azam SI, Yousuf F. Service quality in contracted facilities. Int J Health Care Qual Assur. 2015;28(5):520–31. |
| 51. Renzi C, Sorge C, Fusco D, Agabiti N, Davoli M, Perucci CA. Reporting of quality indicators and improvement in hospital performance: the P.Re.Val.E. Regional Outcome Evaluation Program. Health Serv Res. 2012;47(5):1880–901. |
| 52. Requejo JH, Newby H, Bryce J. Measuring coverage in MNCH: challenges and opportunities in the selection of coverage indicators for global monitoring. PLoS Med. 2013;10(5):e1001416. |
| 53. Requejo J, Victora C, Bryce J, Scientific Review Group of Countdown to 2015. Data resource profile: countdown to 2015: maternal, newborn and child survival. Int J Epidemiol. 2014;43(2):586–96. |
| 54. Rowe AK, Onikpo F, Lama M, Osterholt DM, Deming MS. Impact of a malaria-control project in Benin that included the integrated management of childhood illness strategy. Am J Public Health. 2011;101(12):2333–41. |
| 55. Santos J V, Correia C, Cabral F, Bernardes J, Costa-Pereira A, Freitas A. Should European perinatal indicators be revisited? Eur J Obstet Gynecol Reprod Biol. 2013;170(1):85–9. |
| 56. Sibanda T, Fox R, Draycott TJ, Mahmood T, Richmond D, Simms RA. Intrapartum care quality indicators: a systematic approach for achieving consensus. Eur J Obstet Gynecol Reprod Biol. 2013;166(1):23–9. |
| 57. Smit M, Chan K-LL, Middeldorp JM, van Roosmalen J. Postpartum haemorrhage in midwifery care in the Netherlands: validation of quality indicators for midwifery guidelines. BMC Pregnancy Childbirth. 2014;14:397. |
| 58. Smit M, Sindram SIC, Woiski M, Middeldorp JM, van Roosmalen J. The development of quality indicators for the prevention and management of postpartum haemorrhage in primary midwifery care in the Netherlands. BMC Pregnancy Childbirth. 2013;13:194. |
| 59. Souza JP, Gulmezoglu AM, Carroli G, Lumbiganon P, Qureshi Z. The world health organization multicountry survey on maternal and newborn health: study protocol. BMC Health Serv Res. 2011;11:286. |
| 60. Sprague AE, Dunn SI, Fell DB, Harrold J, Walker MC, Kelly S, et al. Measuring quality in maternal-newborn care: developing a clinical dashboard. J Obstet Gynaecol Canada  JOGC = J d’obstétrique gynécologie du Canada  JOGC. 2013;35(1):29–38. |
| 61. Steenkamp M, Rumbold AR, Kildea S, Bar-Zeev SJ, Kruske S, Dunbar T, et al. Measuring what matters in delivering services to remote-dwelling Indigenous mothers and infants in the Northern Territory, Australia. Aust J Rural Health. 2012;20(4):228–37. |
| 62. Stienen JJC, Tabbers MM, Benninga MA, Harmsen M, Ouwens MMTJ. Development of quality indicators based on a multidisciplinary, evidence-based guideline on pediatric constipation. Eur J Pediatr. 2011;170(12):1513–9. |
| 63. Stivanello E, Rucci P, Carretta E, Pieri G, Seghieri C, Nuti S, et al. Risk adjustment for inter-hospital comparison of caesarean delivery rates in low-risk deliveries. PLoS One. 2011;6(11):e28060. |
| 64. Sychareun V, Hansana V, Phengsavanh A, Chaleunvong K, Eunyoung K, Durham J. Data verification at health centers and district health offices in Xiengkhouang and Houaphanh Provinces, Lao PDR. BMC Health Serv Res. 2014;14:255. |
| 65. Woiski MD, Scheepers HC, Liefers J, Lance M, Middeldorp JM, Lotgering FK, et al. Guideline-based development of quality indicators for prevention and management of postpartum hemorrhage. Acta Obstet Gynecol Scand. 2015;94(10):1118–27. |
| 66. Zhang Y, Chen L, van Velthoven MHMMT, Wang W, Liu L, Du X, et al. mHealth Series: Measuring maternal newborn and child health coverage by text messaging – a county–level model for China. J Glob Health. 2013;3(2):20402. |
| 67. Zimba E, Kinney M V, Kachale F, Waltensperger KZ, Blencowe H, Colbourn T, et al. Newborn survival in Malawi: a decade of change and future implications. Health Policy Plan. 2012;27 Suppl 3:iii88-103. |
|  |
| **Gray literature** |
| 1. UNICEF: Fulfilling the Health Agenda for Women and Children. Countdown to 2015. UNICEF and World Health Organization. 2014, Geneva, Switzerland. |
| 2. Euro-Peristat. Euro-Peristat list of indicator, updated 2012. <http://www.europeristat.com/images/doc/updated%20indicator%20list.pdf>. Accessed 7 May 2016 |
| 3. International Federation of Red Cross and Red Crescent Societies. Maternal, newborn and child health framework. 2013. http://www.ifrc.org/PageFiles/93927/1232600-MNCH Framework report_LR (2).pdf. Accessed 26 May 2016 |
| 4. Rutstein SO, Rojas G. Guide to DHS statistics. Demographic and health surveys methodology. 2006. <http://www.measuredhs.com/pubs/pdf/DHSG1/Guide_to_DHS_Statistics_29Oct2012_DHSG1.pdf>. Accessed 23 Jul 2016. |
| 5. UNICEF. Estado Mundial de la Infancia 2009. New York, NY; 2008. |
| 6. Safe Motherhood and Reproductive Health Working Group and CORE Group. Maternal and Newborn Standards and Indicators Compendium. Washington, D.C; 2004. <https://www.mchip.net/sites/default/files/Maternal_and_Newborn_Standards_and_Indicators_Compendium_2004.pdf>. Accessed 26 May 2016. |
| 7. Countdown to 2015, Health Metrics Network, UNICEF, World Health Organization. Monitoring maternal, newborn and child health: understanding key progress indicators. Geneva: World Health Organization; 2011. |
| 8. World Health Organization. Consultation on improving measurement of the quality of maternal, newborn and child care in health facilities. Geneva: World Health Organization; 2014.  9. World Health Organization, UNICEF. EVERY NEWBORN ACTION PLAN Country. 2015.  10. World Health Organization, UNICEF, Committing to Child Survival. Every Newborn Progress Report. Geneva: World Health Organization; 2014.  11. World Health Organization. Every newborn: an action plan to end preventable deaths. 2014. <http://www.who.int/iris/handle/10665/127938>. Accessed 26 Jul 2018.  12. World Health Organization. Global Reference List of 100 Core Health Indicators. Geneva; 2015. <http://apps.who.int/iris/bitstream/handle/10665/173589/WHO_HIS_HSI_2015.3_eng.pdf?sequence=1>. Accessed 9 Jun 2016.  13. World Health Organization. Standards for improving quality of maternal and newborn care in health facilities. Geneva: World Health Organization; 2016.  14. Every Woman Every Child. Indicator and monitoring framework for the global strategy for women’s, children’s, and adolescents’ health (2016–2030). Geneva: World Health Organization; 2016. |
| * Duplicated |
