# Supplementary material for: Indicators for monitoring maternal and neonatal quality care: a systematic review
Source: BMC Pregnancy Childbirth. 2019 Jan 11;19:25. doi: 10.1186/s12884-019-2173-2 (PMC6330388; doi:10.1186/s12884-019-2173-2)
Supplement: Supplementary file 3 — Description 87 indicators. 87 indicators selected in English and Spanish ordered by each phase of the continuum with reference. (DOCX 112 kb) [file 12884_2019_2173_MOESM3_ESM.docx]

**Additional file 3a.** Description and characteristics of the selected indicators in English

| **Phase of the continuum** | **Indicator** | **Numerator** | **Denominator** | **Definitions** | **Type indicator** | **Level of care** | **Organizational level of application** | **Income level of the country** | **Reference #** |
| --- | --- | --- | --- | --- | --- | --- | --- | --- | --- |
| **Pregnancy** | Frequency of appropriate Prenatal Care | Women who had the appropriate number of expected prenatal visits | Deliveries in a year | Percentage of deliveries that had the following number of expected prenatal visits: less than 21% of expected visits; 21%–40% of expected visits; 41 %–60 % of expected visits; 61 %–80 % of expected visits; greater than or equal to 81 % of expected visits.  Exclude non-live births | Process | 1 | 3 | 2 | 1 |
|  | Proportion of women who receive antenatal assessments by 13 weeks of pregnancy | Number in the denominator seen for booking by 13 weeks 0 days | The number of pregnant women | -- | Process | 1 | 3 | 2 | 2 |
|  | Proportion of women with eclampsia treated with magnesium sulphate | Women with eclampsia who received magnesium sulphate | Women with eclampsia | ICD-9: 642.6.  Exclusion: contraindication to magnesium sulphate | Process | 2 | 2 | 3 | 3 |
|  | Proportion of women with severe pre-eclampsia who were treated with magnesium sulphate | Women with severe pre-eclamsia who received magnesium sulphate | Women with severe pre-eclampsia | ICD-9: 642.5  Exclusion: contraindication to magnesium sulphate | Process | 2 | 2 | 3 | 3 |
|  | Proportion of women with singleton pregnancies and threatened preterm labour who receive corticosteroids | Women who received corticoids | Singleton pregnancies between 26-34 weeks | ICD-9: 644.03, 644.10, 644.13, 644.20, 644.21. Exclusion: corticoids contraindications | Process | 2 | 2 | 3 | 3 |
|  | Proportion of women with threatened preterm labour (TPL) treated with magnesium sulphate | Women who received magnesium sulphate | Women who received pharmacological treatment for TPL | ICD-9: 644.03, 644.10,  644.13, 644.20,  644.21 | Process | 2 | 2 | 3 | 3 |
|  | Proportion of women who are treated with calcium channel blockers (CCB) for inhibiting preterm labour | Women who received CCB | Pregnancies between 22-34 weeks | ICD-9: : 644.03, 644.10, 644.13, 644.20, 644.21  Exclusion: contraindication to CCB. | Process | 2 | 2 | 3 | 3 |
|  | Percent of women with night blindness in last pregnancy | Number of women who had night blindness during last pregnancy | Number of women with last pregnancy resulting in live birth during past 3 years | Expressed as a percentage.  Maternal night blindness is the inability to see normally after dusk or at night during pregnancy, especially the last trimester and early postpartum.  Need to exclude women whose blindness was probably due to visual impairment from other causes. | Outcome | 1 | 3 | 3 | 24 |
| **Childbirth** | Appropriate prophylactic antibiotic received within one hour prior to surgical incision – cesarean section | Percentage of women who receive recommended antibiotics within one hour before the start of cesarean section | All patients undergoing cesarean section without evidence of prior infection or already receiving prophylactic antibiotics for other reasons | Exclusions: Women with evidence of prior infection or already receiving prophylactic antibiotics for other reasons; or with significant allergies to penicillin and/or cephalosporin AND allergies to gentamicin and/or clindamycin | Process | 2 | 2 | 2 | 5 |
|  | Intrapartum antibiotic prophylaxis for group B streptococcus (GBS) | All eligible patients who receive intrapartum antibiotic prophylaxis for GBS | All women delivering live infants, except certain classes who are specifically deemed not to be at risk of vertical transmission of GBS | Excluded populations (not at risk): 1)Patient screened negative for GBS at 35-37 weeks of delivery;2) patients delivering via planned cesarean sections (in the absence of labor or amniotic membrane rupture); 3)patients already on antibiotics for a prenatal maternal infection or other  Prophylaxis; 4) deliveries resulting in stillbirths (ICD-9- V.27.1, V27.3, V27.4, V27.6 or V27.7) | Process | 2 | 2 | 2 | 6 |
|  | Appropriate deep venous thrombosis prophylaxis in women undergoing cesarean delivery | Number of women undergoing cesarean delivery receiving either pneumatic compression device or medical prophylaxis prior to cesarean delivery | All women undergoing cesarean delivery | HCPCS codes: J1644, J1650, J1645, J1655 | Process | 2 | 2 | 2 | 4 |
|  | Proportion of women undergoing caesarean section who receive antibiotic therapy | Women who received antibiotics | Women undergoing caesarean section | ICD-9: 74.XX. The value “X” means any number between 0 to 9.  Antibiotic therapy includes all classes of antibiotics (co-amoxiclav is not recommended) | Process | 2 | 2 | 3 | 3 |
|  | Proportion of women with preterm rupture of membranes (PRM) who receive antibiotic treatment | Women who received antibiotics | Pregnancies between 22-34 weeks with PRM | ICD-9: 658.10, 658.11  Antibiotic treatment includes all classes of antibiotics, co-amoxiclav is not recommended | Process | 2 | 2 | 3 | 3 |
|  | Proportion of women who are administered uterotonics in the third stage of labour | Women who received uterotonics in the third stage of labour | Women in labour | ICD-9: 641.X1, 642.X1,  676.X1. The value “X” means any number between 0 to 9.  Any uterotonic drug that can be used, e.g. oxytocin (intravenous (IV) or intramuscular (IM)); syntometrine (IM); ergometrine (IV or IM); misoprostol (IM).  Exclusion: contraindication to uterotonics and patient refusal to receive uterotonic. | Process | 2 | 2 | 3 | 3 |
|  | Proportion of women delivering at term who had group B streptococcus (GBS)screening at 35 to 37 weeks’ gestation | Number of women in labor who deliver at term and have GBS screening at 35 to 37 weeks’ gestation | Number of women delivering at term | Expressed as a percentage  Delivering at term: ≥37 weeks | Process | 2 | 2 | 2 | 9 |
|  | Proportion of women with term pregnancies and a breech presentation in which external cephalic version is performed or offered | Women in whom cephalic version was performed or offered | Breech presentation | ICD-9: 73.91 | Process | 2 | 2 | 3 | 3 |
|  | Proportion of women induced with an indication of post-dates who are at less than 41 weeks’ gestation at delivery | Number of women who were induced with an indication of induction labour of post-dates with at less than 41 weeks of gestation at delivery | Number of women who who were induced with an indication for induction of labour of post-date | Expressed as a percentage  Post-dates: ≥41 weeks gestation | Process | 2 | 2 | 2 | 9 |
|  | Proportion of women with labour induction who give birth after 41 weeks of gestation | Women with labour induction | Women with $\geq$41weeks of pregnancy | ICD-9: 641.X1, 642.X1,  676.X1. The value “X” means any number between 0 to 9.  Exclusion: Spontaneous labour, non-urgent caesarean delivery  Labor induction: process  of artificially stimulating the uterus to start labour | Process | 2 | 2 | 3 | 3 |
|  | Proportion of women whose second-degree perineal tear or episiotomy is repaired with continuous suture | Women in whom continuous suture was performed | Women with second-degree perineal tear or episiotomy | ICD-9: 664.10, 664.11, 644.14, 73.6. | Process | 2 | 2 | 3 | 3 |
|  | Proportion of pregnant women having a planned caesarean section who have the procedure carried out at or after 39 weeks 0 days | The number in the denominator who had a caesarean section at or after 39 weeks 0 days | Number of women who had a planned caesarean section without maternal or fetal indication for an early birth | Maternal or fetal indications includes but is not limited to the following: significant conditions, hypertensive disease, diabetes or gestational diabetes, significant antepartum haemorrhage, intrauterine growth restriction, congenital abnormality, hydrops or compromise due to blood group incompatibility, acute fetal compromise, multiple pregnancy | Process | 2 | 2 | 2 | 2 |
|  | Rate of repeat caesarean section in low-risk women prior to 39 weeks’ gestation | Number of low-risk women with a caesarean section performed before 39 weeks’ gestation | Number of low-risk women who had a repeat caesarean section at term (≥37 weeks) | Expressed as a percentage  Repeat caesarean section: women with a history of one or more previous caesarean sections. For this analysis, the definition includes women with a singleton live birth, between 37 and 42 weeks of gestational age, with no maternal medical problems, no obstetrical complications, and none of the following indications for the Caesarean section: cord prolapse, fetal anomaly, intrauterine growth restriction/small for gestational age, large for gestational age, nonreassuring fetal status, placenta previa, placental abruption, pre-eclampsia, and preterm rupture of membranes | Process | 3 | 2 | 2 | 9 |
|  | Proportion of unjustified episiotomies | Episiotomy without any reason documented | Women in who episiotomy was performed | ICD-9: 73.6 | Process | 2 | 2 | 3 | 3 |
|  | Proportion of women having perineal shaving on admission to the delivery room | Women for whom perineal shaving was performed | Women in labour | ICD-9: 641.X1, 642.X1, 676.X1. The value “X” means any number between 0 to 9 | Process | 2 | 2 | 3 | 3 |
|  | Proportion of women whose peritoneum is sutured at caesarean delivery | Women for who peritoneum was sutured | Women who received caesarean | ICD-9: 74.XX. The value “X” means any number between 0 to 9 | Process | 2 | 2 | 3 | 3 |
|  | Proportion of women who are given an enema during labour | Women who were given an enema | Women in labour | ICD-9: 641.X1, 642.X1, 676.X1. The value “X” means any number between 0 to 9 | Process | 2 | 2 | 3 | 3 |
|  | Rate of uterine rupture | Number of women with uterine rupture | Number of women delivered | -- | Outcome | 2 | 1 | 2 | 18 |
|  | Proportion of women with prolonged labour | Number of women who have not given birth or were not transferred out within 12 hours of active labour | Number of women in active labour in the health facility | Active labour: cervical dilatation ≥ 4 cm (documented, with clear documentation of delivery time) | Outcome | 3 | 2 | 3 | 14 |
|  | Births without obstetric intervention | Number of deliveries without selected obstetric interventions | Deliveries resulting in a live birth or stillbirth | Births without obstetric intervention are defined as births to women whose labour starts spontaneously, progresses spontaneously without drugs, and who give birth spontaneously. Selected interventions : women who experience any one or more of the following: induction of labour (with prostaglandins, oxytocic’s or ARM); epidural or spinal or general anaesthetic; forceps or ventouse; caesarean section; episiotomy | Outcome | 2 | 3 | 3 | 22 |
|  | Instrumental vaginal delivery rate | Number of neonates delivered by instrumental extraction extractor) | Number of vaginal births (all live born neonates including those with birth defects) | Instrumental extraction: using obstetric forceps or vacuum extractor | Outcome | 2 | 1 | 2 | 18 |
|  | Caesarean section before labour | Number of caesarean sections before labour | Number of women delivered | -- | Outcome | 2 | 1 | 2 | 18 |
|  | Caesarean section during labour | Number of caesarean sections during labour | Number of women delivered | -- | Outcome | 2 | 1 | 2 | 18 |
|  | Episiotomy rate | Number of women who had an episiotomy | All women who had a vaginal delivery | Expressed as a proportion  Presented for all vaginal deliveries and then for non-instrumental and instrumental separately | Outcome | 2 | 3 | 3 | 13 |
|  | Incidence of tear of the perineum | Number of women who had a first, second, third, or fourth-degree tear of the perineum | Women who had a vaginal delivery | Expressed as a proportion  Presented for all vaginal deliveries and then for non-instrumental and instrumental separately.  Categories as follow (ICD-10): 1st degree tear (O70.0) involves the fourchette, perineal skin and vaginal membrane; 2nd degree tear (O70.1) includes the skin and mucous membrane, and the fascia and muscles of the perineal body; 3rd degree tear (O70.2) extends through skin, mucous membrane and perineal body and involves the muscle of the anal sphincter and; 4th degree tear (O70.3) extends through the rectal mucosa to expose the lumen of the rectum. | Outcome | 2 | 3 | 3 | 13 |
|  | Maternal Intensive Care Unit (ICU) transfer and/or admission | Number of women transferred and/or admitted to the ICU | Number of women delivered | -- | Outcome | 2 | 1 | 2 | 18 |
|  | Intrapartum stillbirth rate | Number of stillborn infants weighing > 1000 g and fetal heart rate documented on admission | Number of births of infants weighing > 1000 g in facility | -- | Outcome | 3 | 2 | 3 | 14 |
|  | Proportion of women treated for obstetric complications that are abortion related | Number of women with abortion complications | Number of women with obstetric complications treated at facility in a given period | Obstetric complications that are abortion related: incomplete abortion, failed abortion, haemorrhage, infection, uterine perforation | Outcome | 3 | 2 | 1 | 17 |
|  | Proportion of women treated for abortion complications that are serious | Number of women with serious abortion complications | Number of women with all abortion complications treated at facility in a given period | Abortion complications: incomplete abortion, failed abortion, haemorrhage, infection, uterine perforation | Outcome | 3 | 2 | 1 | 17 |
| **Puerperium** | Proportion of Rh-negative women who are given Anti-D within 72 hours after the birth of a Rh-positive or Rh-undetermined baby | Women who received Anti-D | Rh-negative women with Rh-positive or Rh-undetermined newborn | Exclusion: Women with prior Rh sensitization | Process | 2 | 2 | 3 | 3 |
|  | Proportion of women with severe systemic infection or sepsis in postnatal period, including readmissions | Number of women seen in the facility with severe systemic infection or sepsis in postnatal period, including readmissions after birth in facility | Number of women giving birth in the health facility | Postnatal period: first 42 days after birth | Outcome | 2 | 2 | 3 | 14 |
| **Newborn care** | Hearing screening prior to hospital discharge | Live birth encounters during the measurement period where a patient born at the facility is screened for hearing loss prior to discharge or not screened due to medical reasons | Live birth encounters at a hospital or birthing facility where the newborn was discharged with hospital stays ≤120 days during the measurement period | Denominator exclusions:  Live birth encounters where the patient expires prior to discharge and has not received hearing screening for the left or right ear | Process | 2 | 2 | 2 | 10 |
|  | Hepatitis B vaccine coverage among all live newborn infants prior to hospital or birthing facility discharge | Number of live newborn administered hepatitis B vaccine prior to discharge from the hospital/birthing facility ("birth dose" of hepatitis B vaccine) | Number of live newborn infants born at the hospital/birthing facility | The numerator includes the ICD-10 codes: Z37.0 ,Z37.2, Z37.3, Z37.50, Z37.51, Z37.52, Z37.53, Z37.54, Z37.59, Z37.60, Z37.61, Z37.62, Z37.63, Z37.64, Z37.69, Z38.00, Z38.001, Z38.1, Z38.2, Z38.30 , Z38.31 , Z38.4, Z38.5, Z38.61 ,Z38.62 ,Z38.63, Z38.64, Z38.65, Z38.66, Z38.68, Z38.69, Z38.7, Z38.8.  The denominator excludes: the number of infants born at the facility during one calendar year, whose  parent/guardian refused administration of a birth dose of hepatitis B vaccine before discharge from the  hospital/birthing facility (ICD-9 code V64.05 convert to ICD-10 code Z28.82)  Birth Dose: hepatitis B vaccine before hospital discharge (or by 1 month of age if not yet discharged) | Process | 2 | 2 | 2 | 7 |
|  | Rate of formula supplementation in term infants whose mothers intended to breastfeed | Number of term live babies receiving formula supplementation | Number of term babies whose mothers intended to breastfeed | Expressed as a percentage | Process | 2 | 2 | 2 | 9 |
|  | Proportion of babies exclusively or partially breastfed 6-8 weeks after birth | Number in the denominator recorded as being exclusively or partially breast-fed after 6 to 8 weeks | Number of infants due a six to eight week check | -- | Process | 1 | 3 | 2 | 2 |
|  | Very low birth weight rate | Number of infants with birth weight 500-1499 grams | Number of births (live births and stillbirths) | -- | Outcome | 3 | 3 | 2 | 16 |
|  | Healthy Term Newborn | Newborns with absence of conditions or procedures reflecting morbidity that happened during birth and nursery care to an otherwise normal infant | All singleton, term (≥37 weeks), inborn, livebirths in their birth admission | The denominator further has eliminated fetal conditions likely to be present before labor. Maternal and obstetrical conditions (e.g. hypertension, prior cesarean, malpresentation) are not excluded unless evidence of fetal effect prior to labor | Outcome | 1 | 1 | 2 | 23 |
|  | Birth ≥ 37 weeks with Apgar <7 at 5 min | Number of births ≥ 37 weeks with Apgar <7 at 5 min | Total number of births ≥ 37 weeks | -- | Outcome | 2 | 1 | 2 | 18 |
|  | Percentage of high-risk newborns with staphylococcal and gram negative septicemias or bacteremias | Newborns with septicemia or bacteremia or sepsis with a bloodstream infection confirmed | Liveborn newborns with birth weight ≤1500 g | NUMERADOR inclusions: 1) 1.ICD-10-CM Other Diagnosis Codes for newborn septicemia or bacteremia (P36.0, P36.10, P36.19, P36.2, P36.30, P36.39, P36.4, P36.5, P36.8, P36.9, R78.81)  2.ICD-10-CM Other Diagnosis Codes for sepsis (A02.1, A22.7, A26.7, A32.7, A40.0, A40.1, A40.3, A40.8, A40.9, A41.01, A41.02, A41.1, A41.2, A41.3, A41.4, A41.50, A41.51, A41.52, A41.53, A41.59, A41.81, A41.89,A41.9, A42.7, A54.86, B37.7, R65.20, R65.21, R78.81, T81.12XA)  DENOMINATOR Inclusions:  1.ICD-10-CM Other Diagnosis Codes for birth weight between 500 and 1499g (P05.02,P05.12, P05.2, P05.9, P07.0, P05.03, P05.13, P05.2, P05.9, P07.03, P05.04, P05.05, P05.14, P05.15, P05.2, P05.9, P07.14, P07.15)  2.ICD-10-CM Other Diagnosis Codes for birth weight ≥ 1500g (P05.06, P05.07, P05.16, P05.17, P05.2, P05.9, P07.16, P07.17,P05.08, P05.18, P05.2, P05.9, P07.18) OR Birth Weight ≥ 1500g who experienced one or more of the following:  2.1. Experienced death (dead and then revived/resuscitated)  2.2.ICD-10-PCS Principal Procedure Code or ICD-10-PCS Other Procedure Codes for major surgery  2.3. ICD-10-PCS Principal Procedure Code or ICD-10-PCS Other Procedure Codes for mechanical ventilation (5A1935Z, 5A1945Z, 5A1955Z) 2.4. Transferred in from another acute care hospital or health care setting within 2 days of birth.  Exclusions: 1.ICD-10-CM Principal Diagnosis Code for septicemias or bacteremias (A02.1, A22.7, A26.7, A32.7, A40.0, A40.1, A40.3, A40.8, A40.9, A41.01, A41.02, A41.1, A41.2, A41.3, A41.4, A41.50, A41.51, A41.52, A41.53, A41.59, A41.81, A41.89, A41.9, A42.7, A49.01, B37.7, B95.2, B95.61, B95.7, B95.8, B96.1, B96.20,B96.21, B96.22, B96.23, B96.29, B96.5, B96.89, R57.1, R57.8, R65.20, R65.21, R78.81, T81.10XA); 2. ICD-10-CM Other Diagnosis Codes for septicemias or bacteremias or ICD-10-CM Principal or Other Diagnosis Codes for newborn septicemia or bacteremia (P36.0, P36.10, P36.19, P36.2, P36.30, P36.39, P36.4, P36.5, P36.8, P36.9, R78.81); 3. ICD-10-CM Other Diagnosis Codes for birth weight <500g (P05.01, P05.11, P05.2, P05.9, P07.01); 4. Length of Stay < 2 days | Outcome | 2 | 2 | 2 | 19 |
|  | Late sepsis or meningitis in very low birth weight neonates | Infants with one or more of the following criteria:  Criterion 1: Bacterial Pathogen (A bacterial pathogen is recovered from a blood and/or cerebral spinal fluid culture obtained after day 3 of life) OR;  Criterion 2: Coagulase Negative Staphylococcus (the infant has all 3 of the following: 2.1. Coagulase negative staphylococcus is recovered from a blood culture obtained from either a central line, or peripheral blood sample and/or is recovered from cerebrospinal fluid obtained by lumbar puncture, ventricular tap or ventricular drain. 2.2. One or more signs of generalized infection (such as apnea, temperature instability, feeding intolerance, worsening respiratory distress or hemodynamic instability). 2.3. Treatment with 5 or more days of intravenous antibiotics after the above cultures were obtained. If the infant died, was discharged, or transferred prior to the completion of 5 days of intravenous antibiotics, this condition would still be met if the intention were to treat for 5 or more days | Eligible infants who are in the reporting hospital after day 3 of life | Exclusions:  1. Infants who meet neither of the above two criteria population (1.1 Any infant who is born at the reporting hospital and whose birth weight is between 401 and 1500 grams OR whose gestational age is between 22 weeks 0 days and 29 weeks 6 days (inclusive) should be included, regardless of where in the hospital the infant receives care; and 1.2 Any outborn infant who is admitted to any location in the reporting hospital within 28 days of birth, without first having gone home, and whose birth weight is between 401  and 1500 grams OR whose gestational age is between 22 weeks 0 days and 29 weeks 6 days (inclusive) should be included, regardless of where in the hospital the infant receives care)  2. Outborn infants admitted more than 28 days after birth.  3. Outborn infants who have been home prior to admission.  4. Infants discharged home on or before day 3 of life.  5. Infants who die on or before day 3 of life.  6. Infants who transfer to another hospital on or before day 3 of life and who are not readmitted to the reporting hospital | Outcome | 2 | 2 | 2 | 21 |
|  | Rate of assisted ventilation | Number of very low birth weight (VLBW) infants requiring the use of assisted ventilation for > 4 hours | All VLBW admissions | VLBW infants receiving continuous mechanical ventilation for > 4 hours for any reason (surgery or the need for controlled sedation to perform imaging studies is included). Mechanical ventilation includes nasal IMV/SIMV and high frequency/jet ventilation. Continuous positive airway pressure (CPAP) alone is not included in this measure  VLBW: <1500g | Outcome | 2 | 1 | 2 | 15 |
|  | Intracranial hemorrhage rate(IH) | Number of very low birth weight (VLBW) infants diagnosed with any degree of IH | All VLBW admissions with neural imaging in neonatal intensive care unit | The grade of IH is determined with neural imaging (CT scan, cranial ultrasound, or MRI) performed on or before day 28 of life.  Grade 1: Only subependymal germinal matrix hemorrhage; Grade 2: Intraventricular blood, no ventricular dilation; Grade 3: Intraventricular blood, ventricular dilation; Grade 4: Intraparenchymal hemorrhage  VLBW: <1500g | Outcome | 2 | 1 | 2 | 15 |
|  | Rate of Intracranial hemorrhage (IH) >grade 2 | Number of Very low birth weight (VLBW) infants diagnosed with IH >grade 2 | All VLBW admissions with neural imaging in neonatal intensive care unit | Grade 2: Intraventricular blood, no ventricular dilation; Grade 3: Intraventricular blood, ventricular dilation;  Grade 4: Intraparenchymal hemorrhage  VLBW: <1500g | Outcome | 2 | 1 | 2 | 15 |
|  | Rate of necrotizing enterocolitis (NEC) | Number of Very Low Birth Weight (VLBW) infants diagnosed with NEC | All VLBW admissions in neonatal intensive care unit | Infants with NEC diagnosed at surgery, at postmortem examination, or clinically and radiographically using the following criteria:  1. One or more of the following clinical signs present: Bilious gastric aspirate or emesis, abdominal distention, occult or gross blood in stool with no apparent rectal fissure AND 2. One or more of the following radiographic findings present: pneumatosis intestinalis, hepato-biliary gas, pneumoperitoneum.  This diagnosis includes infants classified as a "focal gastrointestinal perforation" if they meet the above criteria. Infants with focal GI perforation unassociated with clinical NEC are excluded.  VLBW: <1500g | Outcome | 2 | 1 | 2 | 15 |
|  | Rate of cystic Periventricular leukomalacia (PVL) | Number of very low birth weight (VLBW) infants diagnosed with PVL | All VLBW admissions with neural imaging in neonatal intensive care unit | Evidence of PVL indicated by multiple small periventricular cysts as shown by any neural imaging (cranial ultrasound, CT, or MRI)  Periventricular echogenicity without cysts should not be coded as PVL. A porencephalic cyst in the area of previously identified intraparenchymal hemorrhage is not coded as PVL. Periventricular abnormalities on CT or MI are not coded as PVL unless multiple small periventricular cysts are identified.  VLBW: <1500g | Outcome | 2 | 1 | 2 | 15 |
|  | Rate of retinopathy of prematurity (ROP) >stage 2 | Number of very low birth weight (VLBW) infants, <30 weeks gestational age at birth, diagnosed with ROP >stage 2 | VLBW survivors and <30 weeks gestational age at birth with an eye exam | Stage 2: Presence of intraretinal ridge; Stage 3: Presence of ridge with extraretinal fibrovascular proliferation; Stage 4: Partial retinal detachment; and Stage 5: Total retinal detachment  VLBW: <1500g | Outcome | 2 | 1 | 2 | 15 |
|  | Prevalence of selected congenital anomalies | Number of neural tube defects, oral clefts, and Down’s syndrome | Live births, fetal deaths and induced abortions. | Expressed as a proportion  Anencephaly and similar anomalies (ICD10-Q00) Spina bifida (ICD10-Q05) All congenital anomalies of the central nervous system (ICD10-Q00-Q07) Down's syndrome (ICD10-Q90) Cleft lip (ICD10-Q36) Cleft palate with cleft lip (ICD10-Q37) Cleft palate (without cleft lip) (ICD10-Q35) | Outcome | 2 | 3 | 3 | 13 |
|  | Rate of pneumothorax in neonatal intensive care unit (NICU) | Number of very low birth weight (VLBW) infants diagnosed with a pneumothorax | Inborn VLBW admissions in NICU | Pneumothorax: Extrapleural air diagnosed by chest radiograph or needle aspiration (thoracentesis) in the hospital. Also diagnosed for infants who had thoracic surgery and then later developed extrapleural air diagnosed by CXR or needle thoracentesis.  Excludes infants who had thoracic surgery and a chest tube was placed at the time of surgery OR if free air was only present on a CXR taken immediately after thoracic surgery and was not treated with a chest tube.  VLBW: <1500g | Outcome | 2 | 1 | 2 | 20 |
|  | Neonatal mortality rate by gestational age, birth weight, and plurality | Number of neonatal deaths (day 0 through 27) after live birth in a given year | Live births in the same year calculated by gestational age, birth weight and plurality | Expressed per 1000 live births.  Gestational age can be divided in: 22-23 weeks, 24-27 weeks, 28-31 weeks, 32-36 weeks and, $\geq$ 37 weeks.  Birth weight can be divided in: <500g, 500-999g, 1000-1499g, 1500-2499 g and, $\geq$2500 g.  Plurality is a multiple birth, resulting from a single pregnancy.  This rate is presented for all births at or after 22 completed weeks of gestation.  This rate is sub-divided by timing of death into early neonatal deaths (0-6 days of life) and late neonatal deaths (7-27 days) | Outcome | 3 | 3 | 3 | 13 |
|  | Rate of neonatal intensive care unit (NICU) mortality during hospitalization | Number of Very low birth weight (VLBW) deaths prior to 28 days of life | All VLBW NICU admissions | VLBW infant deaths prior to 28 days of life. This measure includes eligible outborn VLBW infants. This measure excludes deaths before 12 hours of life  VLBW: <1500g | Outcome | 2 | 1 | 2 | 15 |
|  | Early Neonatal Mortality rate | Number of neonatal deaths 0-7 days | Number of live births | Per 1000 live births | Outcome | 3 | 3 | 3 | 24 |
|  | Late neonatal mortality rate | Number of neonatal deaths 8-27 days | Number of live births | Per 1000 live births | Outcome | 3 | 3 | 3 | 24 |
|  | Neonatal mortality rate | Number of neonatal deaths | Number of live births | Per 1000 live births. Neonatal death is defined as a death during the first 28 days of life (0-27 days) | Outcome | 3 | 3 | 3 | 24 |
|  | Percent of infants born to HIV-infected mothers who are infected | Number of infants born to HIV-infected mothers who are HIV-infected | Total estimated number of HIV-infected pregnant women | Expressed as a percentage | Outcome | 3 | 3 | 1 | 24 |
|  | Percent of low birth-weight singleton live births, for multiparous women | Number of LBW singleton live births to women with two or more births <2500g | Number of singleton live births to women with two or more births | Expressed as a percentage.  Low birth weight (LBW) is defined as weight less than 2500 g obtained within 24 hours of birth, regardless of gestational age.  "Live birth" is the birth of a newborn, irrespective of the duration of gestation, that exhibits any sign of life, such as respiration, heartbeat, umbilical pulsation, or movement of voluntary muscles. | Outcome | 3 | 3 | 1 | 24 |
|  | Percent of low birth-weight singleton live births, for primiparous women | Number of LBW singleton live births to women with first birth <2500g | Number of singleton live births to women with first birth | Expressed as a percentage.  Low birth weight (LBW) is defined as weight less than 2500 g obtained within 24 hours of birth, regardless of gestational age.  "Live birth" is the birth of a newborn, irrespective of the duration of gestation, that exhibits any sign of life, such as respiration, heartbeat, umbilical pulsation, or movement of voluntary muscles. | Outcome | 3 | 3 | 1 | 24 |
|  | Percent of women with a live birth who reported seeking care from a skilled provider for a sick newborn | Number of women with live birth who report seeking care from a skilled provider for a sick newborn | Number of women with live birth during specified time period | Expressed as a percentage.  A sick newborn is defined as having a reported newborn illness in the first 28 days after birth.  Skilled providers can include private doctors, nurses, physician assistants, midwives and trained birth attendants, and pharmacists. | Outcome | 3 | 3 | 3 | 24 |
| **Others** (more than one phase) | Proportion of health professionals who use double gloves when attending a woman with a blood-borne disease | Health professionals who used double gloves | Health professionals who performed surgical procedures in woman with a blood- borne disease | Blood-borne can be bacterial or viral (hepatitis (B or C), HIV virus, and so on) | Process | 2 | 2 | 3 | 3 |
|  | Rate of prenatal & postpartum care | Prenatal care: deliveries that received a prenatal care visit as a patient of the organization in the first trimester or within 42 days of enrollment in the organization Postpartum Care: Deliveries that had a postpartum visit on or between 21 and 56 days after delivery | Deliveries of live births on measurement year | Expressed as a percentage  Women who had two separate deliveries (different dates of service) of the measurement year should be counted twice. Women who had multiple live births during one pregnancy should be counted once in the measure | Process | 1 | 2 | 2 | 8 |
|  | Blood transfusion during and/or after delivery | Number of women given blood transfusions during and/or after delivery | Number of women delivered | -- | Outcome | 2 | 3 | 2 | 18 |
|  | Incidence of severe maternal morbidity | Severe acute morbidity resulting during pregnancy, delivery or the puerperium period (<42 days) | Women delivering live or stillborn births | Express as a proportion  Severe maternal morbidity: Eclampsia (includes convulsion following specified or unspecified hypertensive disorders (that are not due to unknown epilepsy) during pregnancy, delivery or the puerperium. Corresponds to ICD-10 code O150)), Hysterectomy (surgical remove of the uterus (partial or total, body and/or cervix) for stopping the untreatable post partum haemorrhage) or embolisation (the process by which a blood vessel is obstructed by the lodgement of a material mass (or an embolus) to stop severe obstetric haemorrhage); Blood transfusion (all acts or processes of transferring blood into the vein, including transfusion of red blood cells, platelets (thrombocytes) and fresh frozen plasma) and; ICU >24 hours (admission during pregnancy, delivery or the puerperium to any facility or unit providing intensive or acute care or resuscitation-whether inside or outside of the maternity unit- for greater than 24 hours) | Outcome | 3 | 3 | 3 | 13 |
|  | Intra hospital women with life-threatening conditions (WLTC) ratio | Number of women with WLTC who developed these life-threatening conditions in the hospital | Number of livebirths in the hospital | Expressed per 1000 live births  WLTC: sum of maternal near miss (a woman who nearly died but survived a complication that occurred during pregnancy, childbirth or within 42 days of termination of pregnancy) and maternal deaths.  The death of a woman while pregnant or within 42 days of termination of pregnancy from any cause)  Live birth: complete expulsion or extraction from its mother of a product of conception, irrespective of the duration of the pregnancy, which, after such separation, breathes or shows any other evidence of life. Each product of such a birth is considered live born. | Outcome | 3 | 2 | 1 | 11 |
|  | Severe maternal outcome ratio | Number of women with life‐ threatening conditions | Live births | Expressed per 1000 live births | Outcome | 3 | 3 | 1 | 11 |
|  | Maternal near miss incidence ratio | Number of maternal near miss cases | Live births | Expressed per 1000 live births  Maternal near miss is a woman who nearly died but survived a complication that occurred during pregnancy, childbirth or within 42 days of termination of pregnancy.  Live birth: the complete expulsion or extraction from its mother of a product of conception, irrespective of the duration of the pregnancy, which, after such separation, breathes or shows any other evidence of life. Each product of such a birth is considered live born | Outcome | 3 | 2 | 1 | 11 |
|  | Met need for EmOC | Number of women treated for direct obstetric complications at EmOC facilities, over a defined period | Estimated number of women who would have major obstetric complications (or 15% of expected births, during the same defined period) | Expressed as a percentage.  The direct or major obstetric complications include:  -Hemorrhage: antepartum, intrapartum, or post­partum;  -Prolonged/obstructed labor;  -Postpartum sepsis;  -Complications of abortion;  -Severe pre-eclampsia/eclampsia;  -Ectopic pregnancy; and  -Ruptured uterus.  Number of women with a major obstetric complication includes both women admitted with the complication and women who develop the complication in the facility.  EmOC facilities include both basic and comprehen­sive levels of essential obstetric care. | Outcome | 3 | 2 | 3 | 24 |
|  | Intrapartum and very early neonatal death rate | Number of births weighing ≥ 2.5 kg resulting in intrapartum deaths and very early neonatal deaths within first 24 hours | Number of women giving birth to infants weighing ≥2.5 kg during specified time frame | Expressed as a percentage.  Intrapartum or fresh stillbirths include infants born dead after 28 weeks of gestation without skin deterioration or maceration. The death is assumed to have occurred less than 12 hours before delivery and excludes infants with severe, lethal congenital abnormalities.  Early neonatal deaths include neonates born at term who could not be resuscitated, for whom resuscitation was not available, or who had a specific birth trauma, where death occurred within 24 hours of delivery. | Outcome | 3 | 3 | 3 | 24 |
|  | Feto-infant mortality rate | Number of feto-infant deaths ≥ 20 weeks’ gestation according to four levels of birth weight and age-at-death | Total births | Per 1000 births.  Level of birth weight (500-999 g, 1000-1499 g, 1500-2499 g, ≥ 2500 g).  Level of age-at-death: fetal (20+ weeks), early neonatal (<7 days), late neonatal (7-27 days), post-neonatal (28+ days) | Outcome | 3 | 3 | 2 | 16 |
|  | Fetal mortality rate by gestational age, birth weight, and plurality | Number of fetal deaths at or after 22 completed weeks of gestation in a given year | Live and stillbirths in the same year calculated by gestational age, birth weight and plurality | Expressed per 1000 live and stillbirths. Calculated by gestational age: 22-27 weeks, 28-31 weeks, 32-36 weeks, and $\geq$ 37 weeks.  Birth weight is divided in: <1000g, 1000-1499g, 1500-2499 g, and $\geq$2500 g.  Plurality is defined as a multiple birth, resulting from a single pregnancy | Outcome | 3 | 3 | 3 | 13 |
|  | Fetal and neonatal deaths due to congenital anomalies | Number of deaths in the fetal and neonatal period attributed to congenital anomalies | Number of total births | Expressed as a percentage  Fetal deaths: at or after 22 completed weeks of gestation  Neonatal deaths (day 0 through 27) after live birth.  Congenital anomalies: Anencephaly and similar anomalies (ICD10-Q00) Spina bifida (ICD10-Q05) All congenital anomalies of the central nervous system (ICD10-Q00-Q07) Down's syndrome (ICD10-Q90) Cleft lip (ICD10-Q36) Cleft palate with cleft lip (ICD10-Q37) Cleft palate (without cleft lip) (ICD10-Q35) | Outcome | 3 | 3 | 3 | 13 |
|  | Birth weight-specific feto-infant mortality rate for very low birth weight (VLBW) infants | Number of feto- infant deaths for infants with birth weight 500-1499 grams | Number of births (livebirths and stillbirths) for infants with birth weight 500-1499 grams | VLBW: 500-1499 grams Feto-infant deaths: gestational age ≥ 20 week and ≥28 days of birth | Outcome | 3 | 3 | 2 | 16 |
|  | Proportionate mortality rate | Total number of feto-infant deaths in each category | Total number of feto-infant deaths | Per 1000 feto-infant deaths  Category 1: includes Fetal Death (20+ weeks), Early Neonatal Death (<7 days), Late  Neonatal Death (7-27days) and Post Neonatal Death (28+ days) with very very low birth weight (VVLBW) (500-999gms), very low birth weight (VLBW)(999-1499 gms); Category 2: includes Fetal Death (20+ wks) with Low Birth Weight (LBW)(1499-2499 gms) and Normal Birth Weight (NBW) (2500+ gms).  Category 3: includes Early  Neontal Death (<7 days) with IBW (1499-2499 gms) or NBW (2500+ gms; AND Late Neonatal Death (7-27days) with IBW (1499-2499 gms). Category 4: includes Late  Neonatal Death (7-27days) with NBW (2500+ gms) AND Post Neonatal Death  (28+ days) with IBW (1499-2499 gms) and NBW (2500+ gms). | Outcome | 3 | 3 | 2 | 16 |
|  | Excess feto-infant mortality rate (FIMR) for each intervention category | 1. Number of feto-infant deaths ≥ 20 weeks’ gestation according to four levels of birth weight and age-at-death for category for the reference population  2. Number of feto-infant deaths ≥ 20 weeks’ gestation according to four levels of birth weight and age-at-death for category for the population of interest | 1.Total number of feto-infant deaths for the reference population  2.Total number of feto-infant deaths for the population of interest | Per 1000 feto-infant deaths in the population.  Subtracted the proportionate mortality rate for the reference population from the proportionate mortality rate for the population of interest (i.e, that which achieved the lowest FIMR) | Outcome | 3 | 3 | 2 | 16 |
|  | Stillbirth rate | Number of fetuses and infants born per year with no sign of life and born after 28 weeks gestation, or weighing ≤1000 g | Total births | Per 1000 live births | Outcome | 2 | 3 | 3 | 25 |
|  | Maternal mortality ratio | Number of maternal deaths | Number of live births | Per 100 000 live births Maternal death is the death of a woman while pregnant or within 42 days of termination of pregnancy, irrespective of the duration and site of the pregnancy, from any cause related to or aggravated by the pregnancy or its management but not from accidental or incidental causes. | Outcome | 3 | 3 | 1 | 12 |
|  | Maternal near miss: mortality ratio | Maternal near miss cases | Maternal deaths | Expressed as proportion between maternal near miss cases (MNM) and maternal deaths (MD).  [MNM : 1 MD] | Outcome | 3 | 2 | 1 | 11 |
|  | Case fatality rate | Number of maternal deaths | Number of women with life-threatening conditions | Expressed as a percentage | Outcome | 3 | 2 | 1 | 11 |
|  | Case fatality rate- all complications | Number of deaths from specified obstetric complications in a facility | Number of women with specified obstetric complications attended in the facility | Expressed as a percentage.  Where deaths from the following complications are included:  -Hemorrhage: antepartum, intrapartum or post­partum;  -Prolonged/obstructed labor;  -Postpartum sepsis;  -Complications of abortion;  -Pre-eclampsia/eclampsia;  -Ectopic pregnancy; and  -Ruptured uterus. | Outcome | 3 | 3 | 1 | 24 |
|  | Institutional maternal mortality ratio (per 100 000 deliveries) | Number of maternal deaths in institutions | Number of deliveries in institutions | Per 100 000 deliveries in health facilities/institutions | Outcome | 3 | 2 | 3 | 26 |
|  | Intra hospital mortality index | Number of maternal deaths who were not ill on arrival | Number of women with life-threatening conditions who were not ill on arrival | Expressed as percentage | Outcome | 3 | 2 | 1 | 11 |
| *Level of care*: 1. Primary health care; 2: Hospital care (second or third level of care); 3:both  *Organizational level of application:* 1. Service or specific unit within a health facility; 2. Health facility; 3. Supra-institutional level (region, State or national)  *Income level of the country*: 1. Medium and low income; 2. High income; 3. High, medium or low income (all) | | | | | | | | | |

**Additional file 3b.** Descripción de los indicadores en español

| **Etapa del *continuum*** | **Nombre del indicador** | **Numerador** | **Denominador** | **Aclaraciones y/o definición operativa** | **Tipo de indicador** | **Nivel de atención** | **Nivel de aplicación** | **Nivel de ingreso** | **Referencia**  **#** |
| --- | --- | --- | --- | --- | --- | --- | --- | --- | --- |
| **Embarazo** | Frecuencia de control prenatal apropiado | Mujeres con un número apropiado de consultas de control prenatal esperadas | Partos con nacidos vivos en un año | El numerador considera el número de mujeres clasificadas en cada una de las 5 categorías siguientes: proporción de visitas esperadas <21%; 21%–40%; 41%–60%; 61%–80%; o mayor o igual 81%.  Criterios de exclusión: nacidos muertos | Proceso | 1 | 3 | 2 | 1 |
|  | Porcentaje de mujeres que recibieron atención prenatal a las 13 semanas de gestación | Número de mujeres embarazadas con cita a las 13 semanas de gestación | Número de mujeres embarazadas | -- | Proceso | 1 | 3 | 2 | 2 |
|  | Porcentaje de mujeres con eclampsia tratadas con sulfato de magnesio | Mujeres tratadas con sulfato de magnesio | Mujeres con eclampsia | Código CIE-9: 642.6.  Criterios de exclusión: mujeres con contraindicación a sulfato de magnesio | Proceso | 2 | 2 | 3 | 3 |
|  | Porcentaje de mujeres con preeclampsia grave que fueron tratadas con sulfato de magnesio | Mujeres tratadas con sulfato de magnesio | Mujeres con preeclampsia grave | Código CIE-9: 642.5.  Criterios de exclusión: mujeres con contraindicación a sulfato de magnesio | Proceso | 2 | 2 | 3 | 3 |
|  | Porcentaje de mujeres con un embarazo único y amenaza de parto prematuro que recibieron corticoides | Mujeres que recibieron corticoides | Embarazos únicos con 26-34 semanas de gestación | Códigos CIE-9: 644.03, 644.10, 644.13, 644.20, 644.21.  Criterios de exclusión: mujeres con contraindicación a los corticoides | Proceso | 2 | 2 | 3 | 3 |
|  | Porcentaje de mujeres con amenaza de parto prematuro a las que se le administró sulfato de magnesio | Mujeres a las que se le administró sulfato de magnesio | Mujeres que recibieron tratamiento farmacológico por amenaza de trabajo de parto prematuro | Códigos CIE-9: 644.03, 644.10, 644.13, 644.20, 644.21 | Proceso | 2 | 2 | 3 | 3 |
|  | Porcentaje de mujeres que recibieron bloqueadores de los canales de calcio (BCC) para la inhibición del trabajo de parto prematuro | Mujeres que recibieron BCC | Mujeres embarazadas de 22 a 23 semanas de gestación | Códigos CIE-9: 644.03, 644.10, 644.13, 644.20, 644.21.  Criterios de exclusión: mujeres con contraindicación a BCC | Proceso | 2 | 2 | 3 | 3 |
|  | Porcentaje de mujeres con ceguera nocturna en el último embarazo | Número de mujeres con ceguera nocturna en el último embarazo | Número total de mujeres con embarazo, con recién nacido vivo en los últimos 3 años | Expresado como porcentaje.  La ceguera nocturna es la incapacidad para ver normalmente después del atardecer o por la noche durante el embarazo, especialmente el último trimestre y temprano postparto.  Se debe excluir a mujeres cuya ceguera probablemente debido a la debilidad visual por otras causas. | Resultado | 1 | 3 | 3 | 24 |
| **Parto** | Porcentaje de mujeres que recibieron apropiada profilaxis antibiótica una hora antes de la incisión quirúrgica por cesárea | Porcentaje de mujeres que recibieron antibióticos una hora antes de la cesárea | Todas las mujeres embarazadas sometidas a cesárea sin evidencia de infección previa y sin haber recibido antibióticos profilácticos por otras razones | Criterios de exclusión: Mujeres con evidencia de infección previa o que ya recibieron antibióticos profilácticos por otras razones; o mujeres con alergias significativas a penicilina y/o cefalosporinas y alergias a gentamicina y/o clindamicina | Proceso | 2 | 2 | 2 | 5 |
|  | Porcentaje de mujeres que recibieron profilaxis antibiótica intraparto por riesgo de transmisión por estafilococos del grupo B (EGB) | Mujeres que recibieron profilaxis antibiótica intraparto por EGB | Todas las mujeres que dieron a luz a niños vivos, excepto las que no tienen riesgo de transmisión vertical de EGB | Criterios de exclusión del denominador: 1) pacientes con resultado negativo de EGB a las 35-37 semanas de gestación al momento del parto; 2) pacientes que realizan una cesárea planificada; 3) pacientes están tomando antibióticos para una infección o profilaxis; y 4) nacidos muertos(CIE-9- V.27.1, V27.3, V27.4, V27.6 or V27.7) | Proceso | 2 | 2 | 2 | 6 |
|  | Porcentaje de mujeres sometidas a cesárea con profilaxis para trombosis venosa profunda | Número de mujeres sometidas a un parto por cesárea que recibieron un dispositivo de compresión neumática o profilaxis médica antes de la cirugía | Todas las mujeres sometidas a un parto por cesárea | HCPCS códigos: J1644, J1650, J1645, J1655 | Proceso | 2 | 2 | 2 | 4 |
|  | Porcentaje de mujeres con cesárea que recibieron terapia antibiótica | Mujeres que recibieron antibióticos | Mujeres a las que se le realizó cesárea | Código CIE-9: 74.XX. El valor "X" puede ser sustituido por cualquier número entre 0 y 9.  La terapia antibiótica incluye todo tipo de antibióticos (no se recomienda amoxicilina/ácido clavulánico ) | Proceso | 2 | 2 | 3 | 3 |
|  | Porcentaje de mujeres con ruptura prematura de membranas que recibieron tratamiento con antibióticos | Mujeres que recibieron antibióticos | Embarazos de 22-34 semanas de gestación con ruptura prematura de membranas | Códigos CIE-9: 658.10, 658.11  La terapia antibiótica incluye todo tipo de antibióticos (no se recomienda amoxicilina/ácido clavulánico) | Proceso | 2 | 2 | 3 | 3 |
|  | Porcentaje de mujeres a las que se les administró uterotónicos en la tercera etapa del trabajo de parto | Mujeres a las que se les administró uterotónicos en la tercera etapa del trabajo de parto | Mujeres en trabajo de parto | Código CIE-9: 641.X1, 642.X1, 676.X1.  El valor "X" puede ser sustituido por cualquier número entre 0 y 9.  Se puede administrar cualquier uterotónico p.ej. oxitocina (intravenosa (IV) o intramuscular (IM)); sintometrina (IM); ergometrina (IV o IM); misoprostol (IM).  Criterios de exclusión: mujeres con contraindicación a uterotónicos o rechazo a recibir uterotónicos. | Proceso | 2 | 2 | 3 | 3 |
|  | Porcentaje de mujeres con parto a término con examen de detección de estafilococos del grupo B (EGB)entre la semana 35-37 de gestación | Número de mujeres con trabajo de parto a término que tuvieron tamizaje de EGB entre la semana 35-37 de gestación | Número total de mujeres en trabajo de parto a término | Parto a término: ≥37 semanas de gestación | Proceso | 2 | 2 | 2 | 9 |
|  | Porcentaje de mujeres con embarazos a término y presentación pelviana a las que se les realizó u ofreció versión cefálica externa | Mujeres en las que se realizó u ofreció la versión cefálica externa | Partos con presentación pelviana | Código CIE-9: 73.91 | Proceso | 2 | 2 | 3 | 3 |
|  | Porcentaje de mujeres con inducción del trabajo de parto con menos de 41 semanas de gestación y que tenía indicación de inducción post-término | Número de mujeres inducidas que tenían menos de 41 semanas de gestación al momento del parto | Número total de mujeres con inducción del trabajo de parto con indicación de inducción post-término | Post-término: ≥41 semanas de gestación | Proceso | 2 | 2 | 2 | 9 |
|  | Porcentaje de mujeres que dieron a luz de forma inducida después de las 41 semanas de gestación | Mujeres con inducción del trabajo de parto | Mujeres con embarazo mayor o igual a 41 semanas de gestación | Códigos CIE-9: 641.X1, 642.X1, 676.X. El valor "X" puede ser sustituido por cualquier número entre 0 y 9.  Criterio de exclusión: trabajo de parto espontáneo y parto no urgente por cesárea. | Proceso | 2 | 2 | 3 | 3 |
|  | Porcentaje de mujeres con desgarros perineales de segundo grado o episiotomía que fueron reparados con sutura continua | Mujeres a las que se les realizó sutura continua | Mujeres con desgarros perineales de segundo grado o episiotomía | Códigos CIE-9: 664.10, 664.11, 644.14, 73.6. | Proceso | 2 | 2 | 3 | 3 |
|  | Porcentaje de mujeres embarazadas que tenían programada una cesárea a las que se les realizó en o después de la semana 39 de gestación | Número de mujeres a las que se les realizó una cesárea en o después de la semana 39 de gestación | Número de mujeres a las que se les realizó una cesárea programada sin indicación materna o fetal para un parto temprano | Indicaciones maternas o fetales: condiciones importantes, enfermedad hipertensiva, diabetes o diabetes gestacional, hemorragia antes del parto, restricción del crecimiento intrauterino, anomalías congénitas, hidropesía, incompatibilidad del grupo sanguíneo, sufrimiento fetal agudo o embarazo múltiple | Proceso | 2 | 2 | 2 | 2 |
|  | Tasa de cesárea repetida antes de las 39 semanas de gestación en mujeres con bajo riesgo | Número de mujeres con bajo riesgo a las que se les realizó cesárea antes de las 39 semanas de gestación | Número total de mujeres con bajo riesgo que tuvieron una cesárea repetida a término (≥37 semanas) | Indicador expresado en porcentaje  Cesárea repetida: cesárea realizada en mujeres con antecedentes de una o más cesáreas anteriores.  Inclusiones del denominador: mujeres con un único nacimiento vivo, entre 37 y 42 semanas de edad gestacional, sin problemas médicos maternos, sin complicaciones obstétricas y ninguna de las siguientes indicaciones para la cesárea: prolapso del cordón, anomalía fetal, restricción del crecimiento intrauterino/pequeño para la edad gestacional, grande para la edad gestacional, estado fetal inestable, placenta previa, desprendimiento placentario, pre-eclampsia y rotura prematura de membranas. | Proceso | 3 | 2 | 2 | 9 |
|  | Porcentaje de episiotomías injustificadas | Episiotomía realizada sin justificación documentada | Mujeres a las que se les realizó episiotomía | Código CIE-9: 73.6 | Proceso | 2 | 2 | 3 | 3 |
|  | Porcentaje de mujeres con rasurado pubiano o perineal en el ingreso a la sala de partos | Mujeres para quienes se realizó rasurado pubiano o perineal | Mujeres en trabajo de parto | Código CIE-9: 641.X1, 642.X1, 676.X1. El valor "X" puede ser sustituido por cualquier número entre 0 y 9 | Proceso | 2 | 2 | 3 | 3 |
|  | Porcentaje de mujeres a las que se les suturó el peritoneo en la cesárea | Mujeres a las que se les suturó el peritoneo | Mujeres a las que se le realizó cesárea | Código CIE-9: 74.XX. El valor "X" puede ser sustituido por cualquier número entre 0 y 9 | Proceso | 2 | 2 | 3 | 3 |
|  | Porcentaje de mujeres a las que se les realizó un enema durante el trabajo de parto | Número de mujeres que se les realizó un enema | Mujeres en trabajo de parto | Código CIE-9: 641.X1, 642.X1, 676.X1. El valor "X" puede ser sustituido por cualquier número entre 0 y 9 | Proceso | 2 | 2 | 3 | 3 |
|  | Tasa de incidencia de ruptura uterina | Número de mujeres con ruptura uterina | Número total de mujeres que dieron a luz | -- | Resultado | 2 | 1 | 2 | 18 |
|  | Proporción de mujeres con trabajo de parto prolongado | Número de mujeres que no han dado a luz o que no fueron trasladadas en las primeras 12 hrs con trabajo de parto activo | Número total de mujeres en trabajo de parto en el establecimiento de salud | Trabajo de parto activo: debe estar documentada una dilatación cervical ≥ 4 cm y con documentación clara de la hora del parto | Resultado | 3 | 2 | 3 | 14 |
|  | Porcentaje de nacimientos sin intervención obstétrica | Número de partos sin intervenciones obstétricas seleccionadas | Partos que resultan en un nacido vivo o nacido muerto | Nacimientos sin intervención obstétrica: partos en mujeres cuyo parto comienza espontáneamente, progresa espontáneamente sin medicación y que da a luz espontáneamente.  Intervenciones obstétricas seleccionadas: mujeres que experimentaron uno o más de los siguientes: inducción del parto (con prostaglandinas, oxitócica o rotura artificial de membranas), anestesia epidural o espinal o general, fórceps o ventosa, cesárea, episiotomía. | Resultado | 2 | 3 | 3 | 22 |
|  | Tasa de parto vaginal instrumentado | Número de neonatos que dieron a luz por extracción instrumental | Número total de partos vaginales (todos los neonatos nacidos vivos incluyendo aquellos con defectos en el nacimiento) | Extracción instrumental: fórceps obstétrico o ventosa | Resultado | 2 | 1 | 2 | 18 |
|  | Incidencia de cesárea antes del parto | Número de cesáreas antes del parto | Número total de mujeres que dieron a luz | -- | Resultado | 2 | 1 | 2 | 18 |
|  | Incidencia de cesárea durante el parto | Número de cesáreas durante el parto | Número total de mujeres que dieron a luz | -- | Resultado | 2 | 1 | 2 | 18 |
|  | Tasa de episiotomía | Número de mujeres que han tenido una episiotomía | Mujeres que tuvieron un parto vaginal | Indicador expresado como proporción  Los datos se presentan para todos los partos vaginales y luego para los no instrumentados e instrumentados por separado | Resultado | 2 | 3 | 3 | 13 |
|  | Incidencia de desgarro en el perineo | Número de mujeres que presentaron un desgarro de primer, segundo, tercer o cuarto grado del perineo | Mujeres que tuvieron un parto vaginal | Indicador expresado como proporción.  para todos los partos vaginales, y luego para no instrumental e instrumental por separado. Categorías: 1er grado (CIE-10 O70.0) involucra el frenillo de los labios menores, la piel perineal y la membrana vaginal; 2o grado perineal (CIE-10 O70.1) incluye la piel y la membrana mucosa, así como la fascia y los músculos del cuerpo ; 3er grado (CIE-10 O70.2) se extiende a través de la piel, la membrana mucosa y el cuerpo perineal e involucra el músculo del esfínter anal; y 4o grado (ICD O70.3) se extiende a través de la mucosa rectal para exponer la luz del recto. | Resultado | 2 | 3 | 3 | 13 |
|  | Incidencia de transferencia y/o admisión materna en la unidad de cuidados intensivos (UCI) | Número de mujeres transferidas y/o admitidas en UCI | Número total de mujeres que dieron a luz | -- | Resultado | 2 | 1 | 2 | 18 |
|  | Tasa de mortalidad intraparto | Número de niños nacidos muertos que pesan más de 1000 gr y con frecuencia cardiaca fetal documentada en admisión | Número total de nacimientos de niños con un peso mayor a 1000 gr en el establecimiento | -- | Resultado | 3 | 2 | 3 | 14 |
|  | Proporción de mujeres atendidas por complicaciones obstétricas que están relacionadas con un aborto | Número de mujeres con complicaciones por aborto tratadas en un establecimiento | Número de mujeres con complicaciones obstétricas atendidas en las instalaciones o establecimientos de salud | Complicaciones relacionadas con aborto: aborto incompleto, aborto fallido, hemorragia, infección, perforación uterina | Resultado | 3 | 2 | 1 | 17 |
|  | Proporción de mujeres tratadas por complicaciones del aborto que son graves | Número de mujeres con complicaciones graves del aborto tratadas | Número de mujeres con alguna complicación del aborto tratadas en un establecimiento de salud | Complicaciones del aborto: hemorragia, infección, perforación uterina | Resultado | 3 | 2 | 1 | 17 |
| **Puerperio** | Porcentaje de mujeres con resultado de Rh-negativo que recibieron Anti-D dentro de las 72 horas tras el parto de un bebé con Rh-positivo o Rh-indeterminado | Mujeres que recibieron Anti-D | Mujeres Rh-negativas con recién nacido con Rh-positivo o Rh-indeterminado | Criterios de exclusiones: mujeres con sensibilización de Rh previa | Proceso | 2 | 2 | 3 | 3 |
|  | Proporción de mujeres con infección sistémica grave o sepsis en el período postnatal, incluyendo reingresos | Número de mujeres atendidas en el establecimiento con infección sistémica grave o sepsis en el período postnatal, incluyendo reingresos después del parto en el establecimiento | Número total de mujeres que dan a luz en el establecimiento de salud | Período postnatal: los primeros 42 días después del parto | Resultado | 2 | 2 | 3 | 14 |
| **Recién nacido** | Porcentaje de nacidos vivos en los que se realizó examen auditivo antes del alta hospitalaria | Nacidos vivos a los que se les realizó un tamizaje para la pérdida auditiva antes del alta o que no fueron examinados por razones médicas o exclusiones médicas en el establecimiento de salud | Todos los nacidos vivos en el establecimiento de salud donde el recién nacido fue dado de alta con ≤120 días durante su hospitalización | Criterios de exclusiones del denominador: Pacientes fallecidos antes del alta y que no recibieron tamizaje auditivo del oído izquierdo o derecho | Proceso | 2 | 2 | 2 | 10 |
|  | Cobertura intrahospitalaria de vacuna contra hepatitis B en recién nacidos vivos antes de ser dados de alta del establecimiento de salud | Número de recién nacidos vivos que recibieron la vacuna contra la hepatitis B antes de ser dado de alta del establecimiento de salud o en el primer mes de vida (en caso de una estancia hospitalaria más prolongada) | Número de recién nacidos vivos en el establecimiento de salud | Criterios de inclusión del numerador: CIE-10 Z37.0 ,Z37.2, Z37.3, Z37.50, Z37.51, Z37.52, Z37.53, Z37.54, Z37.59, Z37.60, Z37.61, Z37.62, Z37.63, Z37.64, Z37.69, Z38.00, Z38.001, Z38.1, Z38.2, Z38.30 , Z38.31 , Z38.4, Z38.5, Z38.61 ,Z38.62 ,Z38.63, Z38.64, Z38.65, Z38.66, Z38.68, Z38.69, Z38.7, Z38.8.  Criterios de inclusión del denominador: recién nacidos en el cual el padre o tutor negó la administración de la vacuna (CIE-10 Z28.82) | Proceso | 2 | 2 | 2 | 7 |
|  | Tasa de bebés nacidos a término a los que se les dio fórmula suplementada y cuya madre tenía la intención de amamantar | Número de bebés nacidos a término que recibieron fórmula suplementada | Número de recién nacidos a término cuyas madres tenían la intención de amamantar | Indicador expresado en porcentaje | Proceso | 2 | 2 | 2 | 9 |
|  | Proporción de bebés que fueron alimentados exclusiva o parcialmente con leche materna durante 6-8 semanas después del parto | Niños que fueron alimentados exclusiva o parcialmente de leche materna durante 6 a 8 semanas tras del parto | Número de niños con chequeo postnatal durante las semanas 6 a 8 después del parto | -- | Proceso | 1 | 3 | 2 | 2 |
|  | Tasa de muy bajo peso al nacer | Número de niños con peso al nacer de 500-1499 gramos | Número de nacimientos (nacidos vivos y nacidos muertos) | -- | Resultado | 3 | 3 | 2 | 16 |
|  | Porcentaje de nacidos vivos a término que NO TIENEN complicaciones durante el parto o el cuidado del recién nacido en el hospital | Nacidos vivos a término en parto único con ausencia de alguna complicación durante el parto o el cuidado del recién nacido en el hospital | Todos los nacidos vivos a término (>= 37 semanas) en parto único | Criterios de inclusión del denominador: No se excluyen las afecciones maternas y obstétricas (p.e. hipertensión, cesárea previa, etc.) a menos que exista evidencia de efecto fetal antes del parto; Exclusión: condiciones fetales presentes antes del parto | Resultado | 1 | 1 | 2 | 23 |
|  | Porcentaje de niños de ≥ 37 semanas de gestación con Apgar <7 a los 5 min | Número de nacimientos ≥ 37 semanas con Apgar < 7 a los 5 min | Número total de nacimientos con ≥ 37 semanas de gestación | -- | Resultado | 2 | 1 | 2 | 18 |
|  | Porcentaje de recién nacidos de alto riesgo con septicemias estafilocócicas y gram negativas o bacteriemias | Recién nacidos con septicemia o bacteriemia o sepsis con infección sanguínea confirmada | Nacidos vivos recién nacidos ≤1500 gramos | Criterios de inclusión del numerador:  1. Diagnóstico secundario de septicemia o bacteriemia de recién nacidos (CIE-10 P36.0, P36.10, P36.19, P36.2, P36.30, P36.39, P36.4, P36.5, P36.8, P36.9, R78.81)  2. Diagnóstico secundario para la sepsis (CIE-10 A02.1, A22.7, A26.7, A32.7, A40.0, A40.1, A40.3, A40.8, A40.9, A41.01, A41.02, A41.1, A41.2, A41.3, A41.4, A41.50, A41.51, A41.52, A41.53, A41.59, A41.81, A41.89,A41.9, A42.7, A54.86, B37.7, R65.20, R65.21, R78.81, T81.12XA)  Criterios de inclusión del denominador: Diagnóstico de peso al nacer entre 500 y 1499g (CIE-10 P05.02,P05.12, P05.2, P05.9, P07.0, P05.03, P05.13, P05.2, P05.9, P07.03, P05.04, P05.05, P05.14, P05.15, P05.2, P05.9, P07.14, P07.15) o peso al nacer ≥ 1500g (P05.06, P05.07, P05.16, P05.17, P05.2, P05.9, P07.16, P07.17,P05.08, P05.18, P05.2, P05.9, P07.18) y además con uno o más de los siguientes: 2.1 experiencias cercanas a la muerte, 2.2Procedimiento de cirugía mayor, 2.3 Procedimiento de ventilación mecánica(CIE-10 5A1935Z, 5A1945Z, 5A1955Z), y trasladado desde otro hospital de cuidados agudos o establecimiento de atención dentro de los 2 días posteriores al nacimiento.  Criterios de exclusión: Diagnóstico de septicemias o bacteriemias al ingreso (A02.1, A22.7, A26.7, A32.7, A40.0, A40.1, A40.3, A40.8, A40.9, A41.01, A41.02, A41.1, A41.2, A41.3, A41.4, A41.50, A41.51, A41.52, A41.53, A41.59, A41.81, A41.89, A41.9, A42.7, A49.01, B37.7, B95.2, B95.61, B95.7, B95.8, B96.1, B96.20,B96.21, B96.22, B96.23, B96.29, B96.5, B96.89, R57.1, R57.8, R65.20, R65.21, R78.81, T81.10XA), Diagnóstico de septicemias o bacteriemias al ingreso o con diagnóstico de septicemia o bacteriemia neonatal con una infección del torrente sanguíneo presente en la admisión (P36.0, P36.10, P36.19, P36.2, P36.30, P36.39, P36.4, P36.5, P36.8, P36.9, R78.81) , peso al nacer <500g (P05.01, P05.11, P05.2, P05.9, P07.01), y duración de la estancia <2 días. | Resultado | 2 | 2 | 2 | 19 |
|  | Incidencia de sepsis tardía o meningitis en neonatos de muy bajo peso al nacer | Niños con uno o más de los siguientes criterios:  Criterio 1: Patógeno Bacteriano (se recupera de un cultivo de sangre y/o líquido cefalorraquídeo obtenido después del día 3 de vida)  Criterio 2: Estafilococo coagulasa negativos (el bebé tiene todos los 3 siguientes: 2.1. obtención del estafilococo coagulasa negativo a través de los cultivos (hemocultivo obtenido de una línea central o de una muestra de sangre periférica y/o se recupera del líquido cefalorraquídeo obtenido mediante punción lumbar, punción ventricular o drenaje ventricular), 2.2. Uno o más signos de infección generalizada (como apnea, inestabilidad de temperatura, intolerancia a la alimentación, empeoramiento de la dificultad respiratoria o inestabilidad hemodinámica y, 2.3. Tratamiento con 5 o más días de antibióticos intravenosos después de la obtención de los cultivos anteriores. Si el infante murió, fue dado de alta, o transferido antes de haber completado 5 días de antibióticos intravenosos, este criterio todavía se cumplirá si había intención de tratar durante 5 o más días | Bebés elegibles que están en el hospital de reporte después de tres días de vida | Criterios de exclusión: 1. Cualquier niño que no cumpla con ninguna de las siguientes condiciones está excluido (1.1 Peso al nacer entre 401 y 1500 gramos o con Edad gestacional entre 22 y 29 semanas, 1.2 neonatos nacidos fuera del hospital que ingresan en el hospital más de 28 días después del nacimiento están excluidos) 2. Neonatos nacidos fuera del hospital que han estado en casa antes de ser admitidos en el hospital están excluidos, 3. Bebés dados de alta en el día 3 o antes del día 3 de vida, 4. Bebés que mueren en o antes del día 3 de vida, 5. Bebés que se trasladan a otro hospital en o antes del día 3 de vida y que no son reingresados en el hospital, y 6. Bebés que son transferidos más de una vez antes del tercer día de vida | Resultado | 2 | 2 | 2 | 21 |
|  | Tasa de uso de ventilación asistida | Número de niños de muy bajo peso al nacer (MBPN) que necesitaron el uso de ventilación asistida durante más de 4 horas | Todos los ingresos de niños con MBPN | Los niños de MBPN que reciben ventilación mecánica continua durante más de 4 horas por cualquier razón (cirugía o la necesidad de sedación controlada para realizar estudios de imagen está incluido). La ventilación mecánica incluye ventilación nasal intermitente obligatoria o ventilación obligatoria intermitente sincronizada y ventilación de alta frecuencia / chorro. La presión positiva continua de las vías respiratorias (CPAP) por sí sola no se incluye en este indicador.  MBPN: <1500 gramos | Resultado | 2 | 1 | 2 | 15 |
|  | Tasa de hemorragia intracraneal | Número de niños con muy bajo peso al nacer (MBPN) diagnosticados con cualquier grado de hemorragia intracraneal | Todos los ingresos de niños con MBPN con neuroimagen realizada en la unidad de cuidado intensivo neonatal | El grado de hemorragia intracraneal se determina a partir de imágenes neurales (tomografía computarizada, ecografía craneal o resonancia magnética) realizadas en o antes del día 28 de vida. Grado 1: Sólo hemorragia subependimaria de la matriz germinal; Grado 2: Sangre interventricular, sin dilatación ventricular; Grado 3: Sangre intraventricular, dilatación ventricular; Grado 4: Hemorragia intraparenquimatosa.  MBPN: <1500g | Resultado | 2 | 1 | 2 | 15 |
|  | Tasa de hemorragia intracraneal grado >2 | Número de niños con muy bajo peso al nacer (MBPN) diagnosticados con hemorragia intracraneal mayor a grado 2 | Todos los ingresos de niños con MBPN con neuroimagen realizada en la unidad de cuidado intensivo neonatal | Grado 2: Sangre interventricular, sin dilatación ventricular; Grado 3: Sangre interventricular, dilatación ventricular; Grado 4: Hemorragia intraparenquimatosa.  MBPN: <1500g | Resultado | 2 | 1 | 2 | 15 |
|  | Tasa de enterocolitis necrotizante (EN) | Número de niños con muy bajo peso al nacer (MBPN) diagnosticados de EN | Todos los ingresos de niños con MBPN en la unidad de cuidado intensivo neonatal | Criterios de inclusión: niños con EN diagnosticados en la cirugía, en el examen post mortem, o clínica y radiográficamente usando los siguientes criterios: 1. Uno o más de los siguientes signos clínicos presentes: aspiración gástrica o emesis biliares, distensión abdominal, sangre oculta en las heces sin fisura rectal aparente. Y 2. uno o más de los siguientes hallazgos radiográficos presentes: pneumatosis intestinalis, gas hepatobiliar, pneumoperitoneo. Este diagnóstico incluye a los recién nacidos clasificados con una "perforación gastrointestinal focal" si cumplen con los criterios anteriores. S  Criterios de exclusión: los lactantes con perforación gastrointestinal focal no asociada con EN clínica.  MBPN: <1500g | Resultado | 2 | 1 | 2 | 15 |
|  | Tasa de leucomalacia periventricular | Número de niños con muy bajo peso al nacer (MBPN) diagnosticados de leucomalacia periventricular | Todos los ingresos de niños con muy bajo peso al nacer con neuroimagen realizada en la unidad de cuidado intensivo neonatal | Evidencia de leucomalacia periventricular indicada por múltiples quistes pequeños periventriculares mostrada por cualquier imagen neural (ecografía craneal, TAC o RM). La ecogenicidad periventricular sin quistes no debe codificarse como LPV. Un quiste porencefálico en el área de hemorragia intraparenquimatosa previamente identificada no se codifica como LPV. Las anomalías periventriculares en la TAC o la RM no se codifican como LPV a menos que se identifiquen múltiples quistes periventriculares pequeños.  MBPN: <1500g | Resultado | 2 | 1 | 2 | 15 |
|  | Tasa de retinopatía de prematuro grado >2 | Número de niños con muy bajo peso al nacer (MBPN), <30 semanas de edad gestacional al nacer, diagnosticados de retinopatía del prematuro en grado >2 | Niños con MBPN supervivientes y con >30 semanas de edad gestacional al nacer con un examen de ojo | Grado 2: Presencia de cresta intrarretinal; Grado 3: Presencia de cresta con proliferación fibrovascular extrarretiniana; Grado 4: Desprendimiento parcial de retina; y Grado 5: Desprendimiento total de retina.  MBPN: <1500g | Resultado | 2 | 1 | 2 | 15 |
|  | Prevalencia de anomalías congénitas seleccionadas | Número de defectos del tubo neural, hendiduras orales y síndrome de Down | Nacidos vivos, muertes fetales y abortos inducidos | Indicador expresado como proporción.  Anencefalia y anomalías similares; (CIE10-Q00), Espina bífida (CIE10-Q05) Todas las anomalías congénitas del sistema nervioso central (CIE10-Q00-Q07); Síndrome de Down (CIE10-Q90); Labio leporino (CIE10-Q36); Paladar hendido con labio leporino (CIE10-Q37); y Paladar hendido (sin labio leporino) (ICD10-Q35). | Resultado | 2 | 3 | 3 | 13 |
|  | Tasa de neumotórax en la unidad de cuidado intensivo neonatal (UCIN) | Número de niños con muy bajo peso al nacer (MBPN) diagnosticados con neumotórax | Niños de muy bajo peso al nacer ingresados en UCIN | Neumotórax incluye: aire extrapleural diagnosticado por radiografía de tórax o por aspiración con aguja (toracocentesis) en hospital; también el diagnosticado para lactantes sometidos a cirugía torácica y que luego desarrollaron aire extrapleural diagnosticado por radiografía de tórax o toracocentesis con aguja.  Criterios de exclusión: lactantes que se sometieron a cirugía torácica y se colocó un tubo torácico en el momento de la cirugía, o si el aire libre sólo estaba presente en una radiografía de tórax inmediatamente después de una cirugía torácica y no se trató con un tubo torácico.  MBPN: <1500g | Resultado | 2 | 1 | 2 | 20 |
|  | Tasa de mortalidad neonatal por edad gestacional, peso al nacer y pluralidad | Número de muertes neonatales (0 a 27 días de edad) después de haber nacido vivo | Nacidos vivos calculados por edad gestacional, peso al nacer y pluralidad | Indicador expresado por 1000 nacidos vivos  Edad gestacional: 22-23 semanas, 24-27 semanas, 28-31 semanas, 32-36 semanas y, ≥ 37 semanas.  Peso al nacer: <500gr, 500-999gr, 1000-1499gr, 1500-2499 gr and, $\geq$2500 gr.  La pluralidad es un parto múltiple, como resultado de un embarazo único.  Esta tasa está subdividida por el momento de la muerte en: muertes neonatales tempranas (0-6 días de vida); y muertes neonatales tardías (7-27 días). | Resultado | 3 | 3 | 3 | 13 |
|  | Tasa de mortalidad temprana | Número de muertes neonatales entre 0-7 días | Número total de nacidos vivos | Por 1000 nacidos vivos | Resultado | 3 | 3 | 3 | 24 |
|  | Tasa de mortalidad tardía | Número de muertes neonatales entre 8-27 días | Número de nacidos vivos | Por 1000 nacidos vivos | Resultado | 3 | 3 | 3 | 24 |
|  | Tasa de mortalidad neonatal (NMR) | Número de muertes neonatales | Número de nacidos vivos | Por 1000 nacidos vivos.  Muerte neonatal definida dentro de los primeros 28 días de vida (0-27 días) | Resultado | 3 | 3 | 3 | 24 |
|  | Porcentaje de nacidos vivos infectados con VIH de madres infectadas | Número de nacidos vivos infectados con VIH de madres infectadas | Número total estimado de embarazadas infectadas de VIH | Expresado como porcentaje | Resultado | 3 | 3 | 1 | 24 |
|  | Porcentaje de nacidos vivos, en parto único de mujer multípara, con bajo peso, | Número de nacidos vivos, en parto único de mujer con dos o más nacimientos, con bajo peso (<2500g) | Número total de nacidos vivos, en parto único, de mujeres con dos o más nacimientos | Expresado como porcentaje.  Bajo peso al nacer (BPN) se define como peso menor de 2500 g en 24 horas de su nacimiento, sin importar la edad gestacional.  "Nacido vivo" es el nacimiento de un recién nacido, independientemente de la duración de la gestación, que presenta algún signo de vida, tales como la respiración, del latido del corazón, pulsación umbilical o movimiento de los músculos voluntarios. | Resultado | 3 | 3 | 1 | 24 |
|  | Porcentaje de nacidos vivos, en parto único de mujer primigesta, con bajo peso | Número nacidos vivos, en parto único de mujer con el primer nacimiento, con bajo peso <2500g | Número total de nacidos vivos, en parto único, de mujer con el primer nacimiento | Expresado como porcentaje.  Bajo peso al nacer (BPN) se define como peso menor de 2500 g en 24 horas de su nacimiento, sin importar la edad gestacional.  "Nacido vivo" es el nacimiento de un recién nacido, independientemente de la duración de la gestación, que presenta algún signo de vida, tales como la respiración, del latido del corazón, pulsación umbilical o movimiento de los músculos voluntarios | Resultado | 3 | 3 | 1 | 24 |
|  | Porcentaje de mujeres con un nacido vivo que reportó buscar atención de un proveedor calificado para un recién nacido enfermo | Número de mujeres con un nacido vivo que reportaron buscar atención de un proveedor calificado para un recién nacido enfermo | Número total de mujeres con un nacido vivo durante un periodo específico de tiempo | Expresado como porcentaje.  Un recién nacido enfermo se define como tener una enfermedad recién notificada en los primeros 28 días después del nacimiento.  Proveedores calificados pueden incluir los médicos privados, enfermeras, asistentes médicos, parteras y asistentes de parto capacitados y farmacéuticos. | Resultado | 3 | 3 | 3 | 24 |
|  | Tasa de mortalidad durante hospitalización en la unidad de cuidado intensivo neonatal (UCIN) | Número de muertes de niños con muy bajo peso al nacer (MBPN) antes de los 28 días de vida | Todos los ingresos en UCIN de niños con MBPN | Muertes de niños de MBPN antes de los 28 días de vida, esta medida incluye a bebés de MBPN nacidos fuera del hospital. Este indicador excluye las muertes antes de las 12 horas de vida  MBPN: <1500g | Resultado | 2 | 1 | 2 | 15 |
| **Otros** (más de una fase) | Proporción de profesionales de la salud que utilizaron doble guante en la atención a una mujer con una enfermedad de transmisión sanguínea | Profesionales de la salud que utilizaron doble guante | Profesionales de la salud que realizaron procedimientos quirúrgicos en una mujer con una enfermedad o afección de transmisión sanguínea | Enfermedad o afección de transmisión sanguínea puede ser bacteriana (hepatitis B o C) o viral (VIH) | Proceso | 2 | 2 | 3 | 3 |
|  | Tasa de control prenatal y postnatal oportunos | Control prenatal: nacidos vivos en los cuales las mujeres recibieron control prenatal en el primer trimestre de gestación o en los primeros 42 que se adscribió a la institución.  Control postnatal: Nacidos vivos en los cuales las mujeres recibieron atención postparto entre 21 y 56 días después del parto | Nacidos vivos en el año de medición | Indicador expresado como porcentaje  Si una mujer da a luz 2 veces en un año, cuenta para ambos partos. Sin embargo, una mujer con parto múltiple sólo debe contar una vez. | Proceso | 1 | 2 | 2 | 8 |
|  | Transfusión sanguínea durante y/o después del parto | Número de mujeres que recibieron transfusiones de sangre durante y / o después del parto | Número total de mujeres que dieron a luz | -- | Resultado | 2 | 3 | 2 | 18 |
|  | Incidencia de morbilidad materna grave | Morbilidad aguda grave resultante durante el embarazo, el parto o el puerperio (<42 días) | Mujeres que dan a luz nacidos vivos o nacidos muertos | Indicador expresado como proporción  Las morbilidades agudas graves se definen como las siguientes: 1.Eclampsia: incluye convulsiones tras trastornos hipertensivos especificados o no especificados (que no son debidos a epilepsia desconocida) durante el embarazo, el parto o el puerperio (CIE-10 O150). 2.Histerectomía: extirpación quirúrgica del útero -parcial o total, del cuerpo y/o del cuello uterino- para detener la hemorragia postparto no tratable) o embolización (proceso por el cual un vaso sanguíneo está obstruido por el depósito de una masa material -o un émbolo- para detener la hemorragia obstétrica grave). 3). Transfusión de sangre (todos los actos o procesos de transferencia de sangre a la vena, incluida la transfusión de glóbulos rojos, plaquetas (trombocitos) y plasma fresco congelado). y 4. Unidad de cuidados intensivos >24 horas (admisión durante el embarazo, el parto o el puerperio a cualquier establecimiento o unidad que proporcione cuidados intensivos o agudos o reanimación -tanto dentro como fuera de la unidad de maternidad- durante más de 24 horas) | Resultado | 3 | 3 | 3 | 13 |
|  | Razón intrahospitalaria de mujeres con condiciones amenazantes para la vida | Número de mujeres con afecciones amenazantes para la vida que desarrollaron estas condiciones en el hospital | Número de nacidos vivos en el hospital | Indicador expresado por 100 nacidos vivos  Las mujeres con afecciones potencialmente mortales o amenazantes para la vida: La suma de near-miss maternos (mujeres que casi murieron, pero sobrevivieron a una complicación que ocurrió durante el embarazo, el parto o hasta 42 días post-parto) y muertes maternas (muertes de mujeres durante el embarazo o dentro de los 42 días posteriores a la interrupción del embarazo o su atención, pero no por causas accidentales o incidentales) | Resultado | 3 | 2 | 1 | 11 |
|  | Razón de mujeres con condiciones amenazantes para la vida | Número de mujeres con afecciones amenazantes para la vida | Número de nacidos vivos | Indicador expresado por 100 nacidos vivos | Resultado | 3 | 3 | 1 | 11 |
|  | Razón de incidencia de *near-miss* materna | Número de casos de *near-miss* maternas | Número de nacidos vivos | Indicador expresado por 1000 nacidos vivos  Casos de *near-miss* (MNM): mujeres que casi murieron, pero sobrevivieron a una complicación que ocurrió durante el embarazo, el parto o hasta 42 días post-parto.  Nacido vivo: expulsión o extracción completa del producto de la concepción de la madre, mismo que respira o muestra cualquier otra evidencia de vida. Cada producto de tal nacimiento se considera nacido vivo | Resultado | 3 | 2 | 1 | 11 |
|  | Necesidad satisfecha de atención obstétrica (EmOC) | Número de mujeres tratadas por complicaciones obstétricas directas en servicios EmOC, en un periodo definido | Número estimado de mujeres con complicaciones obstétricas mayores (o 15% de los nacimientos esperados, durante el mismo periodo) | Expresado como porcentaje.  Las complicaciones obstétricas mayores son: Hemorragia: anteparto, intraparto o postparto; parto prolongado/obstruido; sepsis posparto; complicaciones de aborto; severa pre-eclampsia/eclampsia: embarazo ectópico y ruptura de útero.  El número de mujeres con una complicación obstétrica mayor incluye tanto a las mujeres admitidas con la complicación como a las mujeres que desarrollan la complicación en la instalación.  El EmOC incluye los niveles básicos e integrales de atención obstétrica esencial. | Resultado | 3 | 2 | 3 | 24 |
|  | Tasa de mortalidad intraparto y neonatal muy temprana | Número de nacidos con peso ≥ 2.5 kg que resultaron en muerte intraparto y muerte neonatal muy temprana, en primeras 24 horas | Número total de mujeres que dan a luz a bebés que pesen ≥ 2.5 kg durante el tiempo especificado | Expresado como porcentaje.  Mortalidad intraparto o neonatos muertos frescos incluyen a los niños nacidos muertos después de 28 semanas de gestación, sin deterioro de la piel o maceración. Se supone que la muerte se ha producido menos de 12 horas antes del nacimiento y excluye a los bebés con anomalías congénitas graves, letales. Muertes neonatales tempranas incluyen a neonatos nacidos a término que podrían no ser resucitados, pero la reanimación no estaba disponible, o que tenían un trauma de nacimiento específico, donde la muerte se produjo dentro de las 24 horas del nacimiento. | Resultado | 3 | 3 | 3 | 24 |
|  | Porcentaje de muertes fetales y neonatales por anomalías congénitas | Número de muertes en el periodo fetal y neonatal atribuidas a anomalías congénitas | Total de nacimientos | Muerte fetal: muerte en o después de 22 semanas completas de gestación. Muerte neonatal: muerte desde el día 0 al 27 tras el parto.  Anencefalia y anomalías similares; (CIE10-Q00), Espina bífida (CIE10-Q05) Todas las anomalías congénitas del sistema nervioso central (CIE10-Q00-Q07); Síndrome de Down (CIE10-Q90); Labio leporino (CIE10-Q36); Paladar hendido con labio leporino (CIE10-Q37); y Paladar hendido (sin labio leporino) (ICD10-Q35) | Resultado | 3 | 3 | 3 | 13 |
|  | Tasa de mortalidad feto-infantil específica del peso al nacer para infantes con muy bajo peso al nacer | Número total de muertes feto-infantiles en niños con peso al nacer de 500-1499 gramos | Número total de nacimientos (partos vivos y nacidos muertos) en niños con peso al nacer de 500-1499 gramos | Muy bajo peso al nacer: 500-1499 gramos Muerte feto-infantil: edad gestacional ≥20 semanas and ≥28 días de nacido | Resultado | 3 | 3 | 2 | 16 |
|  | Tasa de mortalidad proporcional | Número total de muertes feto-infantiles en cada una de las categorías de intervención | Número total de muertes feto-infantes | Indicador expresado por 1000 muertes feto-infantiles  Categoría 1: incluye Muerte Fetal (≥20 semanas); Muerte neonatal precoz (<7 días); Muerte Neonatal Tardía (7-27 días); Muerte post neonatal (28 días o más) y peso al nacer de 500 a 1.499 gramos; Categoría 2: incluye muerte fetales (≥20 semanas) y cualquier peso al nacer superior a 1.499 gramos;  Categoría 3: incluye muertes neonatales tempranas (<7 días) y peso de 1500-2499 gr y ≥ 2500 gr; y muertes neonatales tardías (de 7 a 27 días) y peso al nacer de 1.500 a 2.499 gramos;  Categoría 4:, incluye muertes neonatales tardías (de 7 a 27 días) y peso al nacer superior a 2.499 gramos así como muertes post neonatales (después de 28 días) y cualquier peso al nacer superior a 1.499 gramos | Resultado | 3 | 3 | 2 | 16 |
|  | Exceso en la tasa de mortalidad feto-infantil para cada categoría de intervención | 1. Número de muertes feto-infantiles ≥ 20 semanas de gestación en cada una de las categorías de intervención en la población de referencia  2. Número de muertes feto-infantiles ≥ 20 semanas de gestación en cada una de las categorías de intervención en la población de interés | 1. Número total de muertes feto-infantes en la población de referencia  2. Número total de muertes feto-infantes en la población de interés | Indicador expresado por 1000 muertes feto-infantiles en cada población.  Se resta la tasa de mortalidad proporcional para la población de referencia de la tasa de mortalidad proporcional de la población de interés (p.ej. la población con la tasa más baja de mortalidad feto-infantil) | Resultado | 3 | 3 | 2 | 16 |
|  | Tasa de mortalidad fetal | Número de fetos y bebés nacidos al año sin ninguna señal de vida y que nacieron después de 28 semanas de gestación o peso ≤1000 g | Total de nacimientos | Por 1000 nacimientos | Resultado | 3 | 2 | 3 | 25 |
|  | Tasa de mortalidad feto-infantil | Número de muertes fetales y nacidos vivos con ≥ 20 semanas de gestación por categoría de peso al nacer y edad al morir | Total de nacimientos | Indicador expresado por 1000 nacimientos  Peso al nacer (500-999 gr, 1000-1499 gr, 1500-2499 gr, ≥ 2500 gr).  Edad al morir: muerte Fetal (≥20 semanas); muerte neonatal precoz (<7 días); muerte Neonatal Tardía (7-27 días); muerte post neonatal (28 días o más). | Resultado | 3 | 3 | 2 | 16 |
|  | Tasa de mortalidad fetal por edad gestacional, peso al nacer y pluralidad | Número de muertes fetales en o después de 22 semanas completas de gestación | Nacidos vivos y nacidos muertos en el mismo año calculados por edad gestacional, peso al nacer y pluralidad. | Indicador expresado por 1000 nacidos muertos  Edad gestacional: 22-27 semanas, 28-31 semanas, 32-36 semanas, and $\geq$ 37 semanas.  Peso al nacer: <1000gr, 1000-1499gr, 1500-2499 gr, and $\geq$2500 gr.  La pluralidad se define como un parto múltiple, como resultado de un embarazo único | Resultado | 3 | 3 | 3 | 13 |
|  | Razón de mortalidad materna | Número de muertes maternas en una población | Número de nacidos vivos | Indicador expresado por 100 000 nacidos vivos  Una muerte materna se refiere a la muerte de una mujer por cualquier causa relacionada o agravada por el embarazo o su tratamiento (excluidas las causas accidentales o incidentales) durante el embarazo y el parto o dentro de los 42 días posteriores al embarazo, independientemente de la duración y el lugar del embarazo | Resultado | 3 | 3 | 1 | 12 |
|  | Razón de mortalidad: near-miss materna | Número de casos de near-miss maternas | Número de muertes maternas | Indicador expresado en proporción entre los casos de near-miss materna(MNM) y muertes maternas (MM).  [MNM : 1 MM]. | Resultado | 3 | 2 | 1 | 11 |
|  | Tasa de letalidad | Número de muertes maternas | Número de mujeres con afecciones amenazantes para la vida | Indicador expresado en porcentaje | Resultado | 3 | 2 | 1 | 11 |
|  | Tasa de letalidad-todas las complicaciones | Número de muertes por complicaciones obstétricas específicas en el establecimiento | Número de mujeres con complicaciones obstétricas específicas atendidas en el establecimiento | Expresado como porcentaje.  Donde se incluyen las muertes de las siguientes complicaciones: hemorragia: anteparto, intraparto o postparto; parto prolongado/obstruido; sepsis posparto; complicaciones de aborto; preeclampsia/eclampsia: embarazo ectópico y ruptura de útero. | Resultado | 3 | 3 | 1 | 24 |
|  | Tasa de mortalidad materna institucional | Número de muertes maternas en algún establecimiento de salud | Número total de partos en establecimientos de salud | Por 100 000 partos en establecimientos de salud | Resultado | 3 | 2 | 3 | 26 |
|  | Índice de mortalidad intra-hospitalaria | Número de muertes maternas intrahospitalarias | Número de mujeres con condiciones amenazantes para la vida que no estaban enfermas a su llegada al establecimiento de salud | Indicador expresado en porcentaje | Resultado | 3 | 2 | 1 | 11 |
| *Nivel de atención: 1. Primer nivel de atención; 2: Atención especializada (segundo o tercer nivel de atención); 3: Todos los niveles de atención*  *Nivel de aplicación: 1. Servicio o área específica del establecimiento de salud; 2. Establecimiento de salud; 3. Supra-institucional (región, estado o país)*  *Nivel de ingreso: 1. Bajo y medio ingreso; 2. Alto ingreso; 3. Alto, medio y bajo ingreso (todos)* | | | | | | | | | |

**References**

1. National Quality Forum (NQF). NQF #1391. <http://www.qualityforum.org/QPS/MeasureDetails.aspx?standardID=1391&print=0&entityTypeID=1>. Accessed 22 May 2016.

2. National Institute for Health and Care Excellence (NICE). Standards and Indicators. Single menu of indicators. 2016. [https://www.nice.org.uk/standards-and-indicators. Accessed 15 Jun 2016](https://www.nice.org.uk/standards-and-indicators.%20Accessed%2015%20Jun%202016).

3. Bonfill X, Roqué M, Aller MB, Osorio D, Foradada C, Vives A, et al. Development of quality of care indicators from systematic reviews: the case of hospital delivery. Implement Sci. 2013;8:42.

4. National Quality Forum (NQF). NQF #0473. <http://www.qualityforum.org/QPS/MeasureDetails.aspx?standardID=294&print=0&entityTypeID=1>. Accessed 22 May 2016.

5. National Quality Forum (NQF). NQF #0472. <http://www.qualityforum.org/QPS/MeasureDetails.aspx?standardID=293&print=0&entityTypeID=1>. Accessed 22 May 2016.

6. National Quality Forum (NQF). NQF #1746. http://www.qualityforum.org/QPS/MeasureDetails.aspx?standardID=1746&print=0&entityTypeID=1. Accessed 22 May 2016.

7. National Quality Forum (NQF). NQF #0475. http://www.qualityforum.org/QPS/MeasureDetails.aspx?standardID=287&print=0&entityTypeID=1. Accessed 22 May 2016.

8. National Quality Forum (NQF). NQF #1517. http://www.qualityforum.org/QPS/MeasureDetails.aspx?standardID=1517&print=0&entityTypeID=1. Accessed 22 May 2016.

9. Sprague AE, Dunn SI, Fell DB, et al. Measuring quality in maternal-newborn care: developing a clinical dashboard. J Obstet Gynaecol Canada JOGC = J d’obstétrique gynécologie du Canada JOGC. 2013;35(1):29-38.

10. National Quality Forum (NQF). NQF #1354. http://www.qualityforum.org/QPS/MeasureDetails.aspx?standardID=1354&print=0&entityTypeID=1. Accessed 22 May 2016.

11. Nelissen EJT, Mduma E, Ersdal HL, Evjen-Olsen B, van Roosmalen JJM, Stekelenburg J. Maternal near miss and mortality in a rural referral hospital in northern Tanzania: a cross-sectional study. BMC Pregnancy Childbirth. 2013; doi:10.1186/1471-2393-13-141

12. Countdown to 2015, Health Metrics Network, UNICEF and World Health Organization. Monitoring maternal, newborn and child health: understanding key progress indicators. Geneva: World Health Organization; 2011.

13. Euro-Peristat. Indicators. EURO-PERISTAT INDICATORS OF PERINATAL HEALTH, 2012. 2012. http://www.europeristat.com/images/doc/updated indicator list.pdf. Accessed 7 Mat 2016.

14. World Health Organization. Consultation on improving measurement of the quality of maternal, newborn and child care in health facilities. World Health Organization. 2014.

15. Kowalkowski M, Gould JB, Bose C, Petersen LA, Profit J. Do practicing clinicians agree with expert ratings of neonatal intensive care unit quality measures? J Perinatol. 2012; doi:10.1038/jp.2011.199:

16. Dunlop AL, McCarthy BJ, Freymann GR, Smith CK, Bugg GW, Brann AW. Analysis of feto-infant mortality using the BABIES framework: Georgia 1981-83 through 2001-03. Int J Heal Res. 2010;3(3):153-163.

17. Otsea K, Benson J, Alemayehu T, Pearson E, Healy J. Testing the Safe Abortion Care model in Ethiopia to monitor service availability, use, and quality. Int J Gynaecol Obstet. 2011; doi:10.1016/j.ijgo.2011.09.003

18. Boulkedid R, Sibony O, Goffinet F, Fauconnier A, Branger B, Alberti C. Quality indicators for continuous monitoring to improve maternal and infant health in maternity departments: a modified Delphi survey of an international multidisciplinary panel. PLoS One. 2013; doi:10.1371/journal.pone.0060663

19. Agency for Healthcare Research and Quality (AHRQ). NQMC #010343:010796. <https://www.qualitymeasures.ahrq.gov/summaries/summary/49434?search=neonatal>. Accessed 22 May 2016.

20. Profit J, Gould JB, Zupancic JAF, et al. Formal selection of measures for a composite index of NICU quality of care: Baby-MONITOR. J Perinatol. 2011; doi:10.1038/jp.2011.12

21. National Quality Forum (NQF). NQF #0304. http://www.qualityforum.org/QPS/MeasureDetails.aspx?standardID=266&print=0&entityTypeID=1. Accessed 22 May 2016.

22. Santos J V, Correia C, Cabral F, Bernardes J, Costa-Pereira A, Freitas A. Should European perinatal indicators be revisited? Eur J Obstet Gynecol Reprod Biol. 2013; doi:10.1016/j.ejogrb.2013.05.017

23. eCQI Resource Center. Healthy Term Newborn. <https://ecqi.healthit.gov/ecqm/measures/cms185v4> Accessed 15 Jul 2016.

24. MEASURE Evaluation. Family Planning and Reproductive Health Indicators Database. Summary List of Indicators. https://www.measureevaluation.org/prh/rh_indicators/indicator-summary. Accessed 26 Jul 2018.

25. Every Woman Every Child. Indicator and monitoring framework for the global strategy for women’s, children’s, and adolescents’ health (2016–2030). Geneva: World Health Organization; 2016.

26. World Health Organization. Global Reference List of 100 Core Health Indicators. Geneva; 2015. http://apps.who.int/iris/bitstream/handle/10665/173589/WHO_HIS_HSI_2015.3_eng.pdf?sequence=1. Accessed 9 Jun 2016.
